# Esketamine Provides Neuroprotection After Intracerebral Hemorrhage in Mice via the NTF3/PI3K/AKT Pathway

**DOI:** 10.1111/cns.70145

**Published:** 2024-12-17

**Authors:** Xiaoyu Niu, Yuanyuan Zheng, Wang Wang, Liwei Zhang, Shaoshuai Wang, Xihua Lu, Junyang Wang, Gaiqing Yang, Ting Zhao, Qiang Li, Nan Li, Junmin Wang, Jian Wang, Changsheng Li

**Affiliations:** ^1^ Department of Anesthesiology The Affiliated Cancer Hospital of Zhengzhou University Zhengzhou China; ^2^ Henan Provincial Key Laboratory of Radiation Medicine The First Affiliated Hospital of Zhengzhou University Zhengzhou China; ^3^ Department of Radiation Oncology The First Affiliated Hospital of Zhengzhou University Zhengzhou China; ^4^ Department of Physiology and Neurobiology, School of Basic Medical Sciences Zhengzhou University Zhengzhou China; ^5^ Non‐Commissioned Officer School of Army Medical University Shijiazhuang China; ^6^ Department of Human Anatomy, School of Basic Medical Sciences Zhengzhou University Zhengzhou China; ^7^ Department of Neurology Zhengzhou Central Hospital, Zhengzhou University Zhengzhou China; ^8^ Department of Neurology People's Hospital of Zhengzhou University Zhengzhou China; ^9^ Department of Neurology Shanghai Gongli Hospital of Pudong New Area Shanghai China; ^10^ Department of Neurology The 2nd Affiliated Hospital of Zhengzhou University Zhengzhou China; ^11^ Department of Anesthesiology The Third Affiliated Hospital of Zhengzhou University Zhengzhou China

**Keywords:** esketamine, intracerebral hemorrhage, neuroprotection, NTF3, PI3K/AKT signaling pathway

## Abstract

**Background:**

Esketamine (ESK), a noncompetitive antagonist of N‐methyl‐D‐aspartate (NMDA) receptors, modulates neurotransmitter signaling in the central nervous system. However, the specific mechanisms and therapeutic potential of ESK for intracerebral hemorrhage (ICH) remain unclear. This study aimed to investigate whether ESK promotes nerve repair and improves neurological outcomes in an experimental model of ICH.

**Methods:**

ICH was induced in mice via collagenase injection into the striatum. Body weight, neurological impairment, and behavioral changes were assessed. ESK administration significantly improved several indicators of ICH. Comprehensive RNA transcriptome sequencing and network pharmacology analyses identified neurotrophin‐3 (NTF3) and the PI3K/AKT signaling pathway as targets for ESK treatment. Western blotting and immunofluorescence detected the protein expression levels and cellular localization of NTF3.

**Results:**

After 28 days of adeno‐associated virus infection in the mouse striatum, ESK treatment significantly enhanced neuroprotection, indicating the crucial role of NTF3 in ESK‐mediated neuroprotection in ICH mice. Inhibition of the PI3K/AKT pathway using the PI3K‐specific inhibitor LY294002 significantly attenuated the therapeutic effects of ESK, suggesting that this pathway is involved in ESK‐mediated neurorepair in ICH mice.

**Conclusions:**

ESK treatment significantly improved functional outcomes and demonstrated neuroprotective effects in animal models of ICH. NTF3/PI3K/AKT pathway activation by ESK indicates its therapeutic potential in the treatment of ICH.

## Graphical abstract

Schematic representation of the underlying molecular mechanisms by which ESK mediates neuroprotection of the PI3K/AKT signaling pathway in mice with ICH through activation of NTF3 expression.

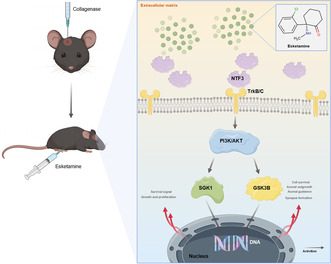

## 1 Introduction

Intracerebral hemorrhage (ICH) is a type of cerebral injury caused by the acute extravasation of blood into the brain parenchyma due to the rupture of a cerebral artery. It affects approximately 2.9 million people worldwide each year [1]. In the United States, ICH affects approximately 80,000 people annually, accounting for about 10%–15% of all strokes [2]. Despite recent preclinical and clinical trials, no treatment has significantly improved mortality or neurological outcomes after ICH. Therefore, new therapeutic approaches are urgently needed to enhance patient prognosis and promote post‐stroke rehabilitation and recovery.

Esketamine (ESK), an azocyclic compound and the S‐isomer of ketamine, is widely used as an analgesic, soothing, and anesthetic. As a potent N‐methyl‐D‐aspartate (NMDA) receptor antagonist, ESK plays a crucial role in the central nervous system, producing rapid sedative anesthetic effects through its action on glutamate receptors [3, 4]. Several studies have reported ESK's neuroprotective effects [5, 6]. However, its potential clinical applications, notably its neuroprotective role in ICH, remain underexplored.

Neurotrophin‐3 (NTF3 and NT‐3) is a member of the neurotrophic factor family. Nerve growth factors are prototypical members of this family and promote sympathetic and sensory neuron survival during development [7]. NTF3 performs several physiological functions in the nervous system, including neuronal survival and development, synaptic plasticity, nerve injury repair, and anti‐inflammatory and neuroprotective effects [8]. Increased NTF3 was shown to have a protective effect on cells and rats of ICH in a delivery system study [9].

As an NTF3‐mediated downstream pathway, the PI3K/AKT pathway is an important signaling pathway involved in various cellular processes, including cell proliferation, survival, differentiation, and metabolism [10]. PI3K phosphorylates multiple substrate proteins, such as glycogen synthase kinase 3 beta (GSK3B) and serum glucocorticoid‐regulated kinase 1 (SGK1), through the activation of protein kinase B (AKT). Several studies have shown that the PI3K/AKT pathway is closely associated with ICH [11, 12, 13].

We hypothesized that NTF3 activation promotes PI3K/AKT‐mediated neurotrophy‐related pathways after ICH and aimed to explore the functional role of ESK in ICH mice in this study.

## 2 Materials and Methods

Detailed information is provided in the Supporting Information, including animal groups (Figure S1 and Table S1) and statistical analysis (Table S6).

### 2.1 Animals

Male C57BL/6 mice (8–10 weeks; 20–30 g). All animal experiments were approved by the Animal Care Committee of Zhengzhou University (approval number: ZZUIRB 2022‐31), and we followed the updated ARRIVE (Animal Research: Reporting of In Vivo Experiments) 2.0 guidelines [14, 15].

### 2.2 ICH Mouse Model

Under stereotactic guidance, the mouse ICH model was established by injecting 0.075 U collagenase VII‐S into the right striatum. Mice in the sham group were injected with an equivalent volume of normal saline [16]. After randomization, ESK (Hengrui; Jiangsu, China; 50 mg/2 mL), LY294002 (MCE, USA; 10 nmol/2 μL or 8 mg/kg), and adeno‐associated virus (AAV2/9‐U6‐shRNANTF3‐WPRE; 2 μL; 1 × 10^13^ V.G/mL) were administered at appropriate dosages, routes, and times.

### 2.3 Weight and Behavioral Tests

Body weight was measured and recorded at fixed time points. The body weight ratio (%) was calculated as (body weight at a fixed time point/initial body weight) × 100%. Neurological function was assessed using the neurological deficit score, rotarod test, beam walking test, and grid walking test [17]. After ICH, behavioral assessments were conducted to determine the optimal drug dose on Days 1, 3, 7, and 14. To evaluate the effects on motor and neurological function following the acute phase of ICH, assessments were conducted on Days 1, 3, and 7 after ICH.

### 2.4 Molecular and Histological Analyses

Polymerase chain reaction (PCR) was used to detect AAV infection, and western blotting was used to assess protein expression. Histomorphological and cell activity changes in the brain were evaluated using immunofluorescence, Luxol fast blue/cresyl violet (LFB/CV) staining, hematoxylin and eosin (H&E) staining, terminal uridine nucleotide end labeling (TUNEL) staining, and Fluoro‐Jade C (FJC) staining. Brain water content was quantified using the dry‐wet method [14, 18].

### 2.5 Transcriptome Sequencing and Network Pharmacology Validation

Differentially expressed genes (DEGs), Kyoto Encyclopedia of Genes and Genomes (KEGG) pathways, and Gene Ontology (GO) data were analyzed to predict key factors and related pathways. Network pharmacology validation was used to mine ESK with ICH targets and construct a protein interaction network of ICH targets for ESK therapy.

### 2.6 Blood Sample Analysis

Blood samples from mice were evaluated to measure drug toxicity and related indices.

### 2.7 Statistical Analysis

Mice that died during surgery or shortly after ICH induction were excluded from the final analysis. The normality of data distribution was assessed using the Shapiro–Wilk test. Data are expressed as mean ± standard deviation. Unpaired *t*‐tests were used for comparisons between the two groups. Repeated‐measures analysis of variance (ANOVA) with Bonferroni post hoc correction was applied to data collected at different time points across groups. One‐way ANOVA with Bonferroni post hoc correction was used to compare multiple groups, while the Friedman or Kruskal–Wallis test was employed for non‐normally distributed data. Mortality rates were compared using the chi‐squared test. Statistical analyses were conducted with GraphPad Prism 9.5.0 (GraphPad Software, San Diego, CA, USA). Statistical significance was set at *p* < 0.05.

## 3 Results

### 3.1 Animal Mortality

A total of 323 mice were used in this study, of which 243 underwent ICH surgery. All mice in the sham group survived. The overall mortality rate of the mice with ICH was 8.36% (27/323) (Table S1).

### 3.2 Construction of the ICH Model and Determination of the Optimal Drug Dose

**FIGURE 1:**
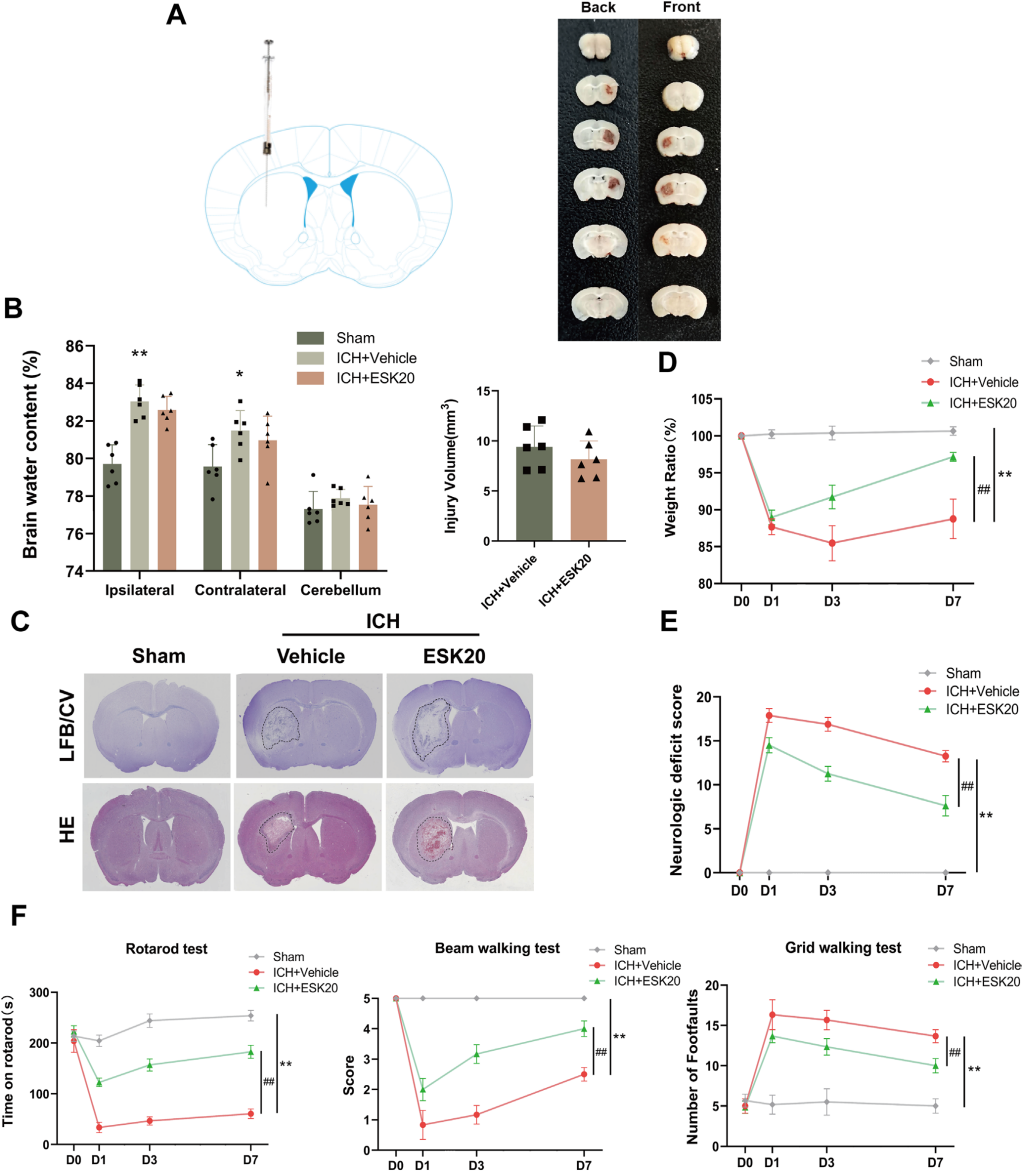
ESK treatment attenuates weight loss and improves neurological and motor function in mice with ICH. (A) ICH was induced in C57BL/6 mice via collagenase injection into the right striatum. (B) Different groups of brain water content on Day 3 after ICH and brain injury volume measurement according to Luxol fast blue/cresyl violet (LFB/CV) staining (*n* = 6). (C) Representative brain sections stained with LFB/CV and hematoxylin and eosin. (D) Body weight ratio. (E) Neurological deficit score. (F) Behavioral assessments using the rotarod test, beam walking test, and grid walking test. *p < 0.05, **p < 0.01: ICH + vehicle versus sham; ^#^ *p* < 0.05, ^##^ *p* < 0.01: ICH + ESK20 versus ICH + vehicle (*n* = 6). All data are mean ± SD.

We constructed an ICH model by injecting collagenase VII‐S into the striatum of mice and assessed the success using LFB/CV and H&E staining (Figure 1A,C). Compared to the sham group, mice in the ICH group showed significant neurological damage at different time points after surgery. We excluded in vivo studies in rodents at doses higher than 40 mg/kg (acute or chronic), due to the potential anesthetic, psychotomimetic, and neurotoxic effects of ESK at higher or repeated high doses [19]. To determine the optimal dosage, three doses of ESK (10, 20, and 40 mg/kg) were injected intraperitoneally at fixed times after ICH to assess their effects on body weight and neurological prognosis in ICH mice. The middle dose (20 mg/kg) significantly improved neurological deficits, body weight, and behavioral performance after ICH (Figure S2A–C). Therefore, we used 20 mg/kg as the optimal dose for subsequent experiments.

### 3.3 ESK Reduces Nerve Damage and Improves Motor Function After ICH in Mice

**FIGURE 2:**
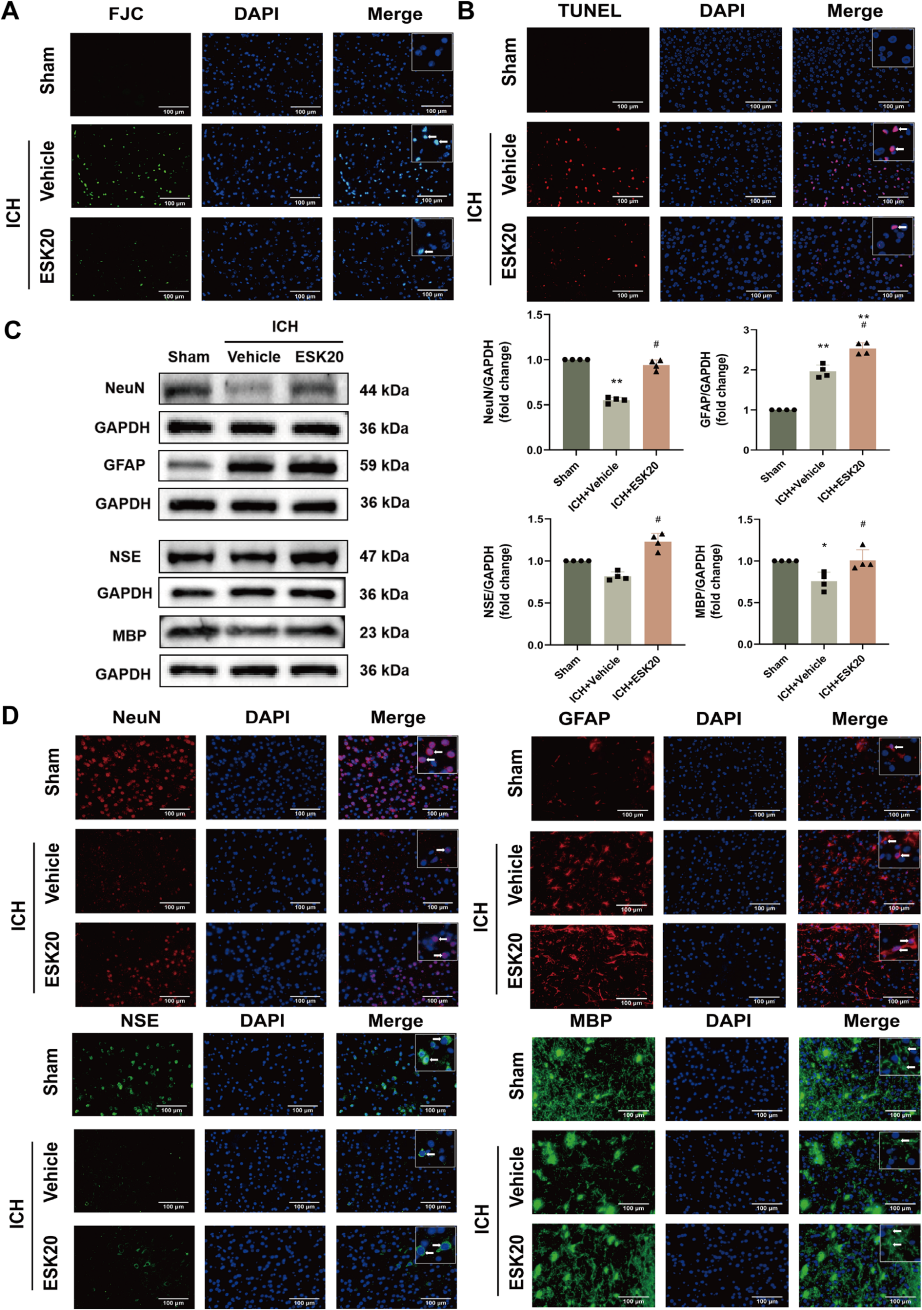
ESK treatment reduces neuronal degeneration and death while increasing the protein expression of NeuN, GFAP, NSE, and MBP in mice subjected to ICH. (A) Representative images of Fluoro‐Jade C staining in the perihematomal region 72 h after ICH (*n* = 3). (B) Representative images of TUBEL staining in the perihematomal region at 72 h after ICH (*n* = 3). (C) Western blot analysis of NeuN, GFAP, NSE, and MBP protein expression, showing representative bands (*n* = 4), *p < 0.05, **p < 0.01 versus sham; ^#^ *p* < 0.05, ^##^ *p* < 0.01 versus ICH + vehicle. (D) Immunofluorescence images of NeuN, GFAP, NSE, and MBP in the perihematomal region of mouse brain tissue after ICH (*n* = 3). Scale bar: 100 μm.

At a dose of 20 mg/kg, there was a significant improvement in body weight, neurological function, and behavioral changes in the ICH + ESK20 group compared to the ICH + vehicle group (Figure 1D–F). FJC and TUNEL staining revealed that the number of FJC‐positive neurons was significantly lower in the ICH + ESK20 group than in the ICH + vehicle group, indicating a significant reduction in nerve injury (Figure 2A,B). Immunofluorescence and western blot analysis of NeuN, glial fibrillary acidic protein (GFAP), neuron‐specific enolase (NSE), and myelin basic protein (MBP) in the ipsilateral striatum showed uniform staining in the same group. In contrast, the ICH group exhibited decreased expression of NeuN, NSE, and MBP, and increased expression of GFAP (Figure 2D). The treatment group, however, showed increased NeuN‐, GFAP‐, NSE‐, and MBP‐positive cells (Figure 2D). Western blot results corroborated these findings, showing higher NeuN, NSE, and MBP expression in the sham group (Figure 2C). The protein expression levels of NeuN, GFAP, NSE, and MBP were significantly higher in the ICH + ESK20 group than in the ICH + vehicle group. To evaluate the safety of ESK, we administered 20 mg/kg ESK to normal mice using the same method and recorded body weights, as well as neurological and motor functions, to confirm that ESK at this dose did not produce toxic side effects. These studies showed that a 20 mg/kg dose of ESK did not have any adverse effects (Figure S3A–C). Additionally, liver and kidney H&E staining, routine blood tests, and relevant liver and kidney biochemical indices of the dosed mice revealed no adverse effects of ESK at doses of 20 or 40 mg/kg (Figure S3D,E and Tables S4 and S5). Finally, the results showed that there was no significant difference in injury volume (mm^3^) or brain water content (%) between the ICH + ESK20 and ICH + vehicle groups (Figure 1B).

### 3.4 Transcriptome Sequencing Reveals Key Genes and Pathways in ESK‐Treated ICH Mice

**FIGURE 3:**
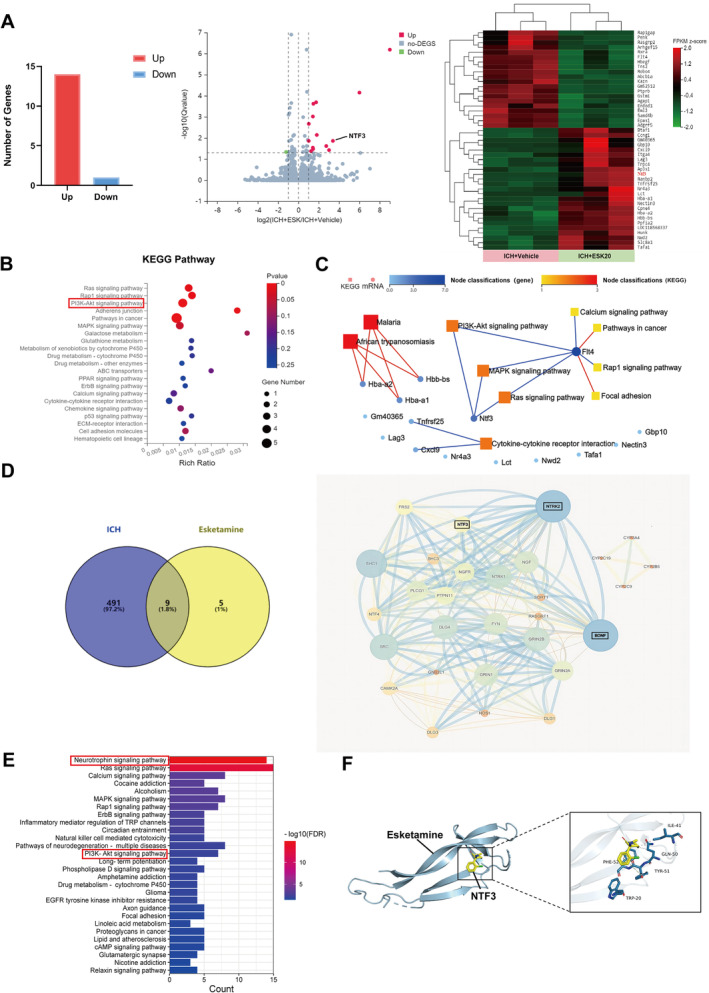
ESK treatment alters the gene transcription profile induced by ICH, with network pharmacology predicting possible target pathway interactions involved. (A) Histogram of differentially expressed genes (DEGs) comparing ICH + ESK20 vs. ICH + vehicle groups). Volcano plots show selected pairwise comparisons. DEGs were identified based on log2 |fold‐change| > 1 and Q‐value < 0.05. Heatmap of the cluster analysis illustrates transcript expression levels of DEGs in ICH groups on Day 3 after treatment with ESK or vehicle. The color scale indicates relative expression levels: red represents level above the mean, and green represents levels below the mean. (B) KEGG pathway enrichment bubble map, where a larger −log10 (*p*‐value) indicates a greater degree of enrichment. (C) Network of significantly enriched KEGG pathways altered by ESK treatment. Squares represent related pathways, circles represent DEGs, and darker colors indicate stronger correlations. (D) Venn diagram showing putative targets of ESK and ICH‐related genes, with a protein–protein interaction network illustrating interactions among target genes. Circle size indicates the target protein degree value. (E) Pathway enrichment analysis. (F) Molecular docking analysis predicts the binding of ESK to NTF3, showing hydrophobic interactions and π–π stacking with NTF3 residues that form a stable spatial conformation.

To gain a deeper understanding of the neuroprotective mechanism of ESK in ICH, we performed RNA‐seq to sequence the mRNA transcriptome of brain tissue surrounding the hematoma in the experimental and control groups. Transcriptome sequencing revealed that 15 genes were significantly altered in the ICH + ESK20 group compared with the ICH + vehicle group (Figure 3A and Table S2). KEGG pathway analysis suggested the enrichment of several important neurological, inflammatory, and immune pathways involved, such as the Ras, Rap1, PI3K/AKT, and MAPK signaling pathways (Figures 3B and S2D). GO analysis suggested the involvement of the axon, myelin sheath, and neuronal cell body in “Cellular Components” and differential gene regulation in “Biological Processes” including the nerve growth factor signaling pathway, nerve development, axon guidance, neuron development, brain development, nervous system development, and other processes related to neural regeneration and nerve growth factor receptor binding. (Figure S2E). KEGG pathway and differential gene coexpression analyses suggested that the association of NTF3 with the PI3K/AKT pathway was most relevant in the ICH + ESK20 group compared with the ICH + vehicle group (Figure 3C).

### 3.5 Network Pharmacological Analysis Indicates the Feasibility and Significance of Key Molecules and Associated Pathways

**FIGURE 4:**
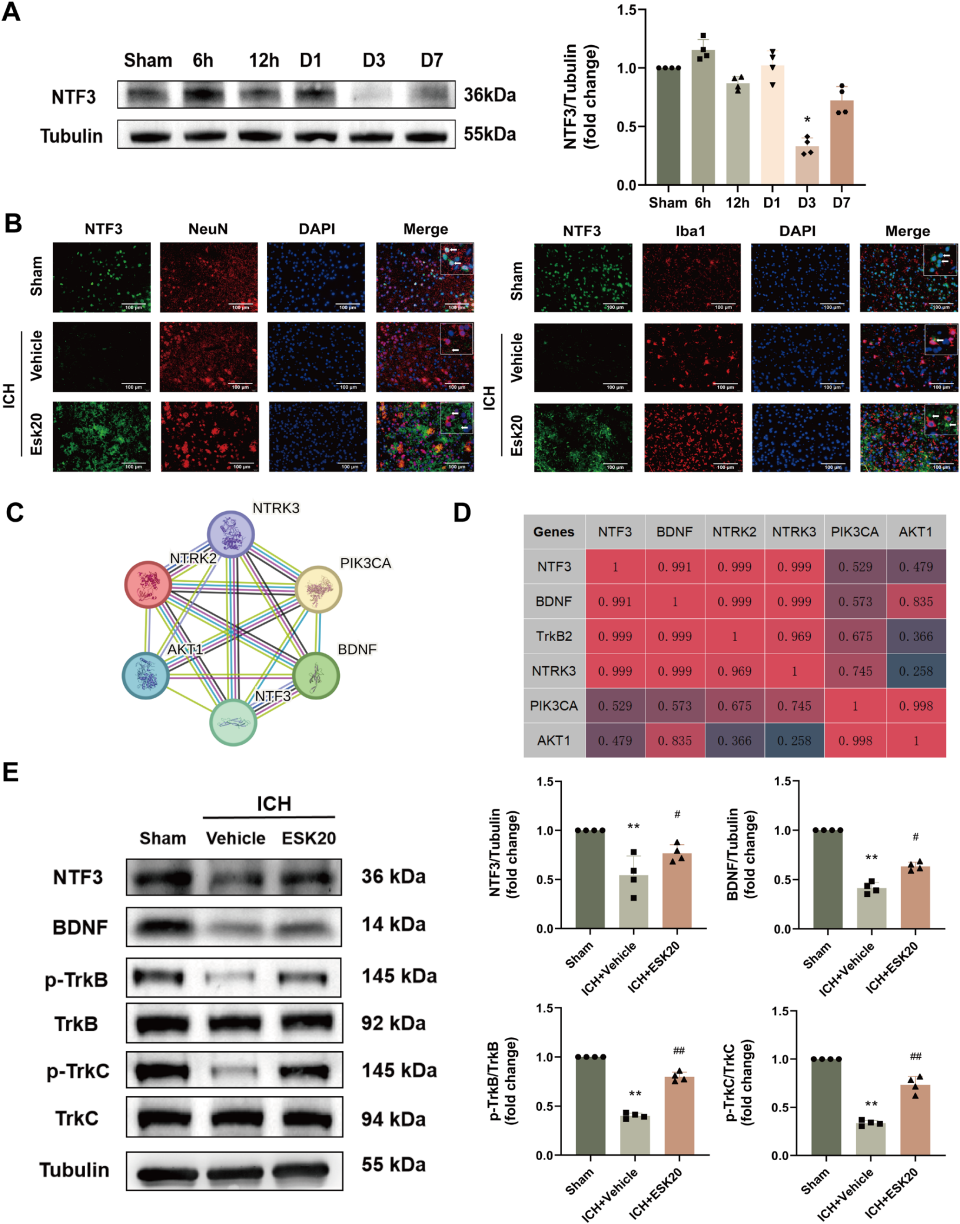
Time course of NTF3 protein levels and cellular localization after ICH. (A) Representative Western blot results and quantitative analyses of temporal changes in NTF3 expression in the ipsilateral hemisphere after ICH (*n* = 4). *p < 0.01 versus sham. (B) Representative images of double immunofluorescence staining showing colocalization of NTF3 (green) with neurons (NeuN, red) or microglia (Iba1, red) in the perihematomal region of ICH + vehicle and ICH + ESK20 groups, and the corresponding region in the sham group, at 72 h after ICH (*n* = 3). Scale bar: 100 μm. (C) NTF3‐related protein interaction network map, with circles representing proteins and lines, indicating verified interactions by different methods. (D) Correlation analysis between proteins. Scores range from 0 to 1, with higher scores associated with greater correlations. (E) Representative Western blot bands and quantitative analyses of the expression of key proteins associated with NTF3 function (*n* = 4). **p < 0.01 versus sham; ^#^ *p* < 0.05, ^##^ *p* < 0.01 versus ICH + vehicle.

We used network pharmacology to predict and validate ESK related targets and pathways for ICH treatment. Data mining from GeneCards, DrugBank, and other databases revealed nine common targets between ESK and ICH: BDNF, GRIN2B, GRIN1, NTRK2, CYP2C9, GRIN2A, CYP3A4, CYP2C19, and CYP2B6 (Figure 3D). Protein–protein interaction network analysis of these common targets revealed several significantly associated proteins, including NTF3, which we sequenced, with scores of 0.999 for BDNF, NTF3 with NTRK2, and 0.991 for BDNF with NTF3 (combined score), which showed the highest confidence (combined score ≥ 0.900 out of 1) (Figures 3D and 4D). Subsequent KEGG pathway analysis ranked the neurotrophic factor pathway as the most associated (FDRP < 0.01), with the PI3K/AKT signaling pathway also showing strong association (FDRP < 0.01) (Figure 3E and Table S3). Molecular docking also provides a reference for understanding the relationship between drugs and their target proteins. NTF3 (yellow) served as the molecular docking acceptor. Molecular docking of the receptor proteins was performed using AutoDock software for the ligand, ESK (blue). The affinity of ESK for the receptor protein NTF3 was less than −5 kcal/mol, indicating that the binding was more stable. The results showed that the interaction between ESK and NTF3 occurred mainly through hydrophobic interactions and π–π stacking of amino acid residues, which could form a stable spatial conformational structure. Network pharmacology validation reinforced the sequencing results and confirmed the drug's mechanism of action (Figure 3F). These findings suggest that ESK activates molecules, pathways, and functions related to neuroregeneration and neuroprotection following ICH. Based on this comprehensive analysis, we used NTF3 and the PI3K/AKT pathway as the target mechanism in our subsequent experiment. The downstream molecules GSK3B and SGK1, which play vital roles in the PI3K/AKT pathway, were also used as indicators of neurological damage and recovery following ICH.

### 3.6 Expression and Localization of NTF3 in ICH

After identifying the potential target molecules and pathways for ESK in the treatment of ICH, we further explored the role of NTF3. We detected the protein expression of NTF3 in the brain tissue of ICH mice using Western blotting at 6 and 12 h, and on the first, third, and seventh day after surgery. NTF3 expression was significantly lower on the third day after ICH in the ICH group compared to the sham group (*p* < 0.05; Figure 4A). Immunofluorescence staining for NTF3 in neurons and microglia showed a significant decrease in NTF3 protein expression on the third‐day post‐ICH in the ICH group compared to the sham group. After ESK treatment, diffuse NTF3 protein expression appeared in the extracellular matrix, potentially related to an increase and release of NTF3 protein from neurons (Figure 4B). Costaining with microglia showed similar results, although NTF3 was not colocalized with microglia (Figure 4B). Protein–protein interaction analysis using the STRING database demonstrated that proteins such as BDNF, TrkB, and TrkC interact with NTF3 and play critical roles in ICH (Figure 4C,D). We confirmed these interactions by analyzing the expression of proteins related to NTF3's role and its involvement in the pathway associated with ESK treatment of ICH using western blotting. The expression of the same family of star proteins, BDNF, was consistent with that of NTF3. The receptors, TrkB and TrkC, showed trends similar to NTF3. These findings suggest that BDNF may play a synergistic role with NTF3 in this process, and their receptors may also play a critical role (Figure 4E).

### 3.7 Effect of AAV‐shRNA‐NTF3 on ESK Treatment

**FIGURE 5:**
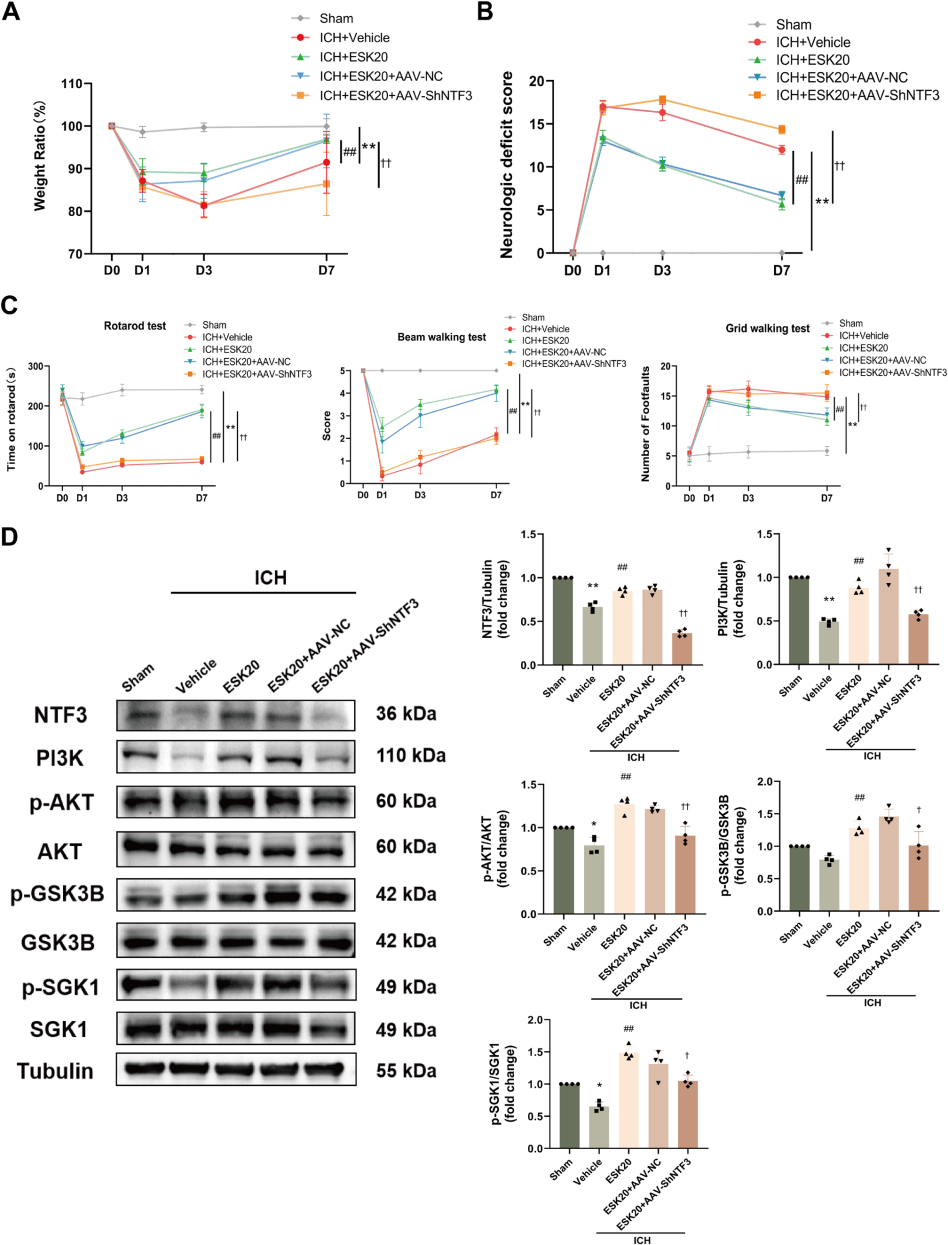
Effects of AAV treatment on body weight and neurological and motor function on Days 1, 3, and 7 after ICH in mice. (A) Body weight ratio. (B) Neurological deficit score. (C) Behavioral assessments (rotarod test, beam walking test, and grid walking test). *p < 0.05, **p < 0.01: ICH + vehicle versus sham; ^#^ *p <* 0.05, ^##^ *p <* 0.01: ICH + ESK20 versus ICH + vehicle; ^†^ *p <* 0.05, ^††^ *p <* 0.01: ICH + ESK20 + AAV‐ShNTF3 versus ICH + ESK20 (*n* = 6). (D) In each group, Western blot bands and analysis of NTF3, PI3K, p‐AKT, p‐GSK3B, and p‐SGK1 protein expression (*n* = 4). *p < 0.05, **p < 0.01 versus sham; ^##^ *p* < 0.01 versus ICH + vehicle; ^†^ *p* < 0.05, ^††^ *p* < 0.01 versus ICH + ESK20.

We silenced the NTF3 gene using AAV infection in specific brain regions of the mice. After 28 days of infection, NTF3 expression in these regions was significantly altered at both mRNA and protein levels, and the optimal shRNA sequence (AAV‐shRNA1) was identified (Figure S5A,B). Infection success was assessed by detecting fluorescence‐carrying viruses using immunofluorescence (Figure S5C,D). The results showed that the therapeutic effects on body weight, neurological function, and behavior were significantly reduced in mice treated with ESK following NTF3 silencing with AAV (**p < 0.01: ICH + vehicle vs. sham; ^#^ *p <* 0.05, ^##^ *p <* 0.01: ICH + ESK20 vs. ICH + vehicle; ^†^ *p <* 0.05, ^††^ *p <* 0.01: ICH + ESK20 + AAV‐ShNTF3 vs. ICH + ESK20), (Figure 5A–C). Western blotting revealed significant alterations in PI3K/AKT pathway‐related proteins levels after AAV‐shRNA‐NTF3 treatment. NTF3, PI3K, p‐AKT, and p‐SGK1 protein expression levels were significantly lower in the ICH + vehicle group than in the sham group (*p < 0.05, **p < 0.01; Figure 5D). Following ESK treatment, the expression levels of NTF3, PI3K, p‐AKT, p‐GSK3B, and p‐SGK1 were significantly higher in the ICH + ESK20 group than in the ICH + vehicle group (^##^ *p* < 0.01; Figure 5D), while NTF3 knockdown with AAV‐ShNTF3 resulted in significantly lower expression levels of NTF3, PI3K, p‐AKT, p‐GSK3B, and p‐SGK1 in the ICH + ESK20 group compared to the ICH + ESK20 + AAV‐ShNTF3 group (^†^ *p* < 0.05, ^††^ *p* < 0.01, Figure 5D). These results suggest that NTF3 is critical to the beneficial effects of ESK in ICH mice and that AAV‐shRNA‐NTF3 can impede ESK treatment.

### 3.8 PI3K Inhibitor, LY294002, Blocks ESK in ICH Mice

**FIGURE 6:**
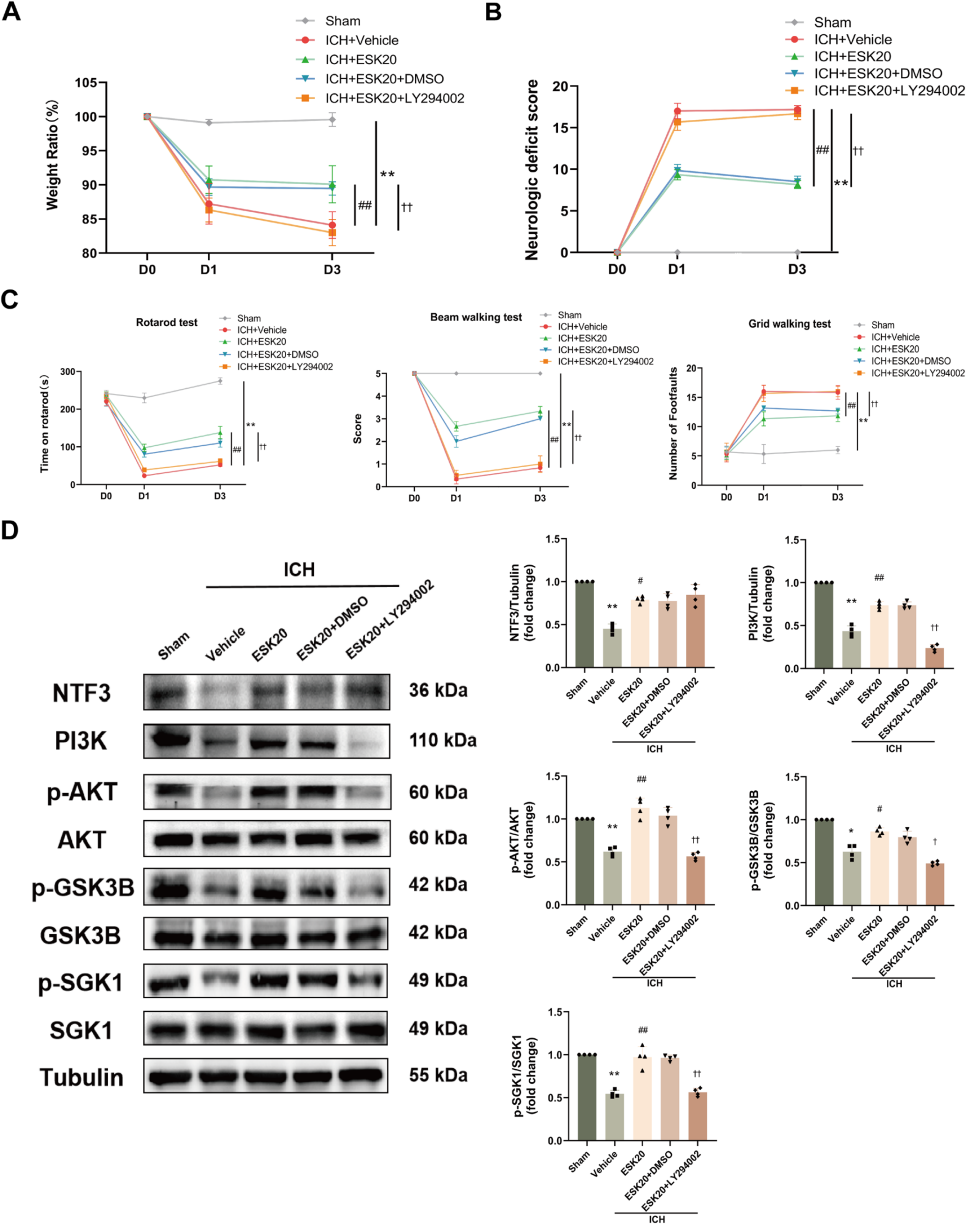
Effects of LY294002 treatment on body weight and neurological and motor function on Days 1 and 3 after ICH in mice. (A) Body weight ratio. (B) Neurological deficit score. (C) Behavioral assessments (rotarod test, beam walking test, and grid walking test). *p < 0.05, **p < 0.01: ICH + vehicle versus sham; ^#^ *p <* 0.05, ^##^ *p <* 0.01: ICH + ESK20 versus ICH + vehicle; ^†^ *p <* 0.05, ^††^ *p <* 0.01: ICH + ESK20 + LY294002 versus ICH + ESK20 (*n* = 6). (D) Results and analyses of each group's Western blot of NTF3, PI3K, p‐AKT, p‐GSK3B, and p‐SGK1 protein expression (*n* = 4). *p < 0.05, **p < 0.01 versus sham; ^#^ *p* < 0.05, ^##^ *p* < 0.01 versus ICH + vehicle; ^†^ *p* < 0.05, ^††^ *p* < 0.01 versus ICH + ESK20.

We used the PI3K inhibitor, LY294002, to block the PI3K/AKT pathway. The therapeutic effects on body weight, neurological function, and behavior were significantly diminished in mice treated with the inhibitor, followed by ESK treatment (**p < 0.01: ICH + vehicle vs. sham; ^#^ *p <* 0.05, ^##^ *p <* 0.01: ICH + ESK20 vs. ICH + vehicle; ^†^ *p <* 0.05, ^††^ *p <* 0.01: ICH + ESK20 + LY294002 vs. ICH + ESK20; Figure 6A–C). Western blotting showed that PI3K/AKT pathway‐related protein levels were significantly altered following LY294002 treatment. Protein expression levels of NTF3, PI3K, p‐AKT, p‐GSK3B, and p‐SGK1 were significantly lower in the ICH + vehicle group than in the sham group (*p < 0.05, **p < 0.01; Figure 6D). After ESK treatment, the expression levels of NTF3, PI3K, p‐AKT, p‐GSK3B, and p‐SGK1 were significantly higher in the ICH + ESK20 group than in the ICH + vehicle group (^#^ *p* < 0.05, ^##^ *p* < 0.01; Figure 6D); while these levels were significantly lower in the ICH + ESK20 + LY294002 group compared to the ICH + ESK20 group (^†^ *p* < 0.05, ^††^ *p* < 0.01, Figure 6D). These results suggest that the PI3K/AKT pathway is essential for the neuroprotective effects of ESK in ICH mice and that LY294002 can inhibit these effects.

## 4 Discussion

In this study, we found that the optimal dose of ESK reduced nerve damage, decreased neuronal cell death, and improved motor function in mice with ICH. NeuN, NSE, MBP, and GFAP protein expression levels were increased in the perihematomal tissue of ICH mice treated with ESK. Subsequent transcriptome sequencing and network pharmacology analyses identified a key target, NTF3. GO analysis suggested that “Biological Processes” of neuroprotection and neurorepair may exist in the ESK treatment group. KEGG pathway analysis also revealed the classical downstream pathway of NTF3. This PI3K/AKT pathway was experimentally validated to highlight the critical role of the NTF3 and PI3K/AKT signaling pathways in ESK treatment. The significant phosphorylation of the downstream effector molecules, GSK3B and SGK1, within the activated PI3K/AKT signaling pathway, indicated that the cerebral nerves of mice with ICH in the ESK treatment group were protected and repaired.

Since the nasal spray, esketamine (Spravato), was approved by the United States Food and Drug Administration for treating treatment‐resistant conditions on March 5, 2019, ESK has been approved in the United States, the United Kingdom, and Europe [20, 21] The sedative, analgesic, and neuroprotective effects of ESK have gained considerable interest in recent years [19]. Several studies have reported on the neuroprotective effects of ESK and its associated mechanistic pathways [22, 23]. Current studies have shown that the primary target of ESK is the glutamate/NMDA receptor (Protein Group) [24]. Other currently confirmed or potential targets of ESK includeGRIN2B [25], EEF2 [25], BDNF [25, 26], and the BDNF/NT‐3 receptor (NTRK2, TrkB) [25, 26]. Investigating these key targets may provide additional support for the potential efficacy and application of ESK. We also predicted and validated potential targets and pathways of ESK and ICH using network pharmacology, and the results were consistent with the evidence obtained from sequencing in the present study.

NeuN is commonly used as a marker to identify and study mature neuronal cells [27]. MBP is a structural protein mainly present in the myelin sheath of the central nervous system and is essential for nerve impulse conduction and axonal formation; cerebral hemorrhage destroys neuronal myelin [28]. GFAP is highly expressed in astrocytes in the central nervous system, and its expression can reflect the response of astrocytes, which are involved in supporting and repairing damaged areas and in immune regulation in ICH [29]. NSE is a hallmark protein of neural tissue, primarily present in neurons and neuroendocrine cells, and can indicate neuronal survival and axonal regeneration [22]. The expression levels of these specific proteins (NeuN, NSE, MBP, and GFAP) were further analyzed using western blotting and immunofluorescence staining, suggesting that ESK promotes the survival and proliferation of endogenous neural cells through the secretion of neurotrophic factors and neuronal repair.

As a member of the neurotrophin family, NTF3 plays a significant role in various processes, including nerve repair and regeneration, neuroprotection, and anti‐inflammation following neurological injury [8, 30, 31]. NTF3 binds to TrkB and TrkC receptors, activating the PI3K/AKT pathway. This canonical downstream signaling pathway further promotes the neurotrophin pathway [32]. The PI3K/AKT pathway has emerged as a critical focus for investigating the pathogenesis, treatment, and prognosis of ICH [33, 34, 35]. BDNF, another neurotrophin family number closely associated with the NTF3 and PI3K/AKT pathways, is also critical for neuronal development, growth, differentiation, and maintenance, including in studies on ICH [36]. Our previous research demonstrated that BDNF is essential for protecting sensorimotor function, and alleviating depression, anxiety‐like behaviors, and cognitive impairment after ICH [15, 37]. This study also suggests a strong association between NTF3 and BDNF, both of which have potential synergistic and reciprocal effects and may play a role in neuroprotection against ICH. In this study, we examined the changes in BDNF, PI3K, and p‐AKT protein levels at different time points after ICH using western blotting (Figure S4A).

GSK3B is a serine–threonine kinase that belongs to the protein kinase family [38]. AKT and SGK1 are known to act as upstream regulators of GSK3B by activating and phosphorylating it [39]. Phosphorylated GSK3B regulates numerous cellular processes. SGK1, another key downstream molecule in the PI3K/AKT signaling pathway, is a protein kinase belonging to the AGC family that regulates cyclins, apoptosis suppressor proteins and cell metabolism, cyclins, apoptosis suppressor proteins, and cell metabolism [40]. In our study, we examined the colocalization and fluorescence of NeuN and Iba1 with GSK3B, SGK1, p‐GSK3B, and p‐SGK1 in each study group on the day after ICH. Numerous studies have explored the roles of GSK3B and SGK1 in ICH, and the identification of NTF3, PI3K/AKT, and their downstream effector molecules can provide insights for further research on potential therapeutic targets or combination therapies for ICH (Figure S4B–E).

ESK has recently emerged as a novel antidepressant. Approximately half of the patients in the ESK group achieved remission, and two‐thirds responded to treatment by Week 32, underscoring the significance of ESK in treating treatment‐resistant depression [41]. Clinical reports have demonstrated the analgesic effects of ESK for pain relief following scoliosis correction [42], analgesia and sedation during cesarean section [43], and perioperative acute postoperative pain management [3]. Depression and pain are common complications associated with ICH. In addition to causing hemiplegia or other motor impairments, ICH may lead to symptoms of depression or pain, imposing a significant burden on patients' quality of life [44, 45]. In this study, ESK improved mouse neurological function and mobility after ICH and exerted a neuroprotective effect. Thus, the potential use of ESK for treating ICH‐related pain or depression is a promising direction for future research, potentially broadening its application to a larger patient population with secondary conditions following ICH [46].

Our study was limited to adult male mice; therefore, potential differences in the effects of ICH and ESK based on age and sex were not explored. Additionally, our findings showed that a dose of 40 mg/kg did not affect neurological function, liver or kidney pathology, or behavior in mice. ESK at doses of at least 20 mg/kg did not cause any toxic side effects in mice, as indicated by routine blood tests and biochemical assessments of liver and kidney function. However, several side effects of ESK overdose have been reported in clinical practice, including dissociation, dizziness, nausea, sedation, vertigo, sensory delay, somnolence, and feelings of intoxication [47, 48]. ESK may also elevate blood pressure within a certain range. Studies have shown that low‐dose ESK can alleviate hypoxia and hypotension induced by propofol anesthesia in patients and stabilize hemodynamics [49]. Therefore, careful monitoring of patients is necessary to ensure medication safety in future clinical applications. As this study was limited to animal models, further clinical research is required to validate its therapeutic efficacy.

## 5 Conclusions

We confirm that ESK exerts neuroprotective effects following ICH. These effects may be associated with neural cell regeneration and axonal repair, with the NTF3/PI3K/AKT pathway playing a crucial role in this process. Therefore, ESK may have potential clinical applications in treating ICH and other neurodegenerative diseases.

## Author Contributions

Changsheng Li and Jian Wang designed and conceived the study. Xiaoyu Niu, Changsheng Li, and Jian Wang wrote the manuscript. Xiaoyu Niu, Yuanyuan Zheng, Wang Wang, and Shaoshuai Wang performed the experiments and acquired the data. Xiaoyu Niu and Liwei Zhang conducted a bioinformatics analysis. Xiaoyu Niu, Yuanyuan Zheng, and Gaiqing Yang analyzed the data and edited the figures. Xihua Lu, Ting Zhao, Nan Li, Junyang Wang, Qiang Li, Junmin Wang, and Jian Wang drafted, edited, and revised the manuscript. All authors approved the final article.

## Conflicts of Interest

The authors declare no conflicts of interest.

## Supporting information


Data S1.

Table S1.

Table S2.

Table S3.

Table S4.

Table S5.

## Data Availability

The data that support the findings of this study are available from the corresponding author upon reasonable request.
