# Supplementary material for: Esketamine Provides Neuroprotection After Intracerebral Hemorrhage in Mice via the NTF3/PI3K/AKT Pathway
Source: CNS Neurosci Ther. 2024 Dec 17;30(12):e70145. doi: 10.1111/cns.70145 (PMC11652676; doi:10.1111/cns.70145)

Part Ⅰ. Raw Western blot bands in Figure 2C
1: Sham
2: ICH+Vehicle
3: ICH+ESK20

A. NeuN

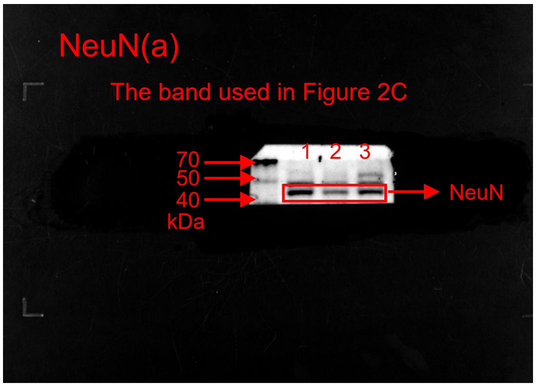

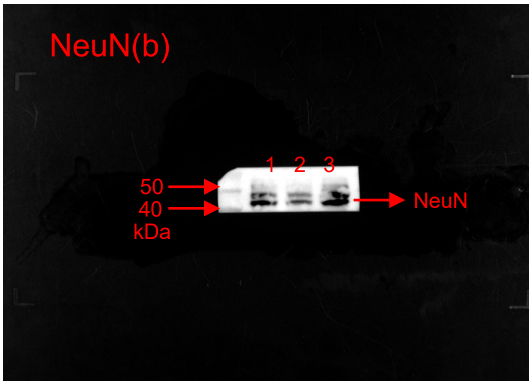

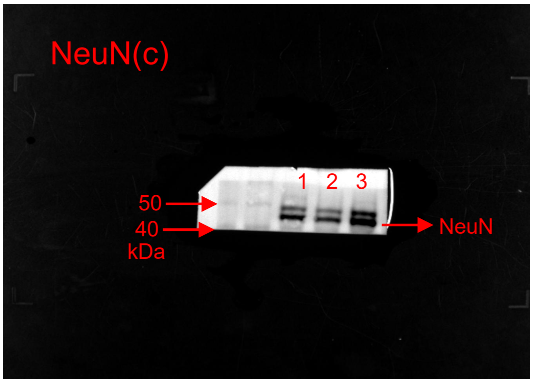

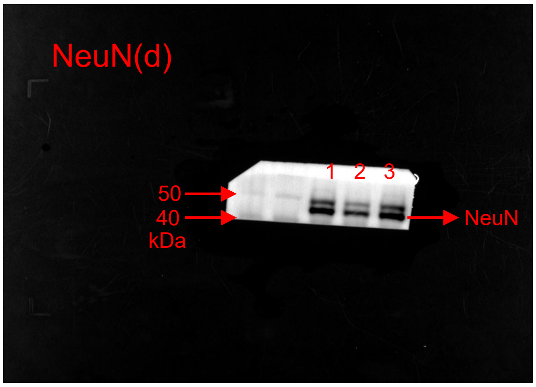

B. GFAP

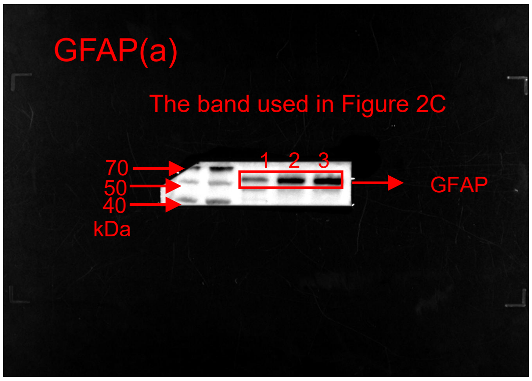

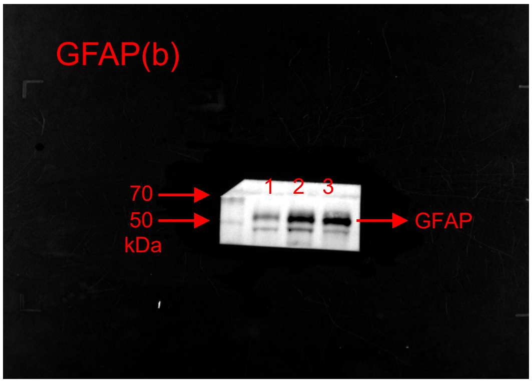

B. GFAP

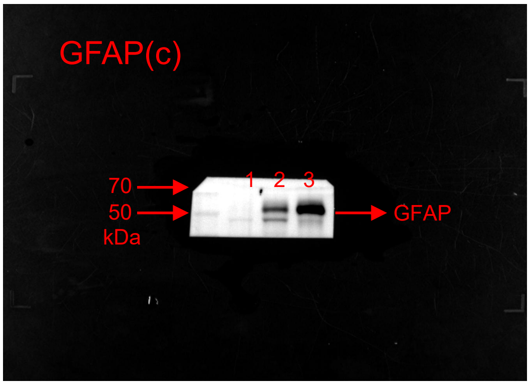

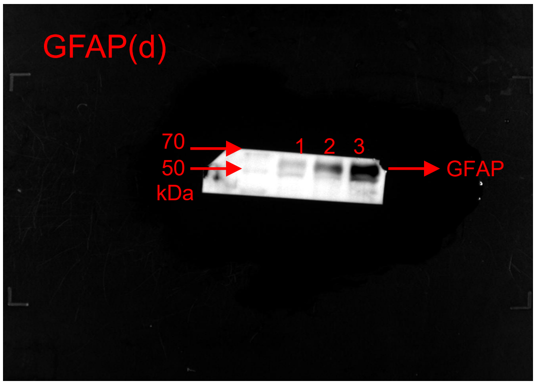

C. NSE

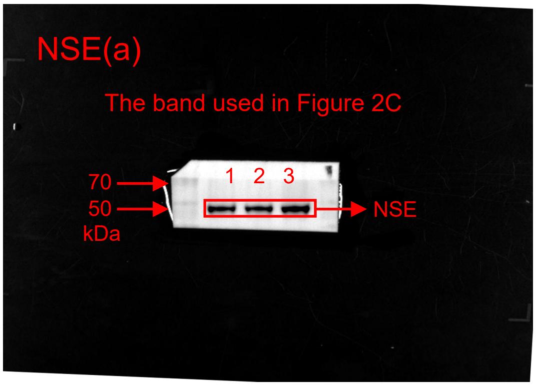

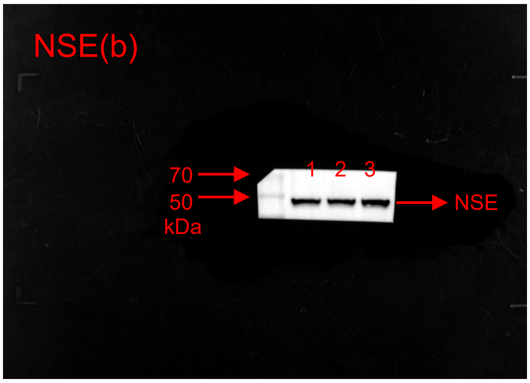

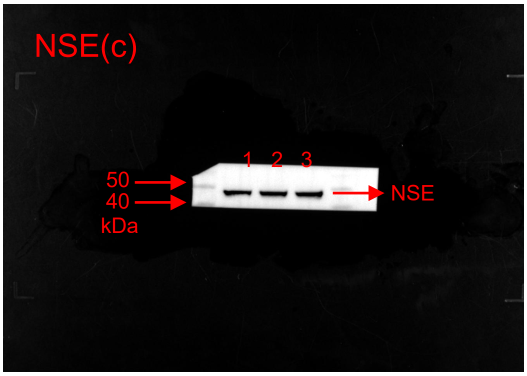

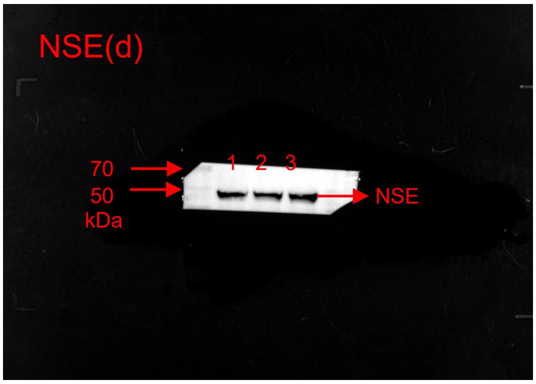

D. MBP

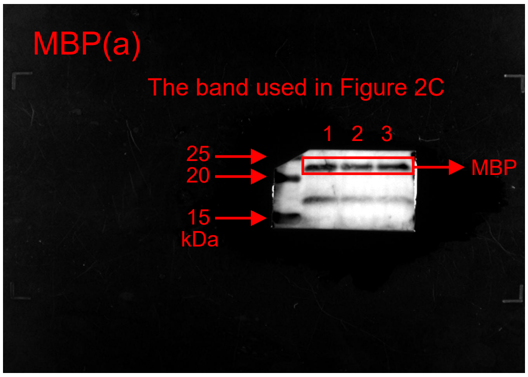

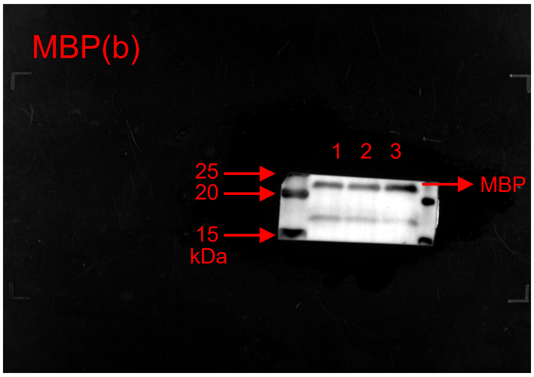

D. MBP

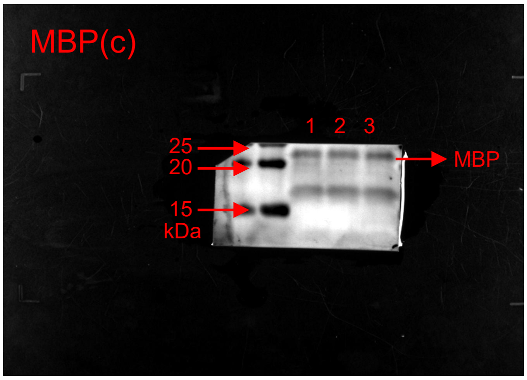
MBP(c)
1 2 3
25
20
15
kDa
MBP

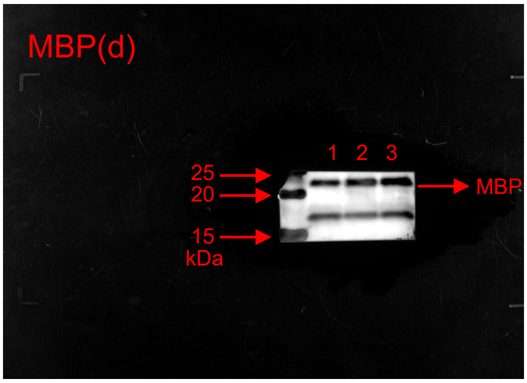
MBP(d)
1 2 3
25
20
15
kDa
MBP

E. GAPDH

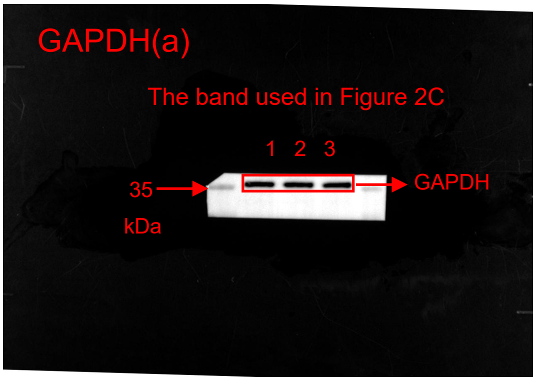
GAPDH(a)
The band used in Figure 2C
1 2 3
35
kDa
GAPDH

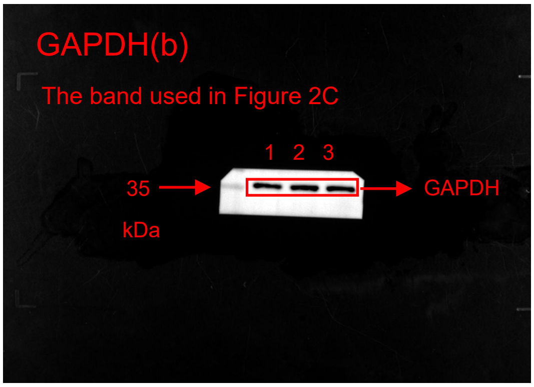
GAPDH(b)
The band used in Figure 2C
1 2 3
35
kDa
GAPDH

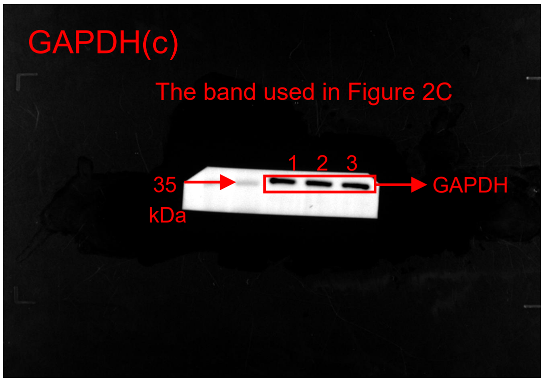
GAPDH(c)
The band used in Figure 2C
1 2 3
35
kDa
GAPDH

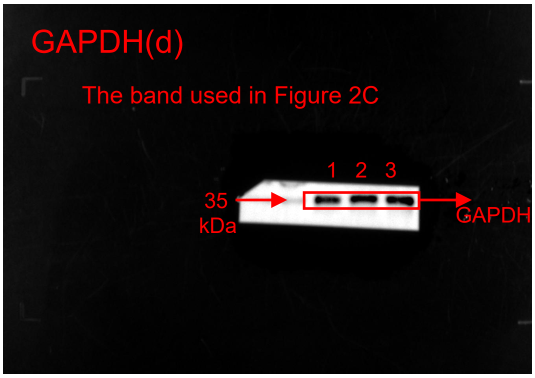
GAPDH(d)
The band used in Figure 2C
1 2 3
35
kDa
GAPDH

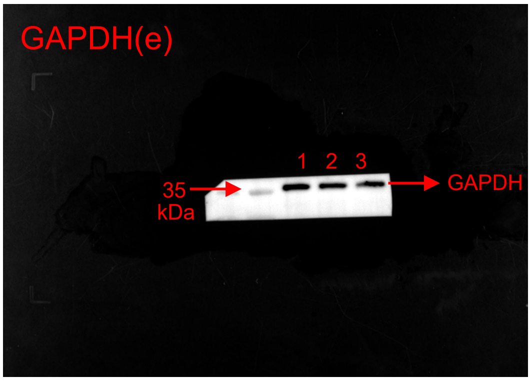
GAPDH(e)
1 2 3
35
kDa
GAPDH

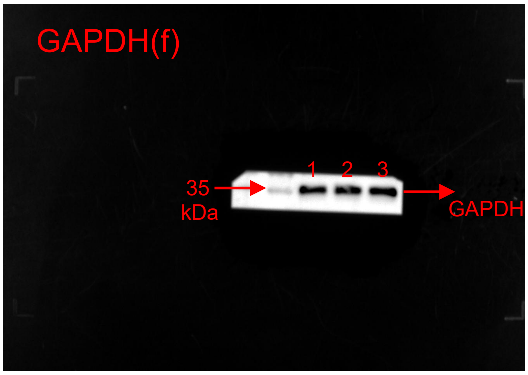
GAPDH(f)
1 2 3
35
kDa
GAPDH

E. GAPDH

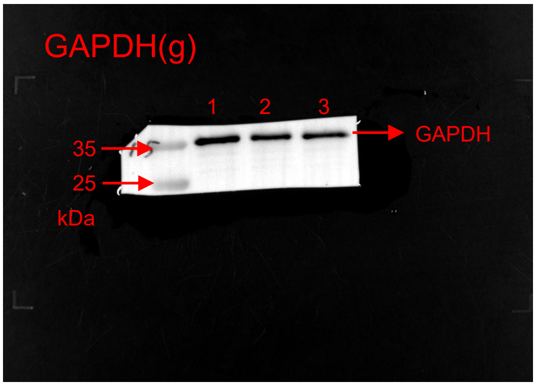

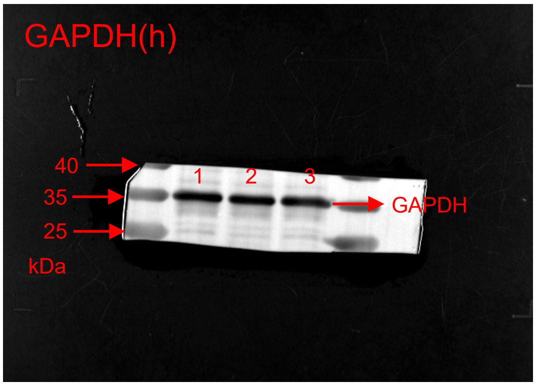

Part Ⅱ. Raw Western blot bands in Figure 5A

1: Sham
2: 6h
3: 12h
4:D1
5:D3
6:D7

A. NTF3

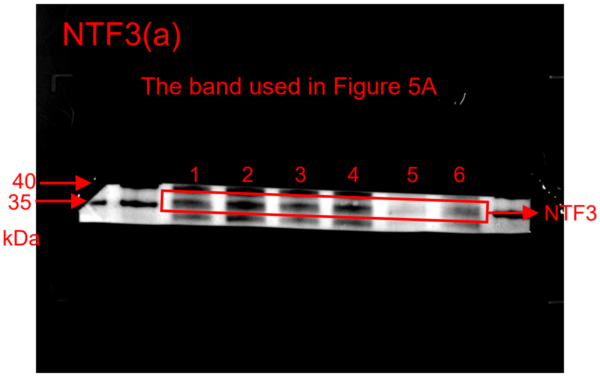

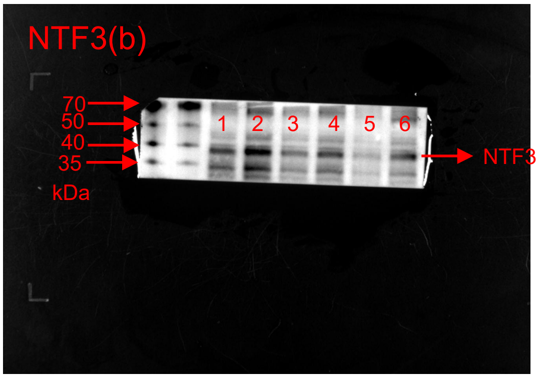

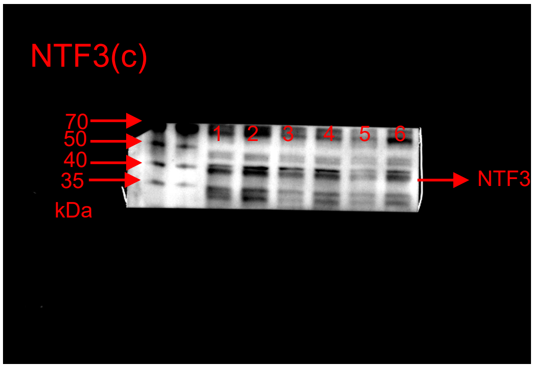

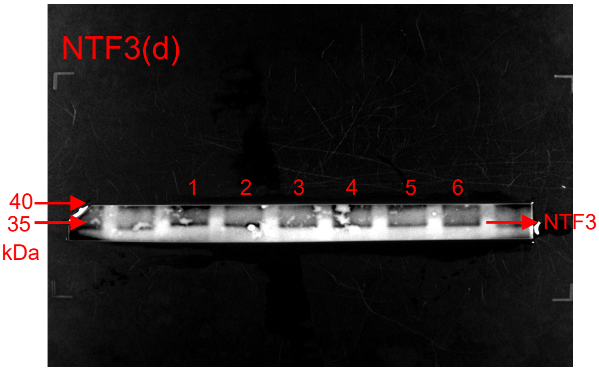

B. Tubulin

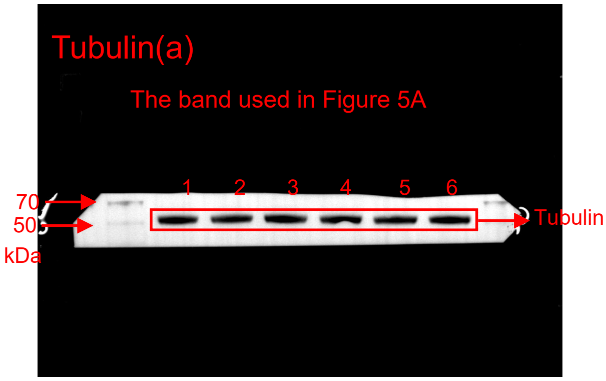

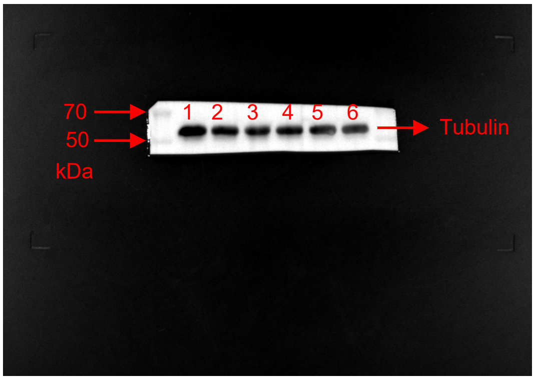

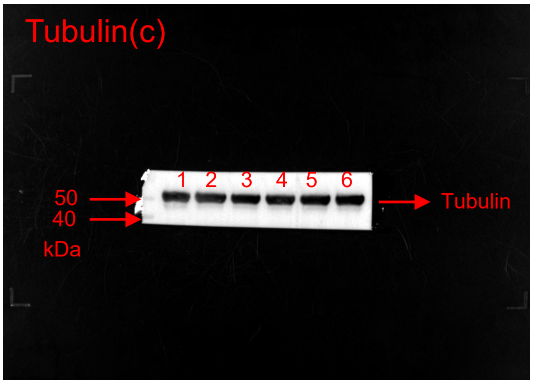

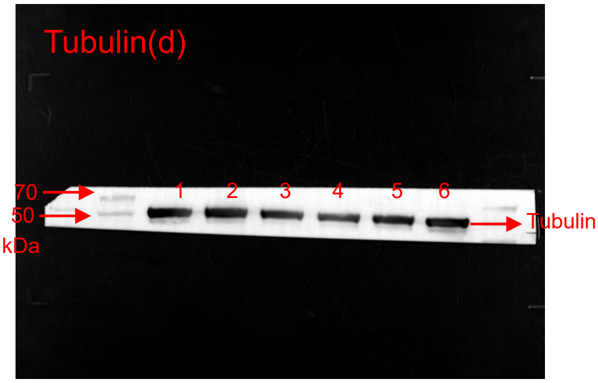

Part Ⅲ. Raw Western blot bands in Figure 5E

1: Sham
2: ICH+Vehicle
3: ICH+ESK20

A. NTF3

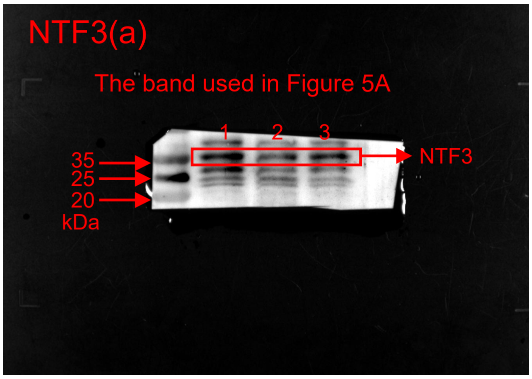

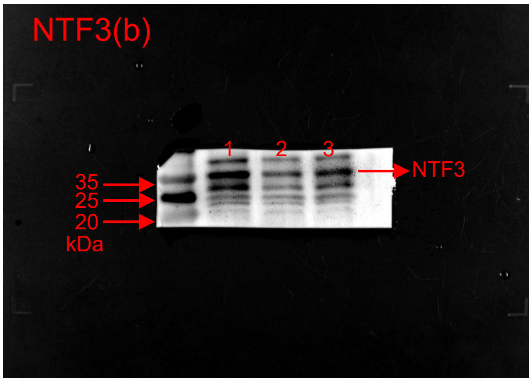

A. NTF3

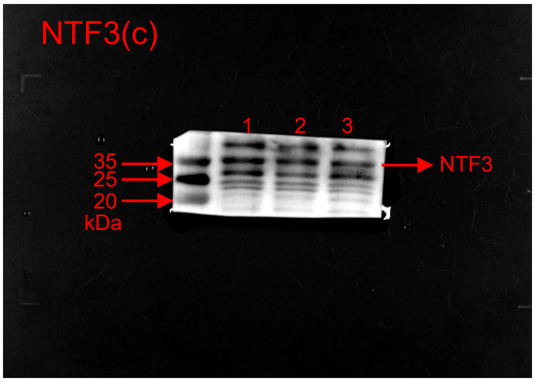

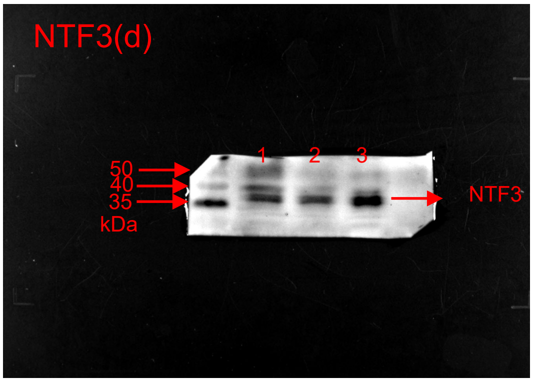

B. BDNF

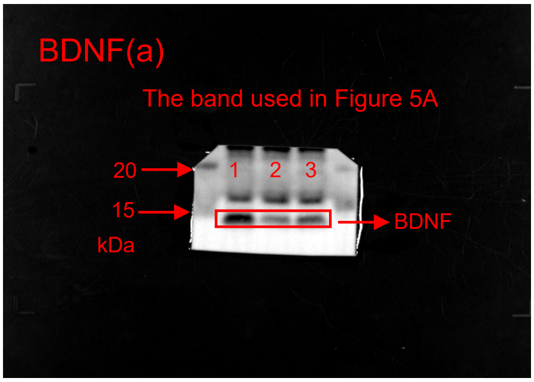

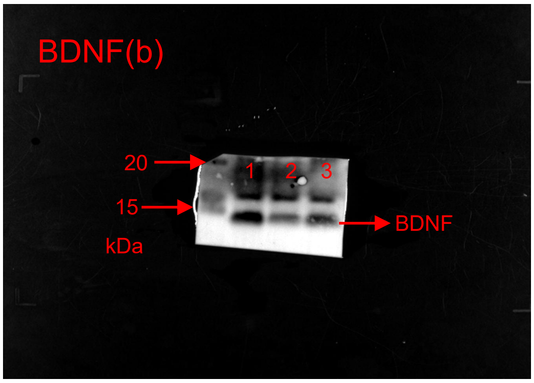

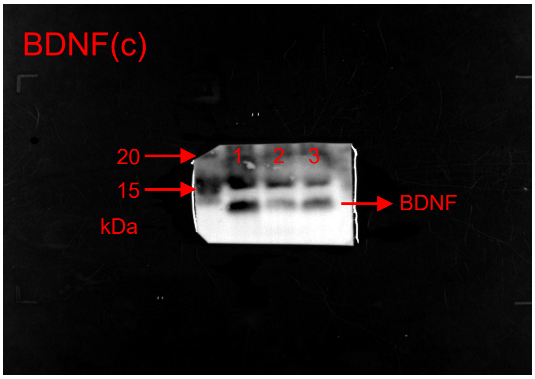

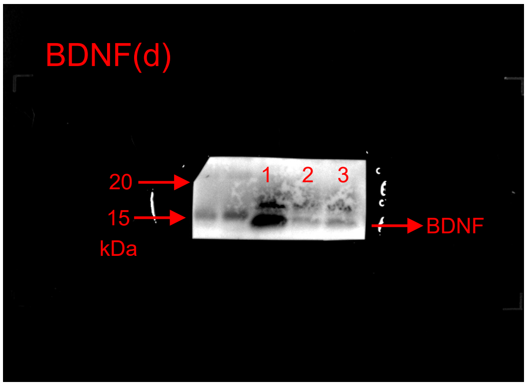

C. p-TrkB

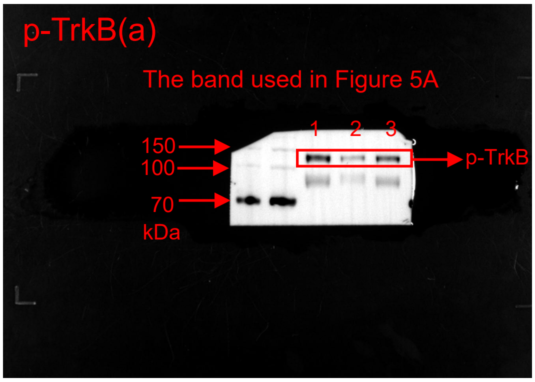

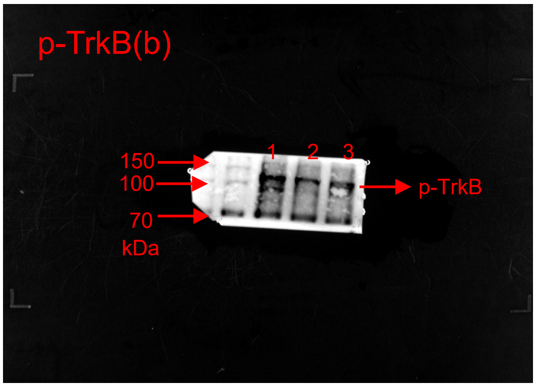

C. p-TrkB

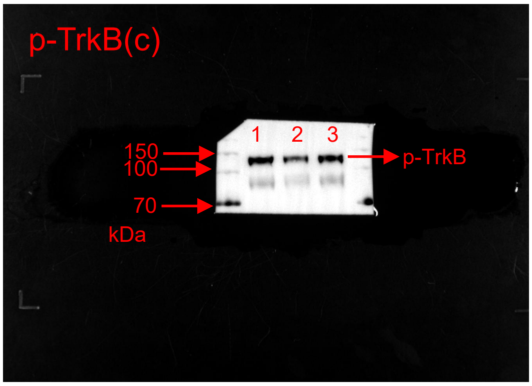

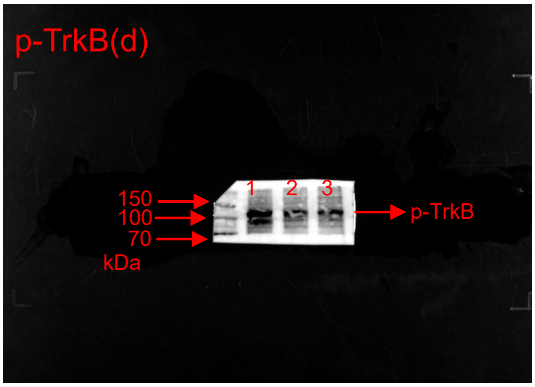

D. TrkB

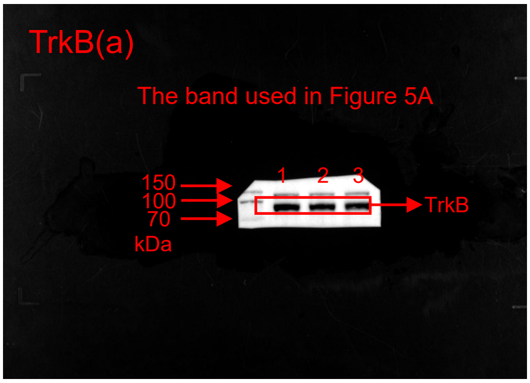

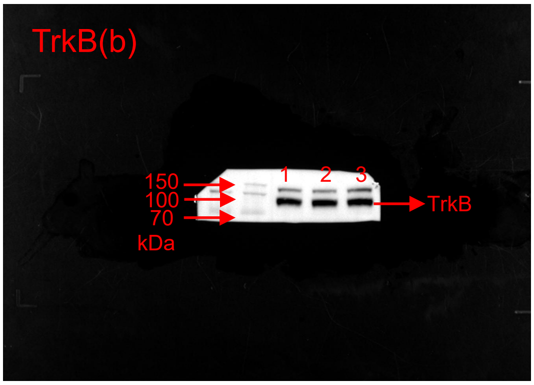

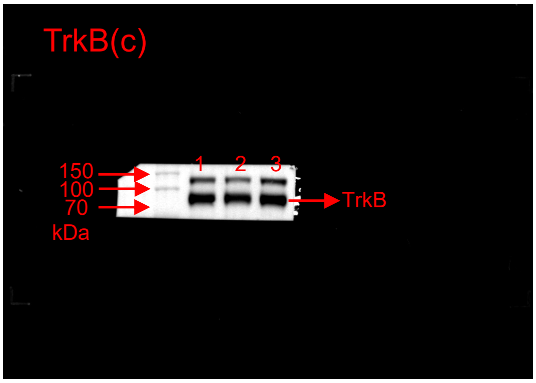

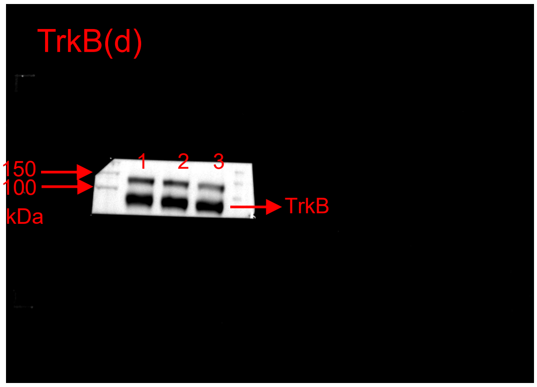

E. p-TrkC

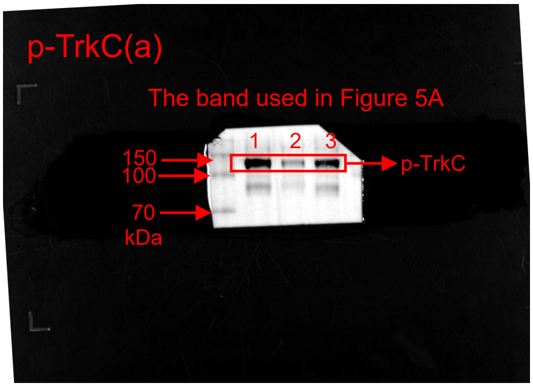

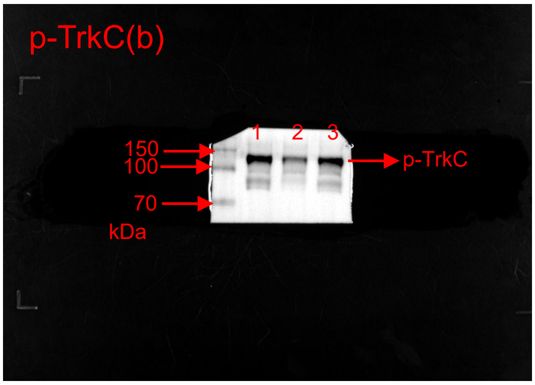

E. p-TrkC

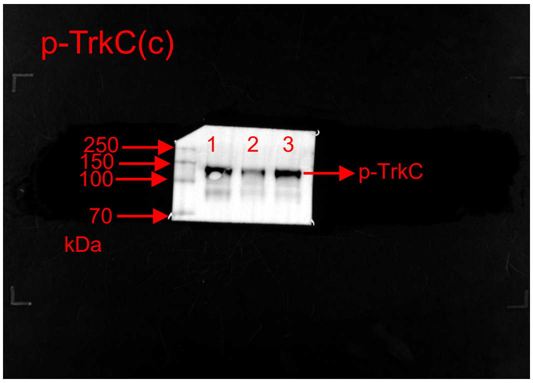

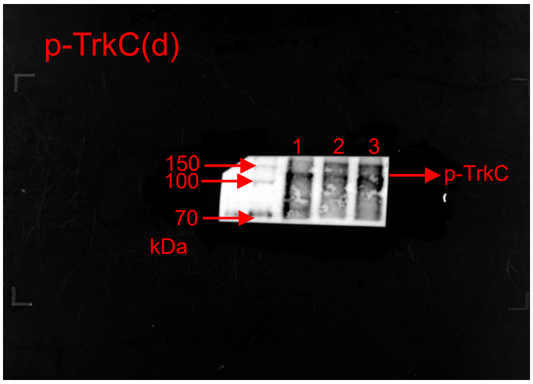

F. TrkC

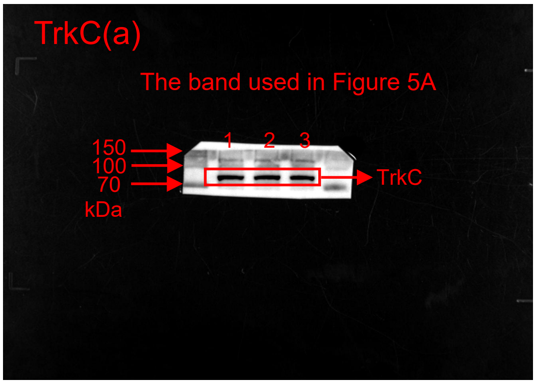

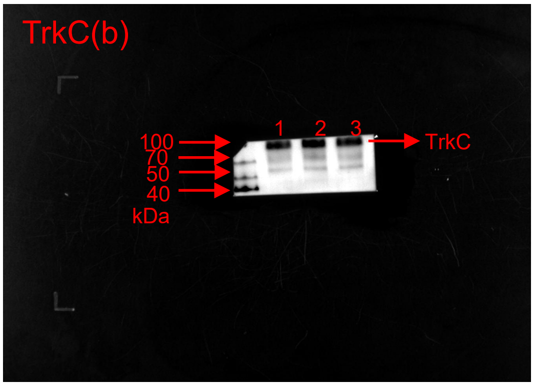

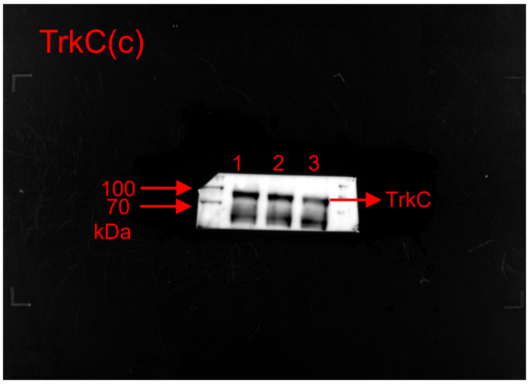

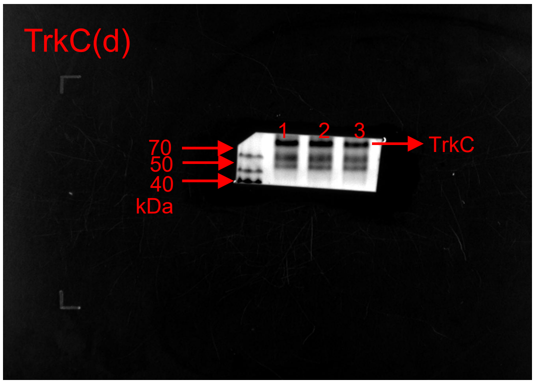

G. Tubulin

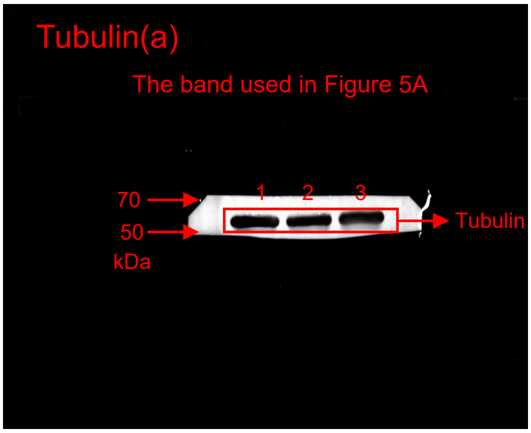

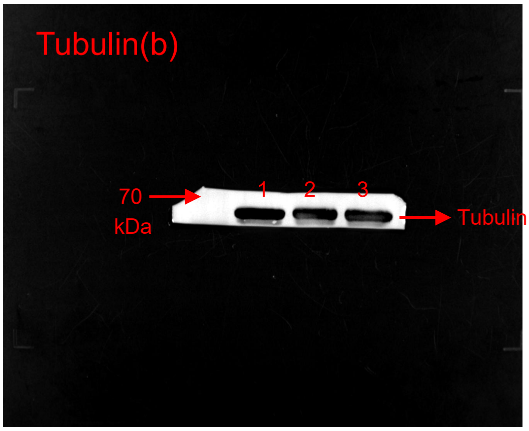

G. Tubulin

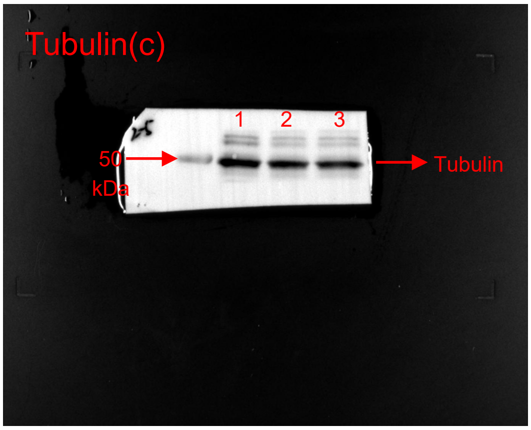

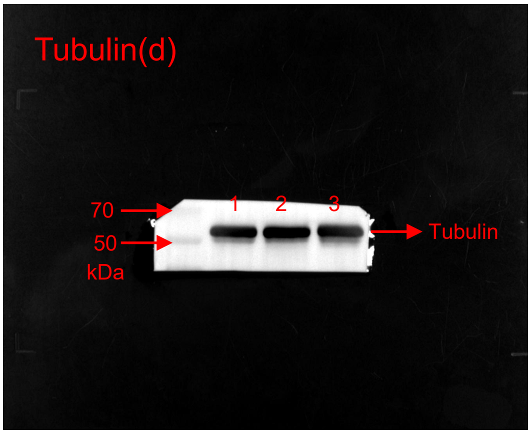

Part Ⅳ. Raw Western blot bands in Figure 6D

1: Sham

2: ICH+Vehicle

3: ICH+ESK20

4: ICH+ESK20+AAV-NC

5: ICH+ESK20+AAV-ShNTF3

A. NTF3

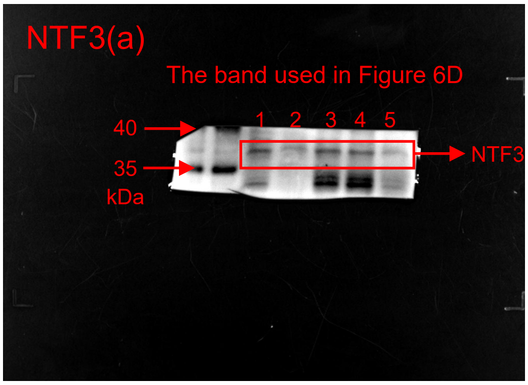

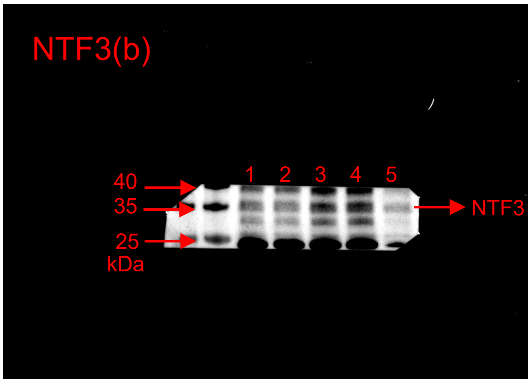

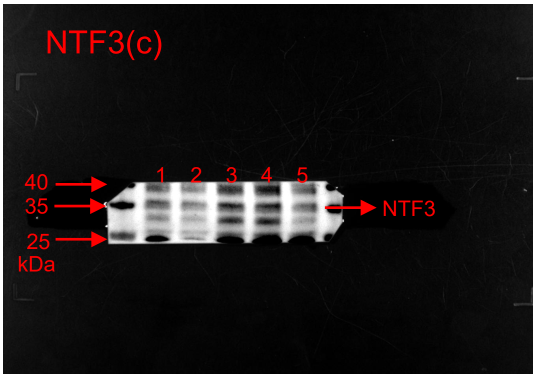

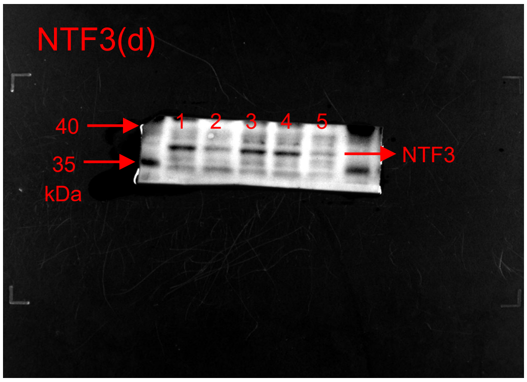

B. PI3K

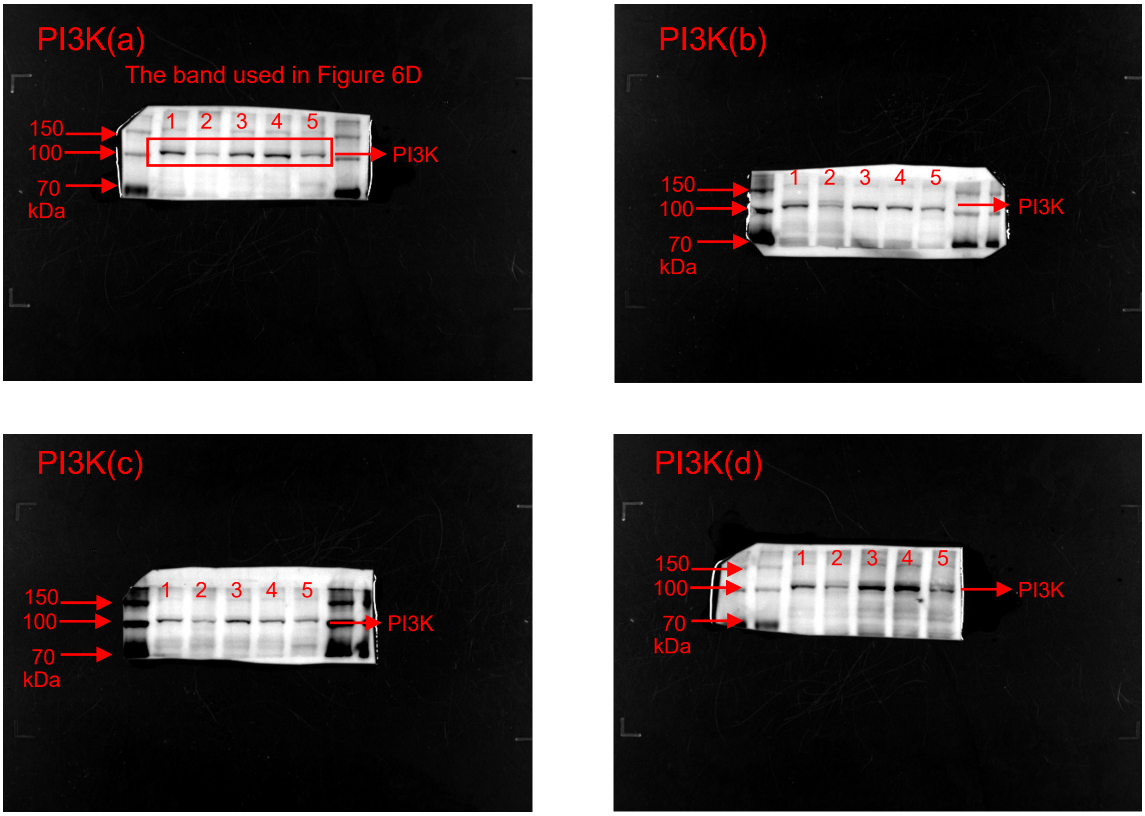

PI3K(a)
The band used in Figure 6D
150
100
70
kDa
1 2 3 4 5
PI3K
PI3K(b)
150
100
70
kDa
1 2 3 4 5
PI3K
PI3K(c)
150
100
70
kDa
1 2 3 4 5
PI3K
PI3K(d)
150
100
70
kDa
1 2 3 4 5
PI3K

C. p-AKT

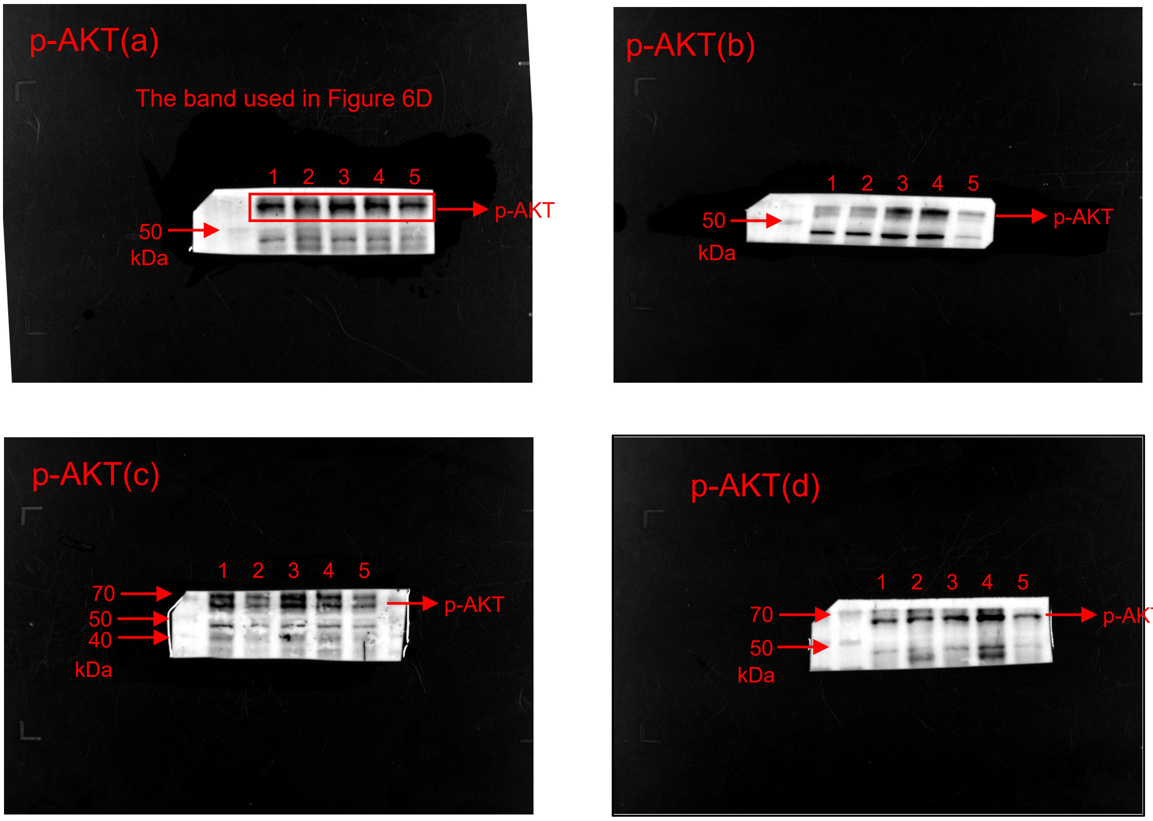

p-AKT(a)
The band used in Figure 6D
1 2 3 4 5
p-AKT
50
kDa
p-AKT(b)
1 2 3 4 5
50
kDa
p-AKT
p-AKT(c)
1 2 3 4 5
70
50
40
kDa
p-AKT
p-AKT(d)
1 2 3 4 5
70
50
kDa
p-AKT

D. AKT

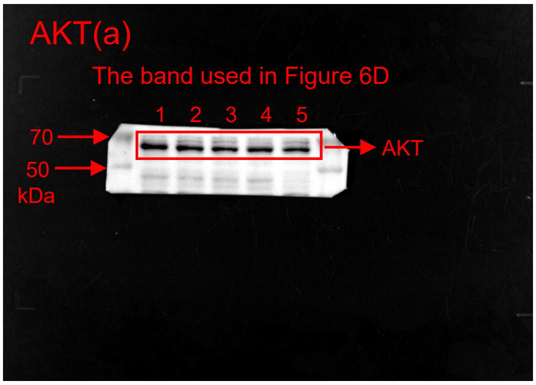

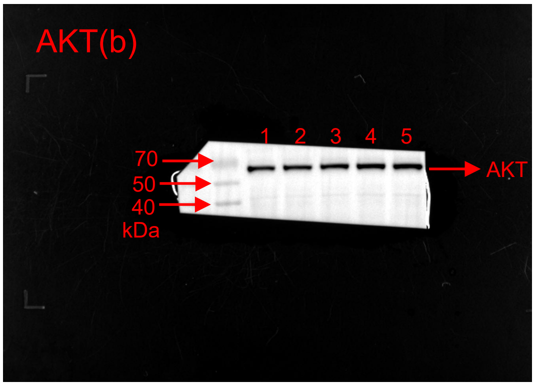

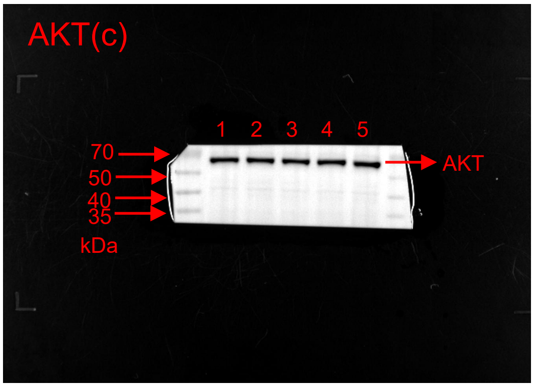

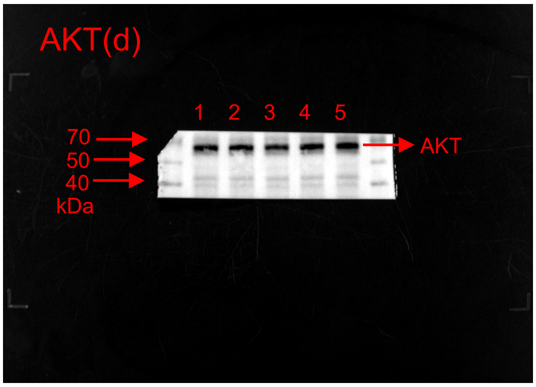

E. p-GSK3B

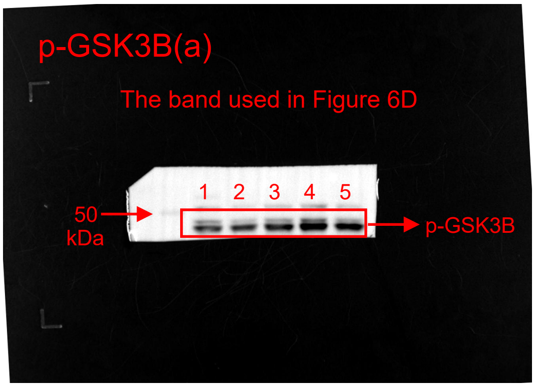

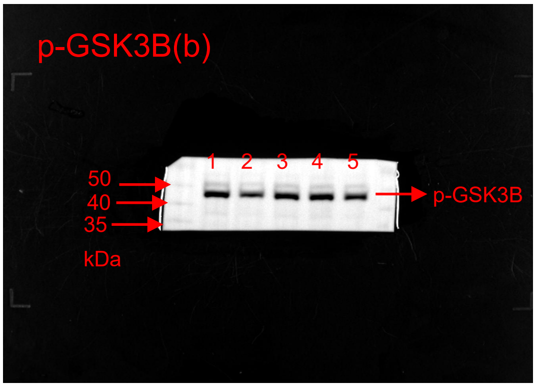

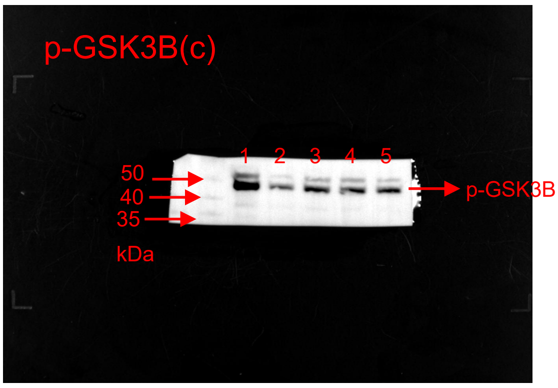

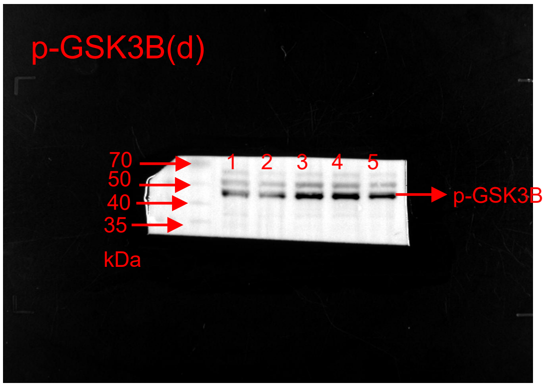

F. GSK3B

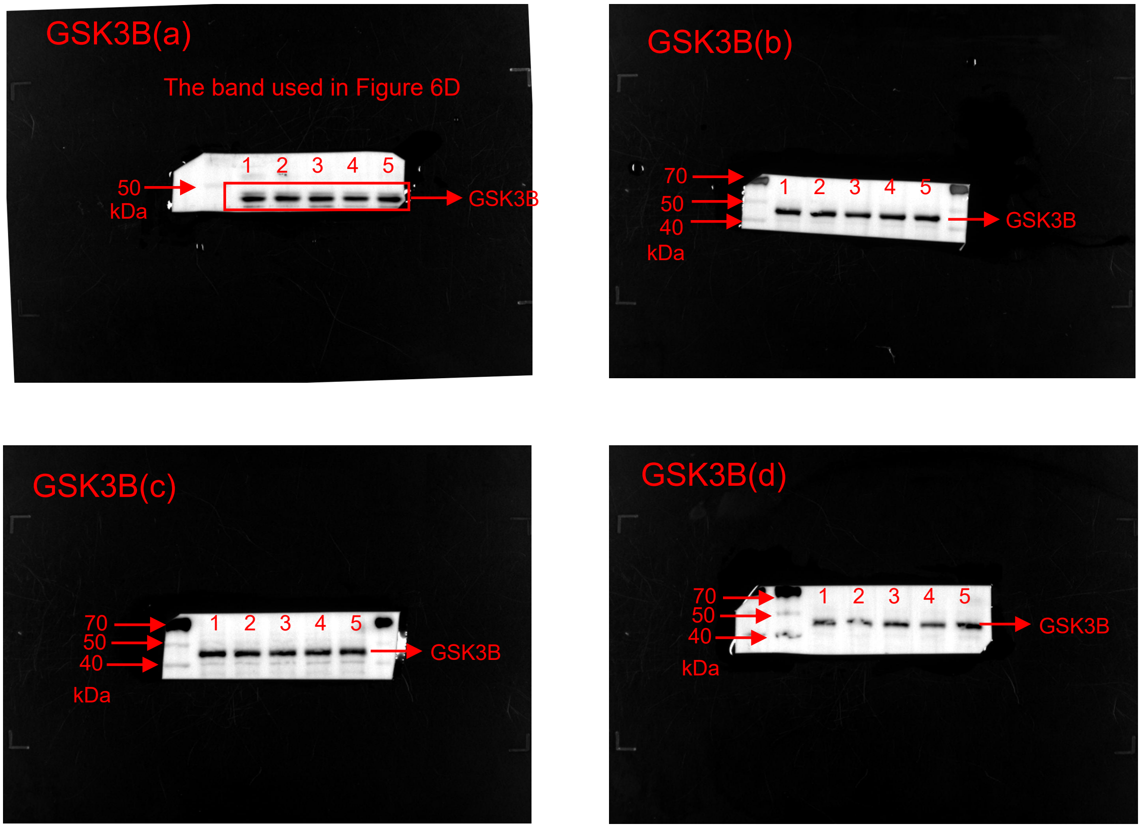

G. p-SGK1

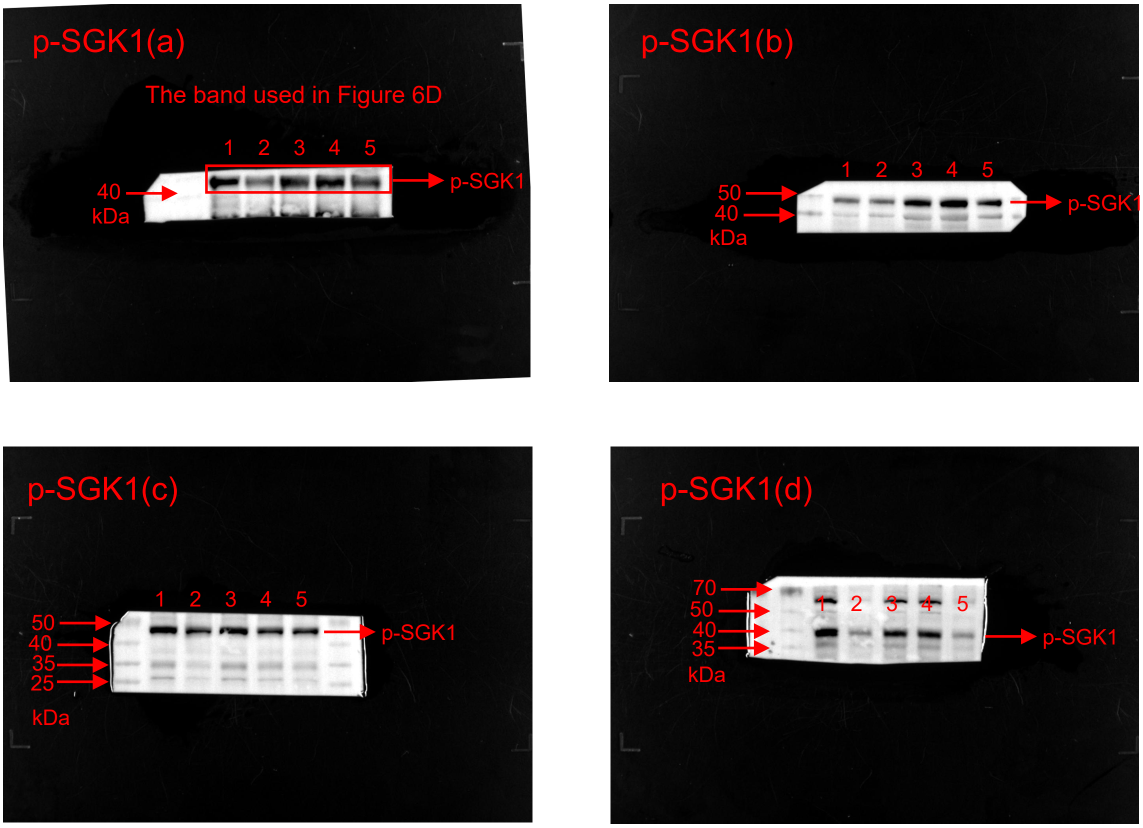

H. SGK1

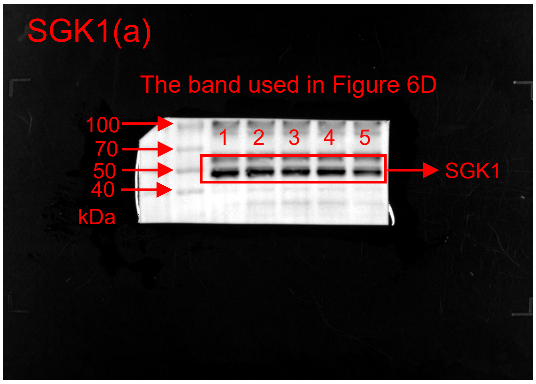

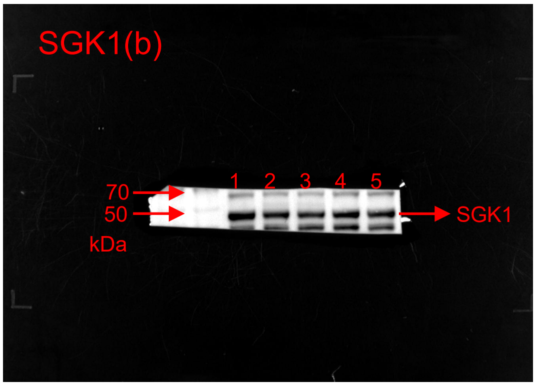

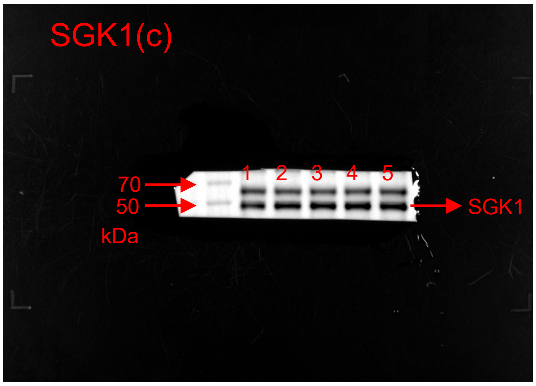

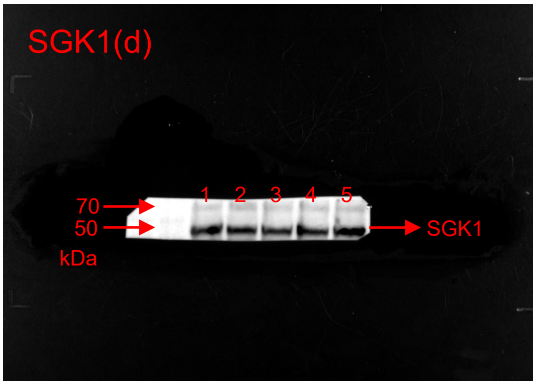

I. Tubulin

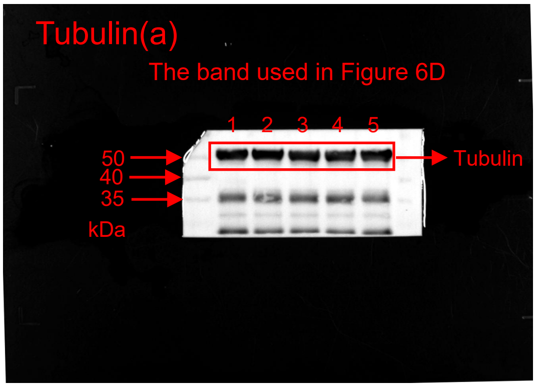

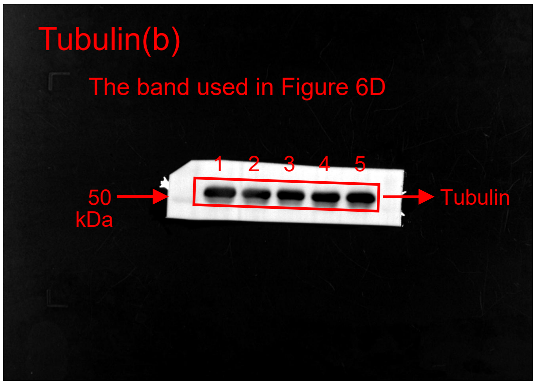

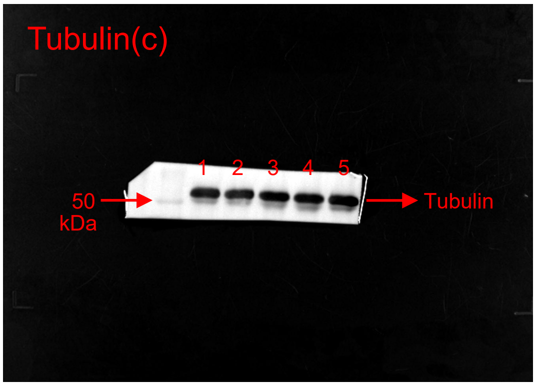

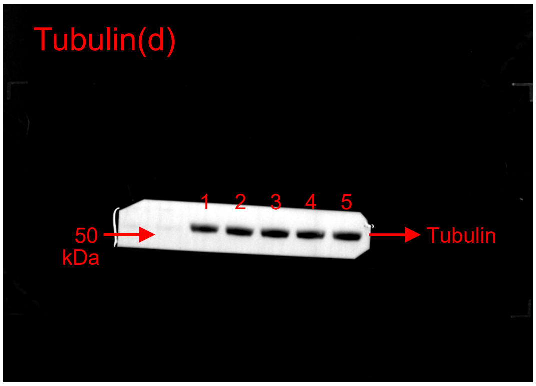

I. Tubulin

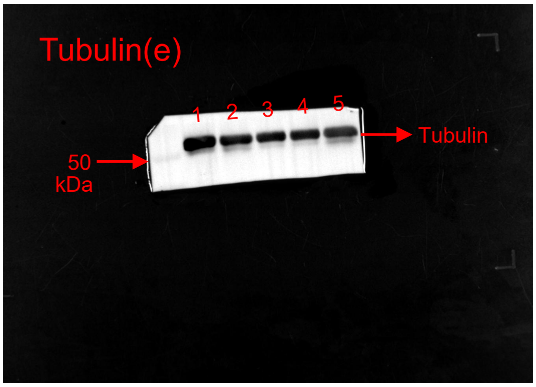

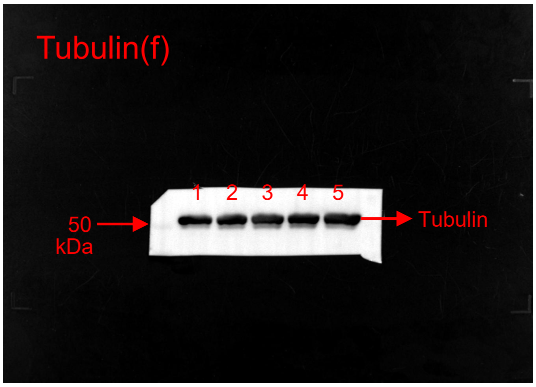

Part Ⅴ. Raw Western blot bands in Figure 7D

1: Sham
2: ICH+Vehicle
3: ICH+ESK20
4: ICH+ESK20+DMSO
5: ICH+ESK20+LY294002

A. NTF3

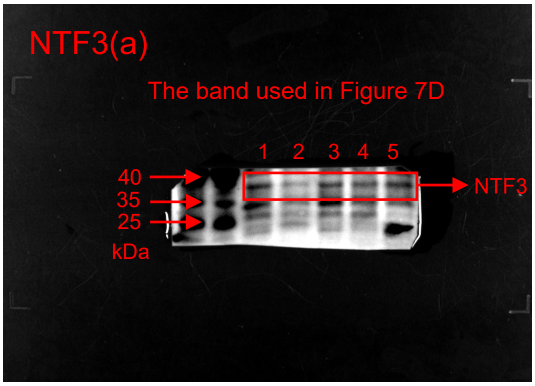

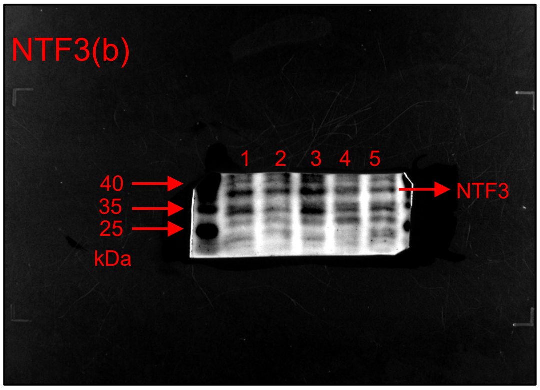

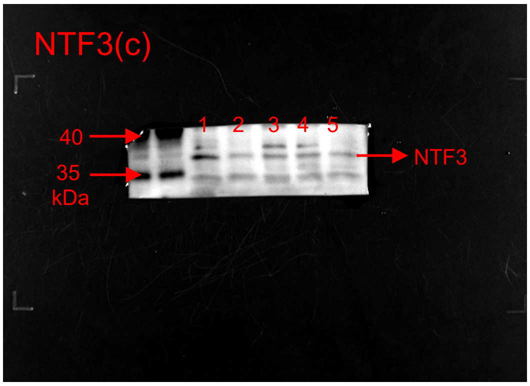

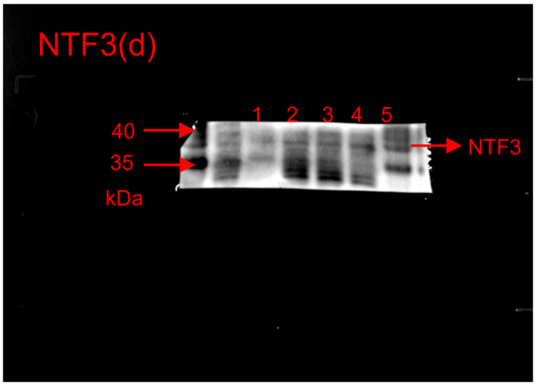

B. PI3K

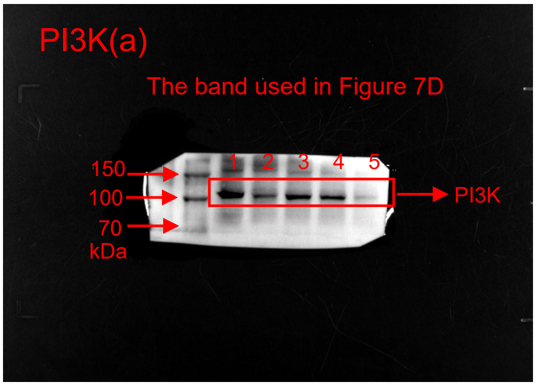
PI3K(a)
The band used in Figure 7D
150
100
70
kDa
1 2 3 4 5
PI3K

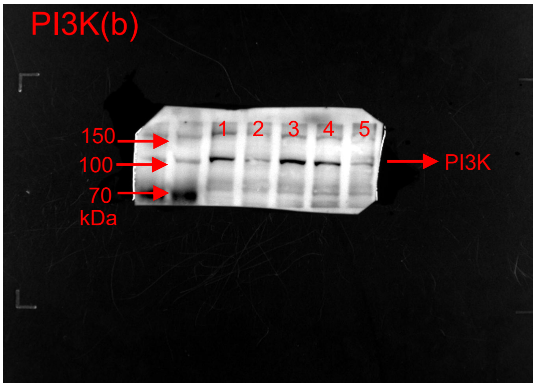
PI3K(b)
150
100
70
kDa
1 2 3 4 5
PI3K

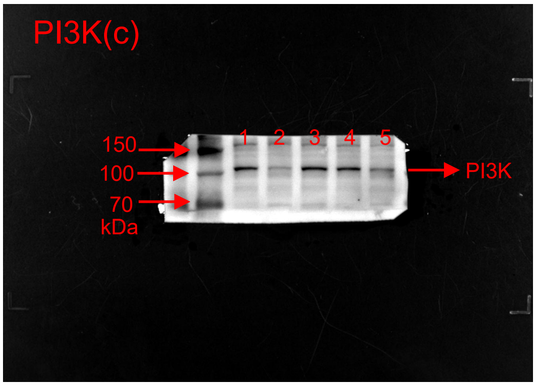
PI3K(c)
150
100
70
kDa
1 2 3 4 5
PI3K

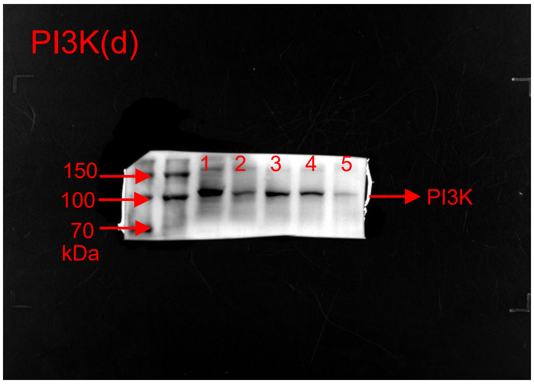
PI3K(d)
150
100
70
kDa
1 2 3 4 5
PI3K

C. p-AKT

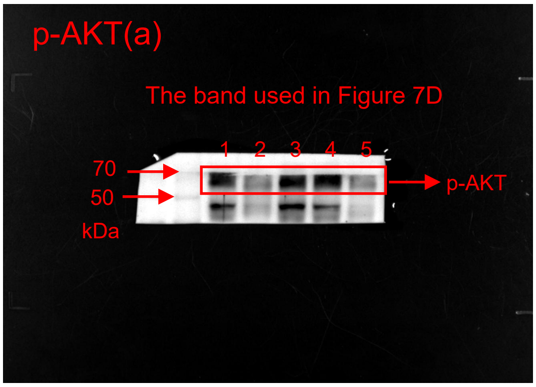
p-AKT(a)
The band used in Figure 7D
70
50
kDa
1 2 3 4 5
p-AKT

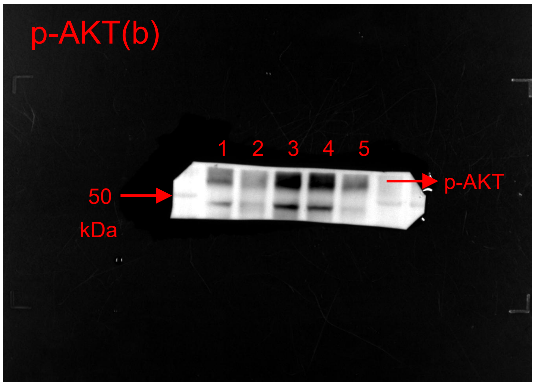
p-AKT(b)
50
kDa
1 2 3 4 5
p-AKT

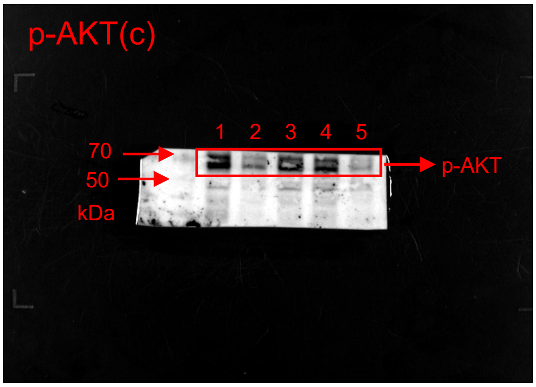
p-AKT(c)
70
50
kDa
1 2 3 4 5
p-AKT

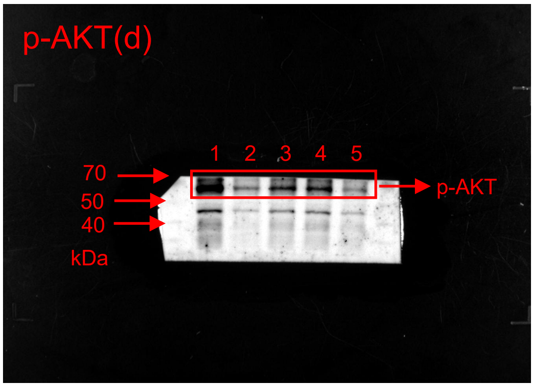
p-AKT(d)
70
50
40
kDa
1 2 3 4 5
p-AKT

D. AKT

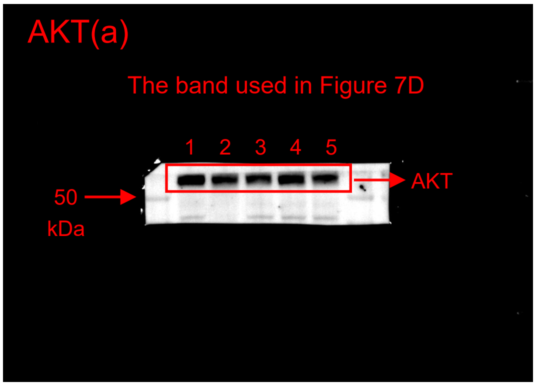

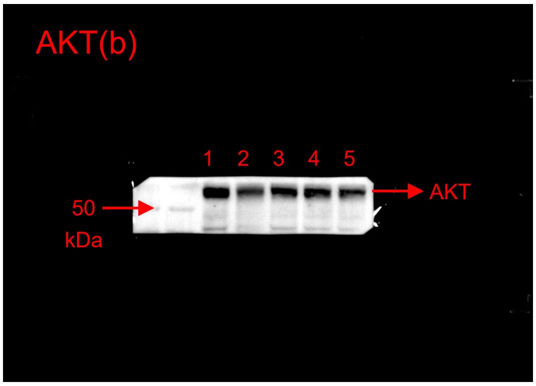

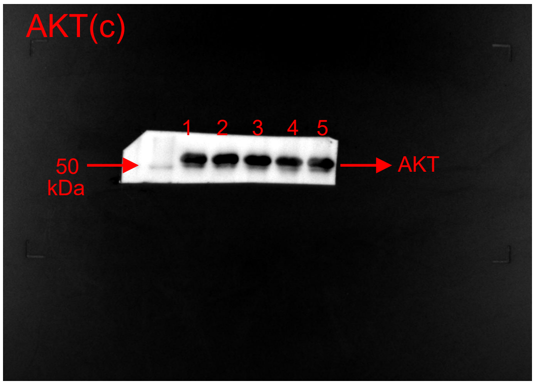

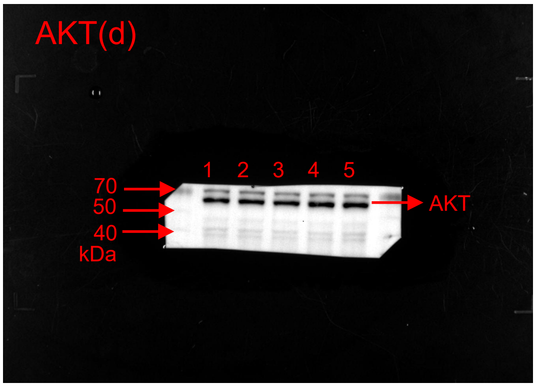

E. p-GSK3B

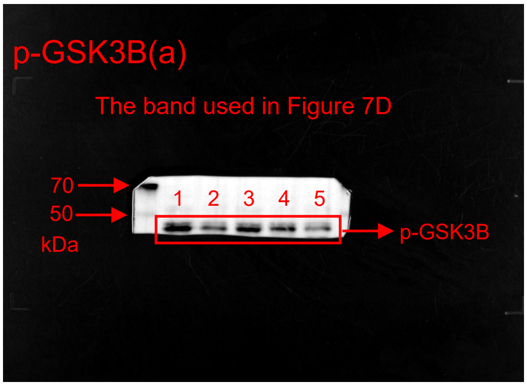

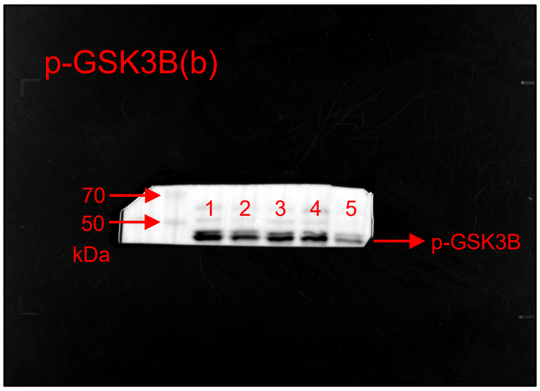

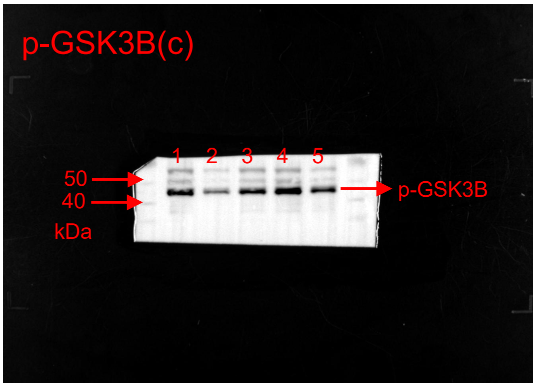

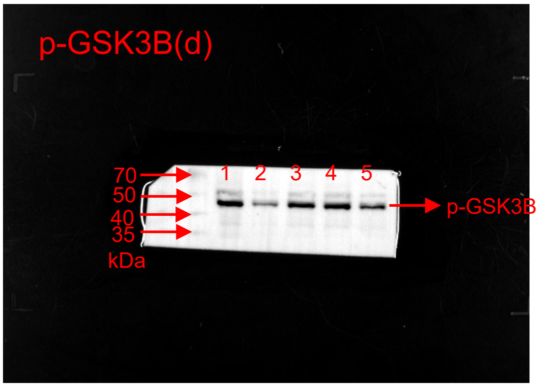

F. GSK3B

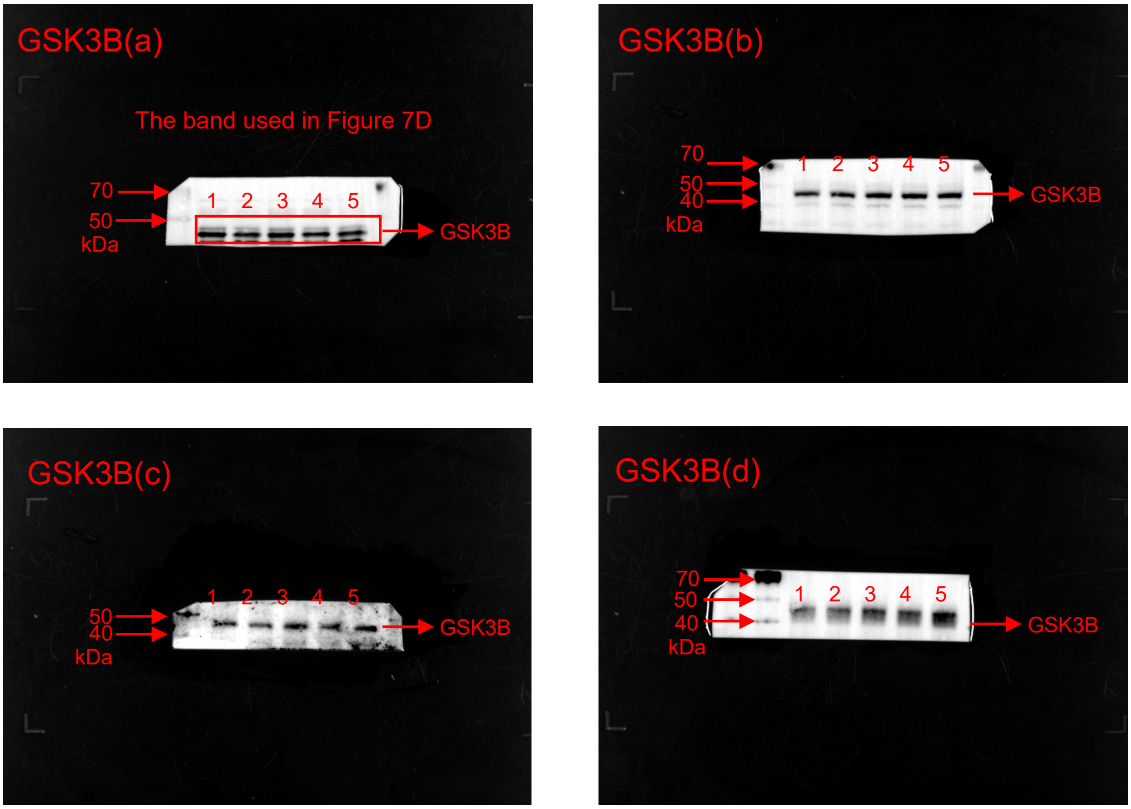

G. p-SGK1

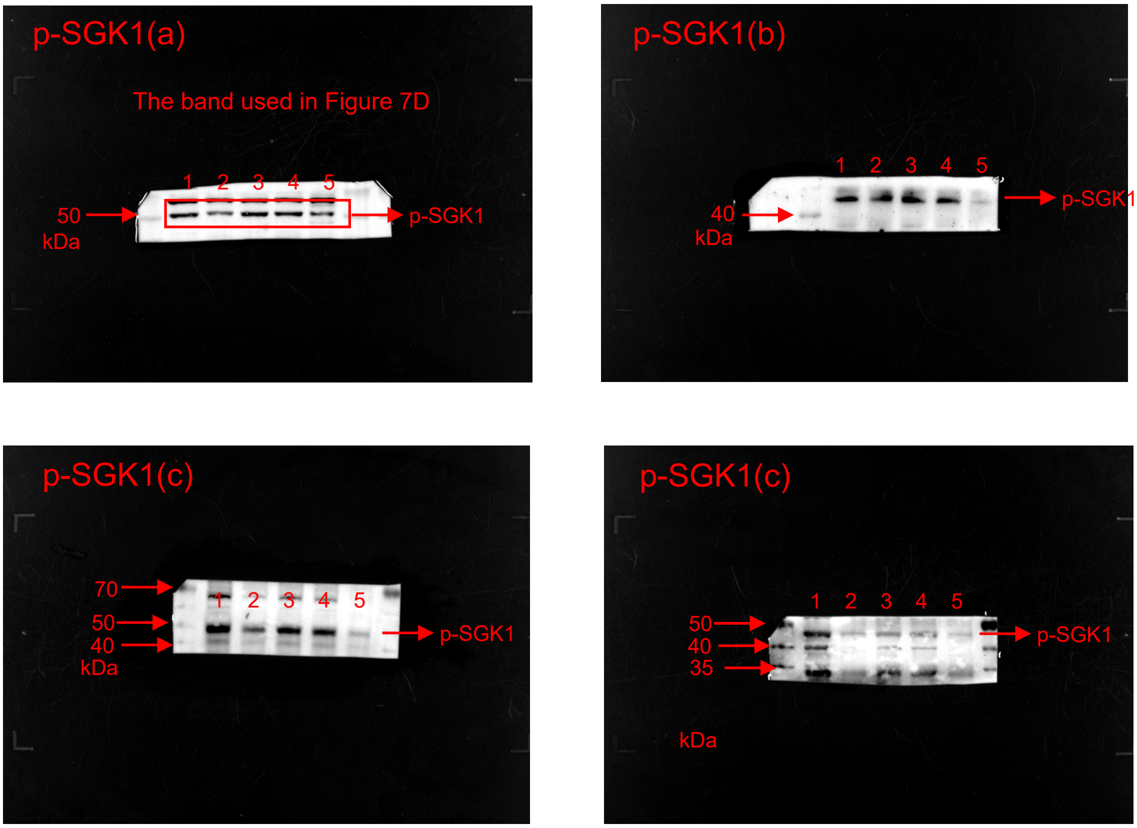

H. SGK1

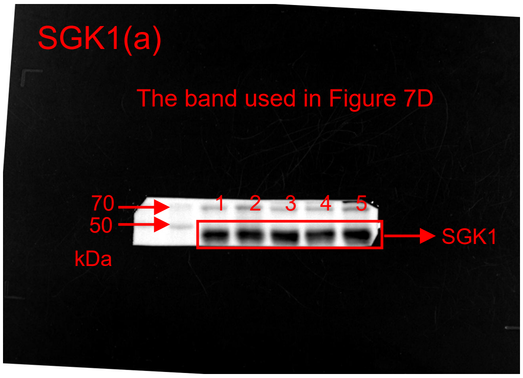

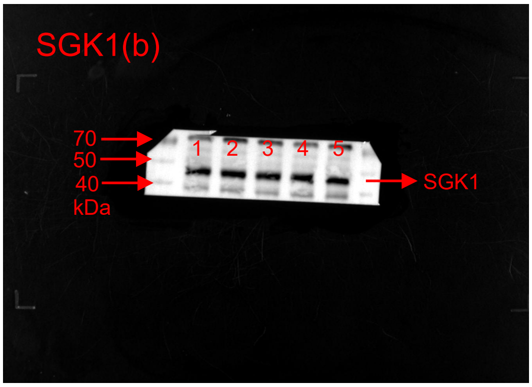

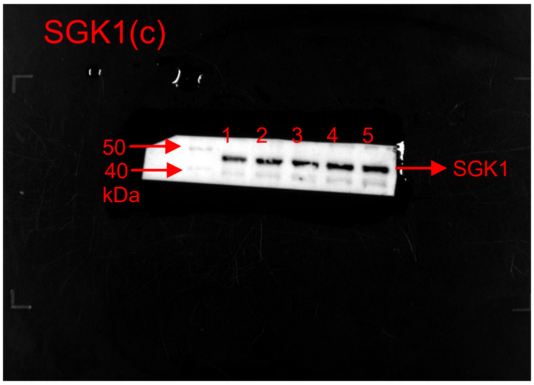

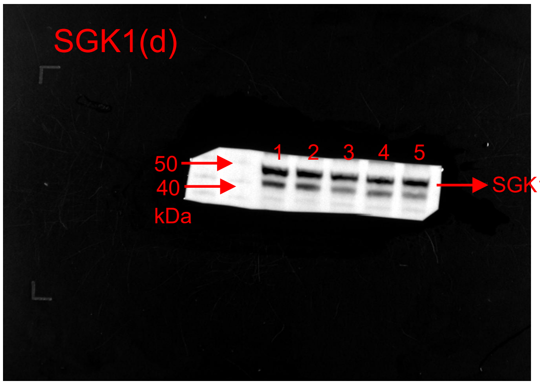

I. Tubulin

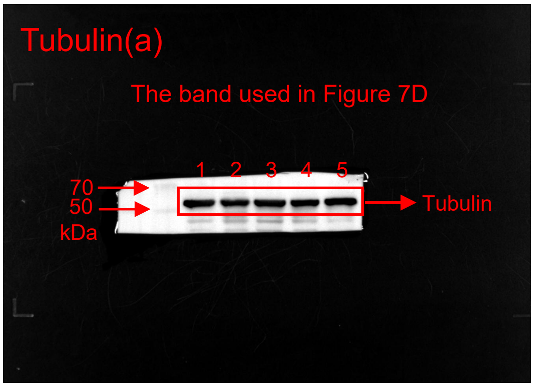

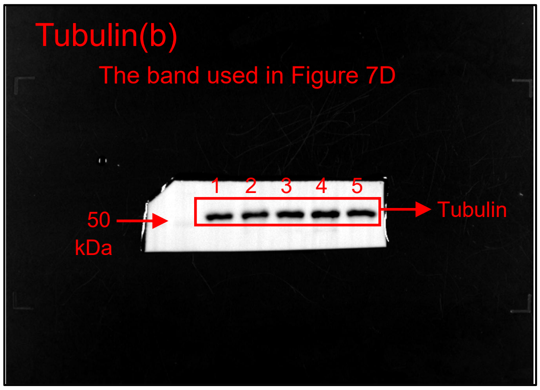

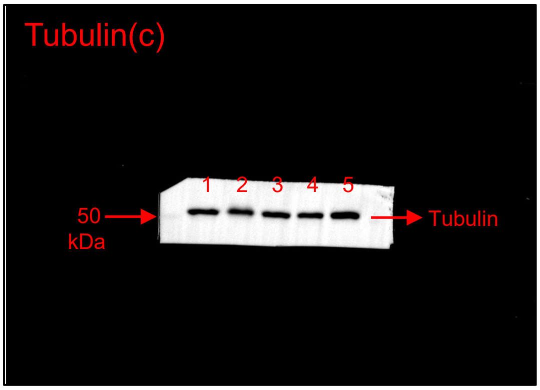

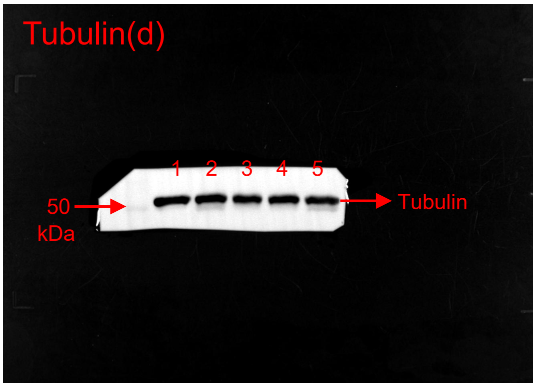

I. Tubulin

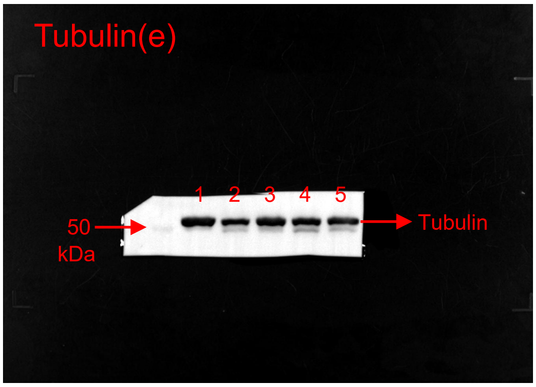

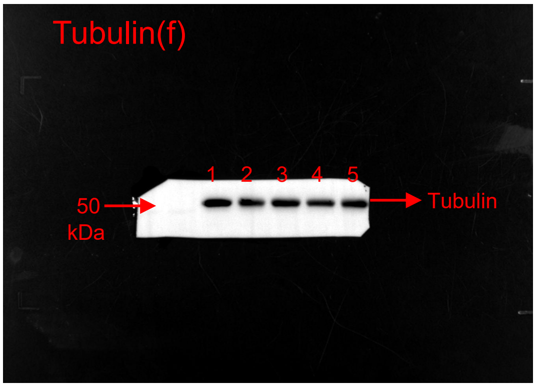

Part Ⅵ. Raw Western blot bands in Supplementary Figure 3A

1: Sham
2: 6h
3: 12h
4:D1
5:D3
6:D7

A. BDNF

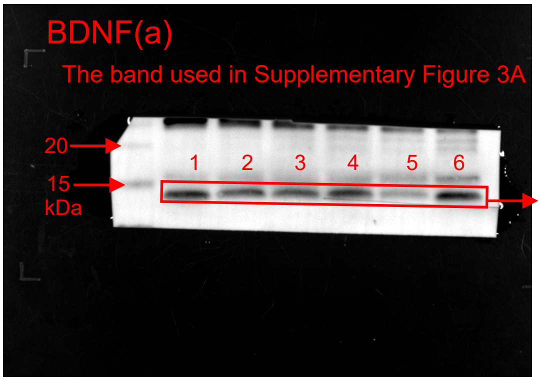

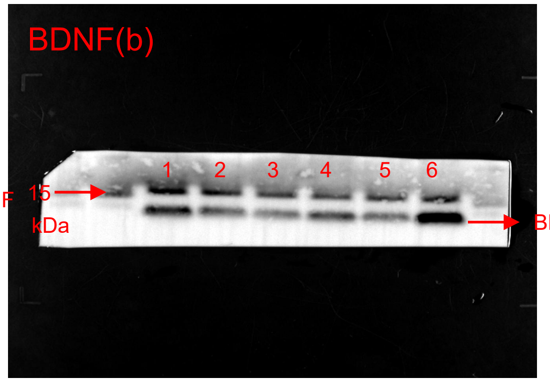

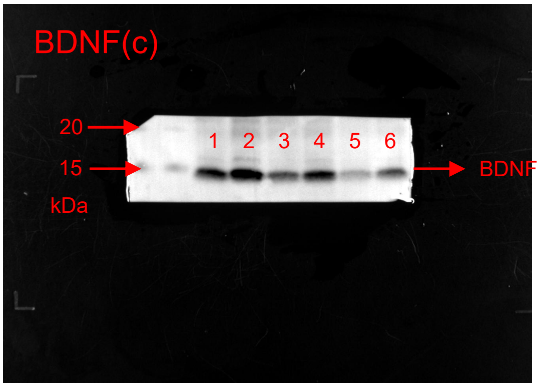

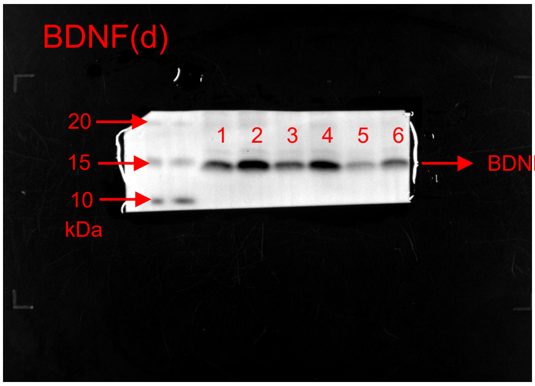

B. PI3K

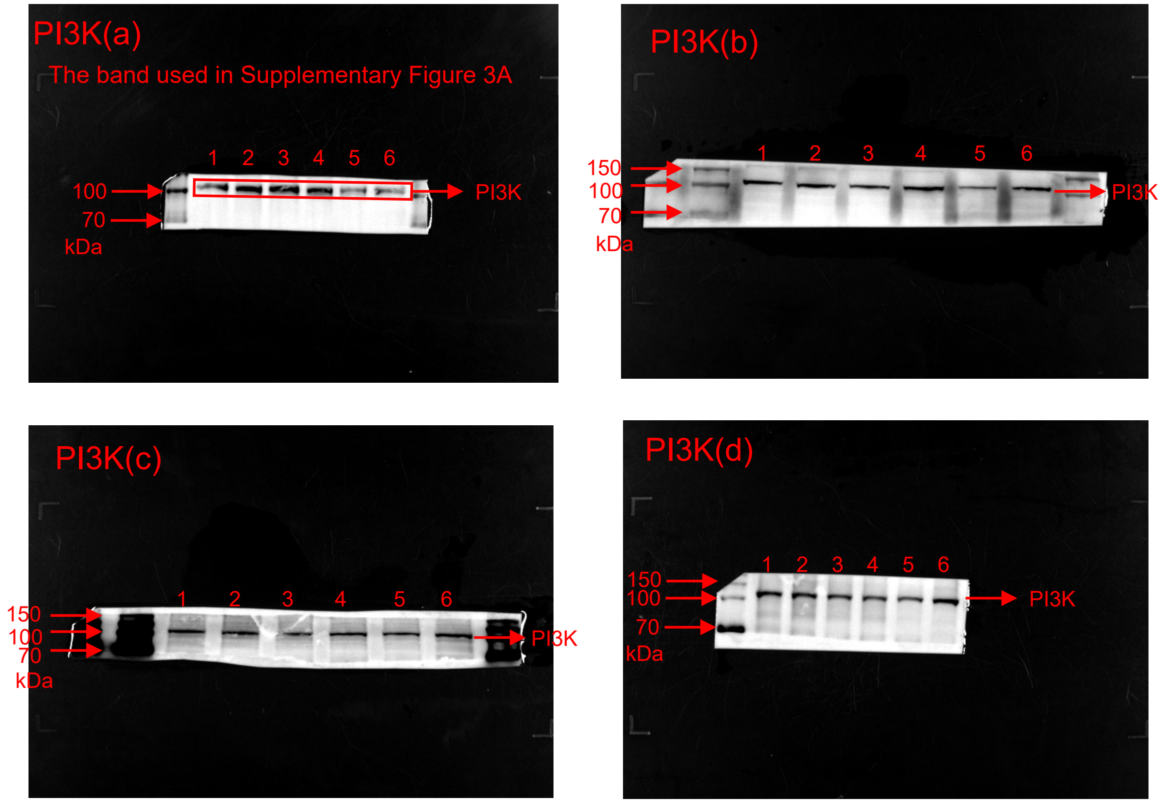
PI3K(a)
The band used in Supplementary Figure 3A
1 2 3 4 5 6
100
70
kDa
PI3K
PI3K(b)
150
100
70
kDa
1 2 3 4 5 6
PI3K
PI3K(c)
150
100
70
kDa
1 2 3 4 5 6
PI3K
PI3K(d)
1 2 3 4 5 6
150
100
70
kDa
PI3K

C. p-AKT

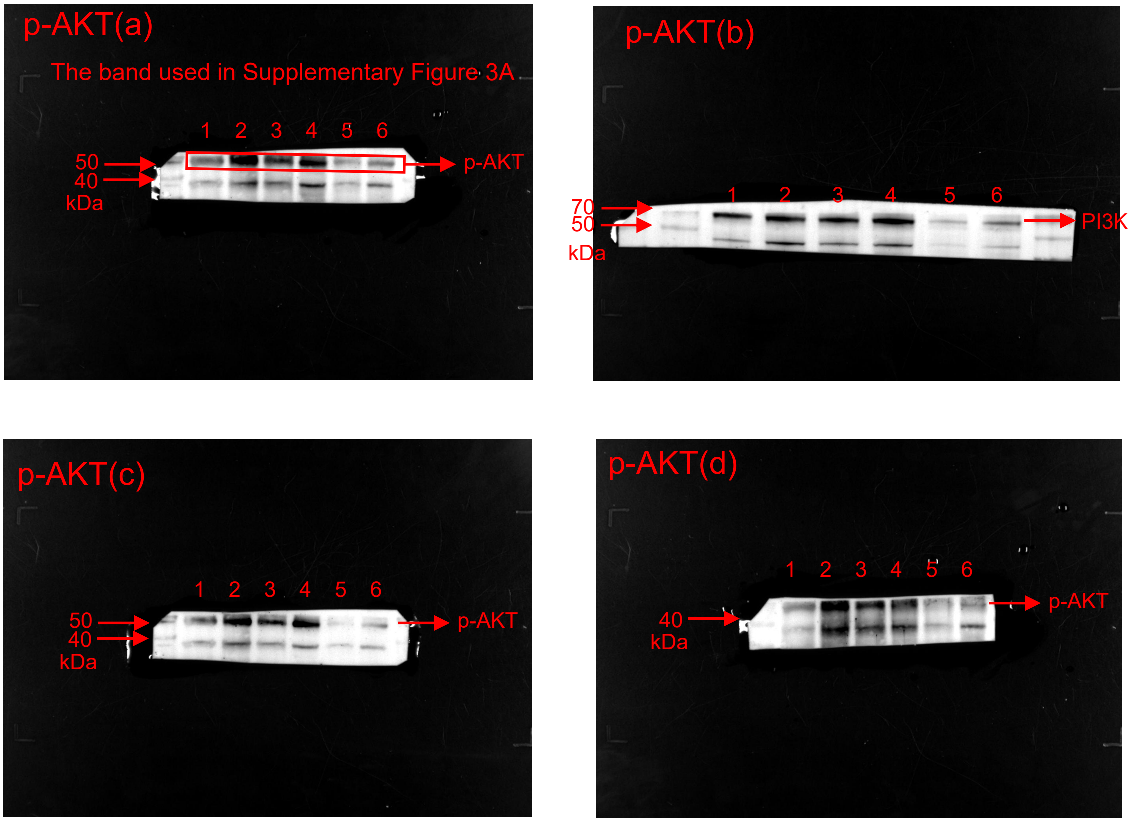
p-AKT(a)
The band used in Supplementary Figure 3A
1 2 3 4 5 6
50
40
kDa
p-AKT
p-AKT(b)
70
50
kDa
1 2 3 4 5 6
PI3K
p-AKT(c)
1 2 3 4 5 6
50
40
kDa
p-AKT
p-AKT(d)
1 2 3 4 5 6
40
kDa
p-AKT

D. AKT

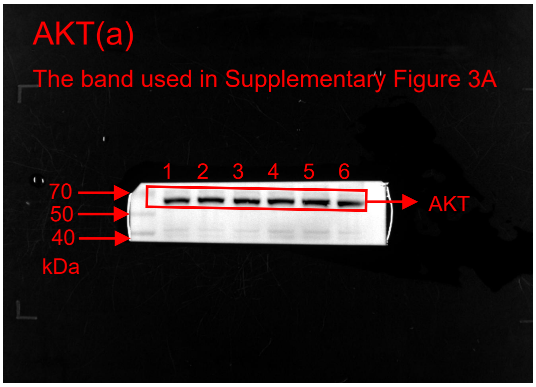

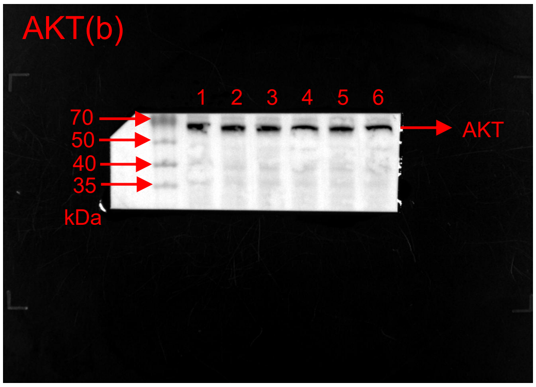

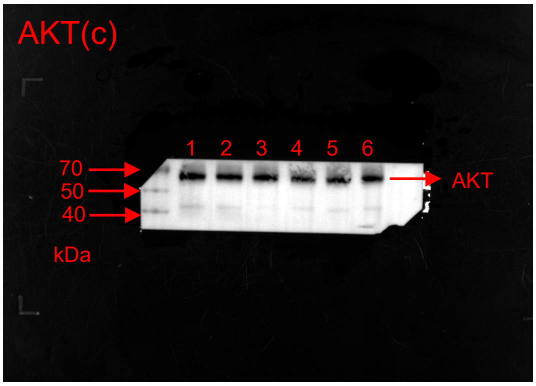

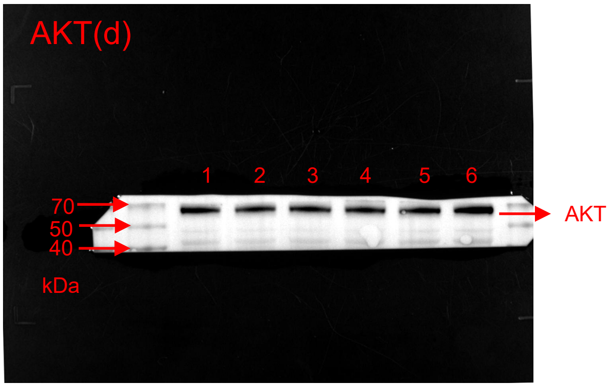

E. Tubulin

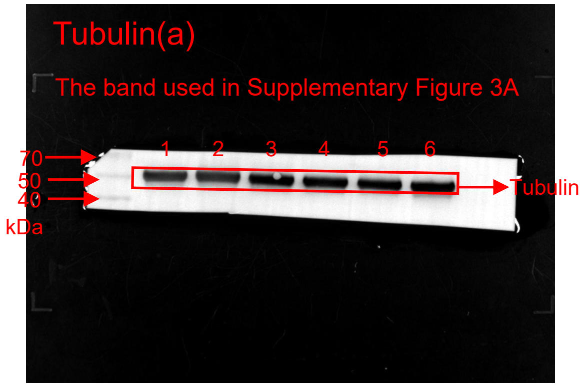

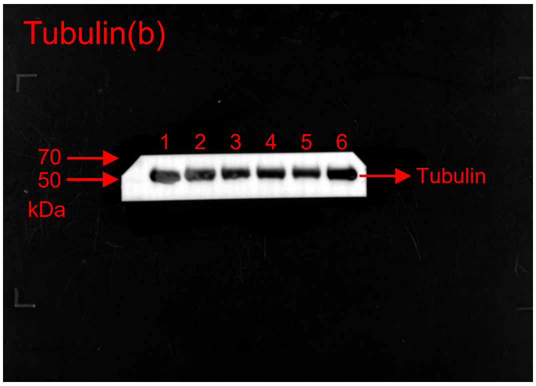

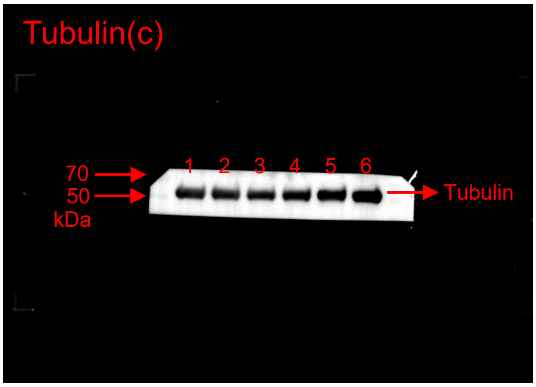

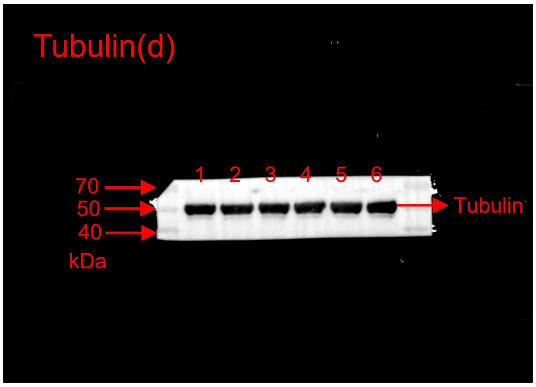

Part Ⅶ. Raw Western blot bands in Supplementary Figure 4A

1: Sham

2: AAV-ShRNA1

3: AAV-ShRNA2

4: AAV-ShRNA3

A. NTF3

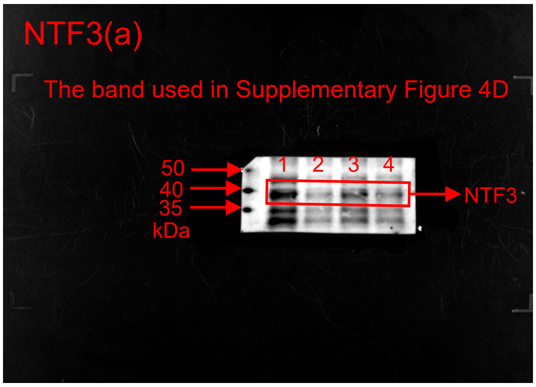

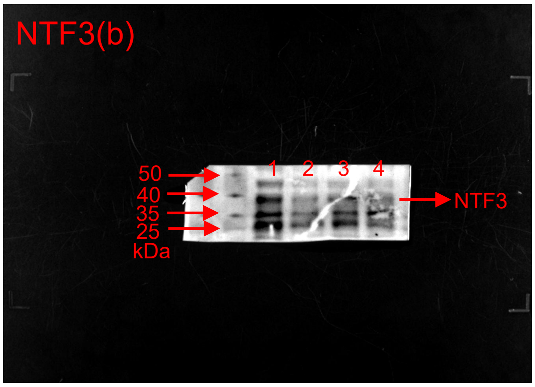

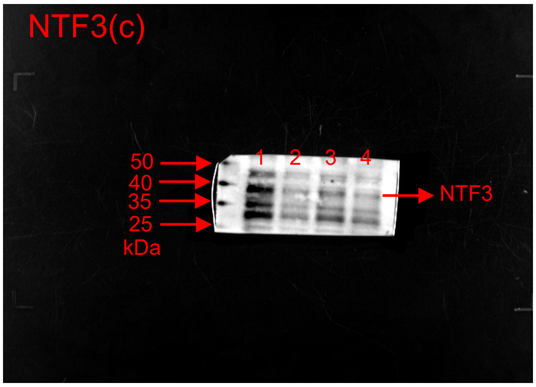

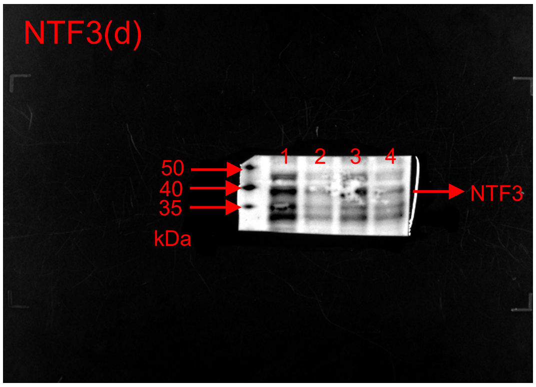

B. Tubulin

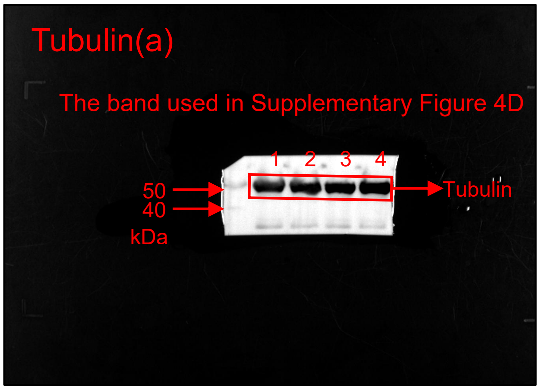

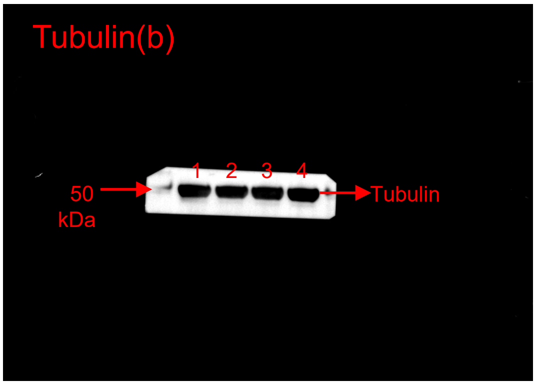

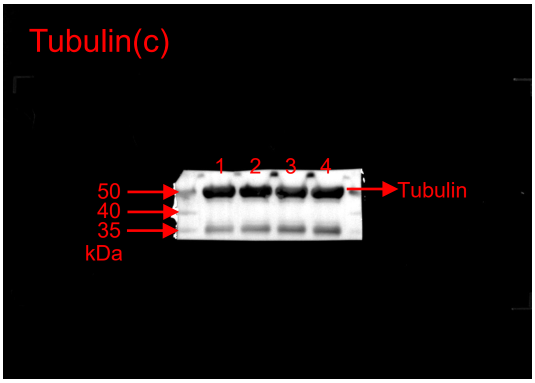

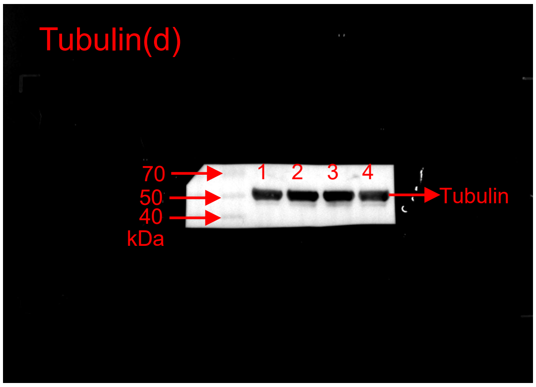

Supplement: Supplementary file 1 — Data S1. [file CNS-30-e70145-s001.zip › Supplemental Files_Raw Western blotting bands.pdf]
