# Supplementary material for: Esketamine Provides Neuroprotection After Intracerebral Hemorrhage in Mice via the NTF3/PI3K/AKT Pathway
Source: CNS Neurosci Ther. 2024 Dec 17;30(12):e70145. doi: 10.1111/cns.70145 (PMC11652676; doi:10.1111/cns.70145)
Supplement: Supplementary file 1 — Data S1. [file CNS-30-e70145-s001.zip › Supplementary material.pdf]

SUPPLEMENTAL MATERIAL

Esketamine provides neuroprotection after intracerebral hemorrhage in mice via the NTF3/PI3K/AKT pathway

Supplementary Methods

1 | Animals

In this study, we used 323 adult male C57BL/6 mice (8-10 weeks, 20-30 g) obtained from Liaoning Changsheng Biotechnology Co., Ltd., license No. SCXK (Liao) 2020-0001). Mice were individually housed in pathogen-free animal facilities at the School of Basic Medical Sciences, Zhengzhou University, with 4-5 mice per cage, provided with sufficient food and water, and subjected to controlled environmental conditions, including temperature (23-25°C), relative humidity (60±10%), and a 12-hour light-dark cycle. We exclusively employed male mice for this study because of the potential behavioral differences caused by sex in mice. The Animal Ethics Committee of Zhengzhou University (ZZUIRB 2022-31) approved all experimental protocols. Animal experiments were performed according to the ARRIVE guidelines (http://www.nc3rs.org.uk/arriveguidelines). All mice were randomly assigned to different groups using the RANDOMIZER FORM (http://www.randomization.com). [20,21]

2 | ICH Mouse Model

The ICH model was established by injecting collagenase into the right striatum of the mice as previously described[22]. Weighed mice were prepared for skin preparation after the induction of anesthesia with 3.5% isoflurane, followed by aseptic manipulation on a stereotactic head frame (RWD Life Science), which was maintained with 1.5% isoflurane in 80% nitrogen and 20% oxygen during the procedure. After an approximately 1 cm incision was made in the mouse scalp with a scalpel, a hole (0.6 mm anterior to the fontanelle and 2.0 mm lateral) was drilled using a cranial drill. A 5μl Hamilton microinjection needle containing collagenase VII-S (0.075 U in 0.5μl of sterile normal saline, Sigma-Aldrich) was slowly introduced 3.5 mm into the needle and injected into the right striatum of the mouse with collagenase at a constant rate of 0.1μl/min. After a 5-minute infusion, the needle was left in place for 10 min to prevent fluid reflux and then removed. Mice in the sham-operated group were injected with the same dose of normal saline using the same procedure. During surgery and recovery from anesthesia, the mice's body temperature was maintained at 37±0.5°C. After awakening from anesthesia, the mice were returned to the cage with the bedding changed.

3 | ESK Intraperitoneal Injection

The ESK injection solution was purchased from Jiangsu Hengrui Medicine Co., Ltd. (Jiangsu, China; 50 mg/2 mL). ESK was dissolved in sterile normal saline

(NS) according to the manufacturer's instructions and injected intraperitoneally at a designated dosage. ESK was administered for five consecutive days after surgery.

4 | Treatment Regimens and Experimental Groups

Experimental 1

Construction of the mouse ICH model Successful construction of the model was evaluated by sectioning fresh tissues fixed in 4% paraformaldehyde (PFA) after ICH and Luxol fast blue/cresyl violet (LFB/CV) and hematoxylin and eosin (HE) staining.

Experiment 2

The effects of ESK on tissue phenotype (brain injury volume and brain water content), neurological impairment, body weight, and behavior after ICH in mice, as well as the optimal drug concentration, were investigated. The experimental animals were randomly classified into five groups according to random number: sham, ICH+Vehicle (NS), ICH + ESK (10 mg/kg), ICH+ ESK (20 mg/kg), and ICH + ESK (40 mg/kg). An optimum concentration of 20 mg/kg was selected for subsequent experiments. Fluoro-Jade C (FJC) and terminal uridine nucleotide end labeling (TUNEL) were performed to examine the neuroprotective effects of ESK in mice with ICH. We evaluated the toxic side effects of the appropriate concentration of ESK in mice using routine blood tests, biochemical parameters of liver and kidney function, body weight, HE

staining, neurology, and behavior to investigate whether the appropriate concentration of ESK has toxic side effects in mice. Routine blood and biochemical parameters of liver and kidney function were categorized into four groups: sham, ICH+Vehicle, ICH + ESK (20 mg/kg), and Sham+ESK (20 mg/kg).

Experiment 3

The potential molecular mechanisms by which ESK improved the prognosis of ICH in mice were also investigated. The mice were randomly classifed into three groups: sham, ICH+Vehicle, and ICH + ESK (20 mg/kg). Target genes and potentially related signaling pathways were identified by sequencing and public database mining.

Experiment 4

Different time courses of NTF3 expression in perihematomal tissues after ICH were detected using western blotting. Cellular localization of NTF3 was detected by double immunofluorescence staining 3 days after ICH. Western blotting was used to detect the expression of proteins related to the effects of NTF3 on the downstream PI3K/AKT pathway-mediated effects in perihematomal tissues after ICH. Correlation analysis was performed on the related receptors NTRK2 and NTRK3 of the ligand NTF3, as well as BDNF, a similar molecule with potential synergistic effects. In this study, the mice were randomly divided into three groups: Sham group, ICH+Vehicle group, and ICH+ESK (20 mg/kg) group.

Experiment 5

We infected the striatum of mice with AAV-siNTF3. ICH models were generated after infection, and mice were randomly classified into five groups: sham, ICH+Vehicle, ICH + ESK (20 mg/kg), ICH + ESK (20 mg/kg)+AAV-negative control (NC), and ICH + ESK (20 mg/kg)+AAV-siNTF3 to assess the effect of NTF3 on brain injury and neurodegeneration after ICH, 28 days before ICH. Neurological function, weight, and related behaviors were assessed preoperatively and on postoperative days 1, 3, and 7. Changes in the expression of important proteins in related signaling pathways were also detected.

Experiment 6

The PI3K inhibitor LY294002 (5 mM) was injected into the lateral ventricle 1 h before ICH injury in each group, and LY294002 (10 mM) was injected intraperitoneally at regular intervals every day after ICH to investigate the potential mechanism by which NTF3 mediates the PI3k/AKT signaling pathway after ICH. The mice were randomly divided into five groups: sham, ICH+Vehicle, ICH + ESK (20 mg/kg), ICH + ESK (20 mg/kg)+DMSO, and ICH + ESK (20 mg/kg)+LY294002. Neurological function, weight, and related behaviors were assessed preoperatively and on postoperative days 1, 3, and 7. Changes in the expression of important proteins in related signaling pathways were also detected (Figure S1).

5 | Mouse Weight Recording

The body weights of the mice were measured and recorded at a fixed time every day before and after surgery. We used the ratio of body weight change as the analysis result to distinguish differences in mice with different body weights. Body Weight Ratio (%) = (body weight at time point/initial body weight) × 100%.

6 | Neurologic Deficit Score

We tested the neurological deficit scores of each mouse on days 1, 3, 5, 7, and 14 after ICH, according to a previously published method The scores were summed for the six subtests: body symmetry, gait, climbing, circling behavior, front limb symmetry, and compulsory circling. Each test score was graded from 0 to 4, with a maximum deficit score of 24. The maximum deficit score of 24 was established. Higher scores indicate more severe neurological impairment.

7 | Behavioral Test

7.1 | Rotarod Test

Motor function in mice after ICH was assessed using an accelerating rotarod. The speed increased steadily from 4 to 40 rpm over 5 min. Adaptive training was performed three days before ICH surgery, three times a day for 5 min, and the speed of the rotarod during the training period was 12 rpm/min at a constant rate. Each mouse was tested three times on postoperative days 1, 3,

7, and 14, with 5-minute intervals between each test. The time taken to fall off the rotating lever was recorded. Data are presented as the mean of three trials.

7.2 | Beam Walking Test

The balance-beam device consisted of a 130 cm long and 1 cm wide and high rod and a unilateral small dark room. The balance beam was 50 cm high, and a cushion was placed underneath to prevent the mice from falling and being injured. The mice were adaptively trained 3 days before ICH surgery, and formal experiments were performed 1, 3, 7, and/or 14 days after surgery to record the walking distance of the mice within a minute on a balance beam. Briefly, 5 points indicated that the participant reached the other end or walked ≥ 45 cm within 30 seconds; 4 points indicated that the participant reached the other end or walked ≥ 45 cm within 60 seconds; 3 points indicated that the participant walked ≥ 22.5 cm within 60 seconds; 2 points indicated that the participant walked < 22.5 cm within 60 seconds; 1 point indicated that the participant did not walk but did not fall within 30 seconds in any posture; and 0 points indicated that the participant fell within 30 seconds.

7.3 | Grid Walking Test (Fault Foot Test)

Mice were placed on a 40 cm × 40 cm overhead grid with a 2.5 cm × 2.5 cm aperture. The number of incorrect hind limb steps (three toes or the entire limb falling below the grid) was proportional to the number of normal steps within 20 steps of a mouse walking 1 day before surgery and on days 1, 3, 7, and/or 14 after surgery. This device was used to detect hind limb sensorimotor loss and

coordination.

8 | Tissue Processing

Bleeding caused by collagenase in the brains of tissue-processed rodents lasts for 6 h and is relatively stable for over three days, followed by partial recovery over time. In this study, we examined brain lesion volume (a combination of hematoma and secondary injury areas) on day 3 after ICH At different time points after ICH surgery in mice, after anesthesia with 3.5% isoflurane, the brains were decapitated immediately. Brain tissues were separated to obtain perihematomal tissues, and normal striatum tissues were collected from the sham operation group. Untreated tissues were frozen at -80°C and subsequently subjected to Western blot, quantitative polymerase chain reaction(qPCR), RNA sequencing, and other experiments. Mice were anesthetized with 3.5% isoflurane and transcardially perfused with saline, followed by 4% PFA on day 3 after ICH. The brains were subsequently removed, stored overnight in 4% paraformaldehyde (PFA), and used for model identification, LFB/CV, HE, FJC, TUNEL, immunofluorescence, and other experiments.

8.1 | Paraffin Section

The sections were then washed with 4% PFA for 1 h overnight for 24 hours and dehydrated at 50%, 70%, 85%, 95%, 100% -I, 100% -II, xylene-I, and xylene-II, followed by embedding and sectioning at a section thickness of 5μm after

immersion in a wax jar for 2 hours.

8.2 | Frozen Section

Next, 4% PFA was added to 20% sucrose and incubated overnight at 24 h, and 30% sucrose was added at 48 h. After the brain samples were submerged in sucrose, they were embedded in the OCT embedding medium at the optimal cutting temperature. The brains were cut into 35μm sections using a cryostat. The sections were stored in a cryopreservation solution at -20°C for immunofluorescence experiments.

9 | LFB/CV and Hematoxylin and Eosin Staining

For staining, the tissue slides were rehydrated using xylene, 100% alcohol, 90%, 80%, 70%, 60%, and 50% alcohol, followed by water. The LFB/CV staining kit (G3245, Solarbio, China) and hematoxylin and eosin HE staining were used according to standard procedures, and the samples were mounted for imaging after clearing.

10 | Brain Lesion Volume

As described above, coronal sections of the entire striatum were stained using the LFB/CV staining kit according to the manufacturer’s instructions: cresyl violet (CV, for Nissl bodies) and Luxol Fast Blue (for myelin). The ImageJ software was used to quantify the bleeding lesion area.

11 | Brain Water Content

Our group and others have shown that vascular permeability peaks three days after collagenase-induced ICH [24]. The mice (n = 6 per group) were anesthetized with isoflurane and decapitated 72 h after ICH. Intact brain tissue was removed and divided into the ipsilateral hemisphere, contralateral hemisphere, and cerebellum (internal controls). The wet weight (WW) of each sample was determined immediately using an electronic analytical balance. The brain samples were dried in an oven at 100°C for 48 h and weighed to determine dry weight (DW). The brain water content was calculated as follows: (WW-DW)/WW × 100%.

12 | FJC Staining

The number of degenerated, apoptotic, and necrotic cells peaked 72 h after ICH in the perihematomal region of rodents. FJC staining was used to quantify degenerating neurons, and the experimental procedures were performed sequentially according to the manufacturer's instructions (G3262, Solarbio, China). Three sections from each mouse were selected to quantify FJC-positive cells, similar to the steps used for immunofluorescence. Stained sections were observed and photographed under a fluorescence microscope at an excitation wavelength of 450-490 nm.

13 | TUNEL Staining

For neuronal death, TUNEL was conducted with a TUNEL Assay Kit (C1089, Beyotime, China) in coronal sections at 72 h after ICH, according to the manufacturer's instructions.

14 | Immunofluorescence Staining

Paraffin sections were rehydrated and subjected to antigen retrieval by heating in sodium citrate-EDTA buffer (P0086, Beyotime, Shanghai, China) at 100°C for 30 minutes. The sections were then blocked with 3% hydrogen peroxide for 10 minutes and then incubated with a primary antibody overnight at 4°C. The following day, the sections were incubated for 1 h at room temperature with the secondary antibody. Then, the sections were stained with the fluorescent dye 4,6-diamidino-2-phenylindole (DAPI, Solarbio, Beijing, China) to label the nuclei and subsequently photographed. The following primary antibodies were used: rabbit anti-NTF3 (1:200, A12476, ABclonal), mouse anti-NeuN (1:500, 66836-1-Ig, Proteintech) mouse anti-GFAP (1:500; GB12100, Servicebio), rabbit anti-MBP (1:200, A11162, ABclonal), rabbit anti-NSE (1:500, 10149-1-ap, Proteintech), mouse anti-Iba1 (1:200; GB12105, Servicebio), rabbit anti-GSK3B (1:200, A11731, ABclonal), rabbit anti-p-GSK3B (1:200, AP1088, ABclonal), rabbit anti-SGK1 (1:200, A1025, ABclonal), and rabbit anti-p-SGK1 (1:200, AB55281, Abcam). The following fluorochrome-conjugated secondary antibodies were used: goat anti-rabbit 488 (1:500, GB25303; Servicebio) and goat anti-mouse Cy3 (1:500, GB21301;

Servicebio).

15 | Transcriptome Sequencing and Pathway Enrichment Analysis

RNA was extracted using the BGISEQ-2000 platform (BGI-Shenzhen, China). RNA was isolated from six tissue samples, namely 3 from the ICH+Vehicle group and three from the ICH+ESK20 group. Differentially expressed genes (DEGs), Kyoto Encyclopedia of Genes and Genomes (KEGG) pathways, and Gene Ontology (GO) data were visualized using the Dr. Tom system provided by BGI. RNA was sequenced using a random sampling process. A gene was considered a differentially expressed gene when the fold change was ≥ 1, and the Q value was ≤ 0.05 to improve the accuracy of the differentially expressed gene results.

16 | Network Pharmacology Validation

16.1 | The Following Main Databases and Software used in this Study

Genecards database (https://www.genecards.org/), DrugBank database (https://www.drugbank.ca/), Search Tool for the Retrieval of Interacting Genes/Proteins (STRING) database (https://string-db.org/), PubChem website (https://pubchem.ncbi.nlm.nih.gov/), Protein Structure Database (Protein Database, PDB, https://www.pdb.org), Cytoscape 3.9.1 software, Open Babel 2.3.2 software, PyMOL 2.3.4 software, AutoDockTools software, and AutoDock Vina 1.1.2 software.

16.2 | Mining of ESK and ICH Targets

Information on ICH disease targets was obtained by searching the GeneCards and DrugBank databases using the keyword "ICH," and information on the targets of ESK was downloaded from the DrugBank database using the keyword "esketamine." The intersection of the targets of ICH and ESK was determined by constructing Venn diagrams to identify targets common to drugs and diseases.

16.3 | Construction of a Protein Interaction Network and KEGG Pathway Enrichment Analysis of ICH Targets for ESK Therapy

The intersection target genes were uploaded to the online STRING database, and the protein species used were set as "*Homo sapiens* (human)" in the database to construct a Protein–protein interaction (PPI) network diagram. The corresponding protein interaction information was obtained and downloaded, important target proteins were screened according to the number of targets in the PPI network for analysis, and the results were plotted using Cytoscape 3.9.1 software. Simultaneously, the STRING database can be used to perform functional annotation and enrichment analyses based on the key target proteins common to ESK and ICH and the significantly related signaling pathways that can be identified.

16.4 | Molecular Docking Calculation of Drug Affinity for Targets

The compound structure files were obtained by a PubChem website search, and the SDF files were converted into PDB files using the Open Babel 2.3.2

software. Receptor proteins were retrieved from the Protein Structure Database (PDB), and three-dimensional structures of protein targets were obtained. The receptor protein was dehydrated and the ligand was removed using PyMOL 2.3.4 software. The receptor protein was hydrogenated, and the balanced charge was modified using AutoDockTools software to transform the receptor protein and ligand small molecules into the pdbqt format. Global molecular docking of receptor proteins to small ligand molecules was performed using AutoDock Vina 1.1.2. Docking results were visualized using PyMOL, and the affinity was recorded.

17 | Quantitative Polymerase Chain Reaction (qPCR) Array

Following tissue grinding, total RNA was extracted using TRIzol reagent according to the manufacturer’s instructions. DNA was amplified using the MLV RT Premix for qPCR (Accurate Biology, AG11706, China). The specific reaction conditions were 37°C, 15 min, 85°C, 5 s, and 4°C for 10 min, and real-time PCR (qPCR) was performed using SYBR Green. A SYBR Green Premix Pro Tag HS qPCR Kit (Rox Plus) (Accurate Biology, AG11718, China) and a CFX Connect real-time PCR system (BioRad, Hercules, CA, USA) were used at 95°C for 15 s, followed by 40 cycles at 95°C, 5 s and 60°C for 34 s. The data were analyzed using the same method. The primers used for mRNA were as follows: NTF3-F, “GGAGTTTGCCGGAAGACTCTC”; NTF3-R, “GGGTGCTCTGGTAATTTTCCTTA”; GAPDH-F, “AGGGCCCT

ATCCCAACTCG” and GAPDH-R, “GGGGTCGTTGATGGCAACA.”

18 | Western Blot

Total protein was extracted using RIPA lysis buffer, protease inhibitor, and phosphatase inhibitor mixing (RIPA/PMSF, 100:1; Solarbio, Beijing, China) lysis of tissue and then quantified using a BCA protein assay kit (Solarbio). Protein samples were heated to 100°C for 7 min. Equal amounts of protein samples were separated using 15% sodium dodecyl sulfate (SDS)-sulfate-polyacrylamide gel electrophoresis (PAGE) and transferred to polyvinylidene fluoride (PVDF) membranes. The membranes were blocked with Quick Blocking Solution (Epizyme Biotech, Shanghai, Beijing, China) for 10 min and incubated overnight at 4°C with primary antibodies. The primary antibodies used were rabbit anti-NTF3 (1:1000, A12476, ABclonal), rabbit anti-BDNF(1:1000, A4873, ABclonal), mouse anti-NeuN (1:1000, 66836-1-lg, Proteintech), mouse anti-GFAP (1:1000; GB12100, Servicebio), rabbit anti-MBP (1:1000, A11162, ABclonal), rabbit anti-NSE (1:10000, 10149-1-ap, Proteintech), mouse anti-Iba1 (1:1000; GB12105, Servicebio), rabbit anti-GSK3B (1:1000, A11731, ABclonal), rabbit anti-p-GSK3B (1:1000, AP1088, ABclonal), rabbit anti-SGK1 (1:1000, A1025, ABclonal), rabbit anti-p-SGK1 (1:1000, AB55281, Abcam), mouse anti-PI3K (1:1000, 67071-1-lg, Proteintech), rabbit anti-AKT (1:1000, A11016, ABclonal), rabbit anti-p-AKT (1:1000, AP0637, ABclonal), rabbit anti-TrkB (1:10000, A21227, ABclonal),

rabbit anti-TrkC (1:1000, A23002, ABclonal), rabbit anti- p-TrkB (1:1000, AF3461, Affinity), rabbit anti- p-TrkC (1:1000, AF7405, Affinity). After overnight the membranes were washed five times for 6 min each and incubated with the appropriate secondary antibody, goat anti-mouse (1:5000; SA00001-1, Proteintech) or goat anti-rabbit (1:5000; SA00001-2, Proteintech) for 1.5 h at 37°C. Protein signals were visualized using an enhanced chemiluminescence(ECL) kit (Beyotime Biotechnology, Shanghai, China) and semi-quantitatively analyzed using ImageJ software. The results are expressed as the relative density minus the background and normalized to the loading control internal reference protein.

19 | Blood Sample Processing for Routine Blood and Biochemical Tests

Blood Specimen Collection

Mice in different groups were anesthetized with 1% pentobarbital sodium, and whole blood (0.5 ml of whole blood was collected from the orbital venous plexus in EDTA K2 anticoagulant tubes and 1.5 ml centrifuge tubes, which were centrifuged at 3,000 rpm for 10 min. Processed whole blood and serum samples were sent to Servicebio Technology Co., Ltd. (Wuhan, China) for the next step of testing.

19.1 | Routine Blood Tests

Routine blood parameters of the mice, including white blood cell (WBC) count, neutrophil (Gran) count, lymphocyte (Lymph) count, monocyte (Mon) count,

neutrophil ratio (Gran%), lymphocyte ratio (Lymph%), monocyte ratio (Mon%), red blood cell (RBC), hemoglobin (HGB), hematocrit (HCT), mean corpuscular volume (MCV), mean corpuscular hemoglobin (MCH), mean corpuscular hemoglobin concentration (MCHC), red blood cell distribution width (RDW), platelet (PLT), platelet volume (PCT), mean platelet volume (MPV), and mean platelet distribution width (PDW), were measured by an automatic hematology analyzer.

19.2 | Blood Biochemical Indicators

Serum biochemical parameters, including alanine aminotransferase (ALT), aspartate aminotransferase (AST), direct bilirubin (D-BIL), total bilirubin (T-BIL), glutamyl transpeptidase (GGT), total bile acid (TBA), alkaline phosphatase (ALP), albumin (ALB), blood urea nitrogen (BUN), creatinine (CREA), and uric acid (UA), were measured using an automatic biochemical analyzer.

20 | Adeno-Associated Virus

Adeno-associated viruses were injected into the lateral ventricle as previously described by microinjection. Mice were infected with AAV to knock down NTF3 (AAV2/9-U6-shRNANTF3-WPRE, 2μl, 1 × 10^{13} V.G/ml) at the ICH site 4 weeks before ICH induction. AAVs were constructed using the targeting assistance provided by Hanbio Co., Inc. (Shanghai, China). The sequence of the siRNA used was 5’ -GCATTGCCA TTTCCACATA-3.’

21 | LY294002 Intraperitoneal and Intracerebroventricular Injection

The PI3K/AKT signaling pathway-specific inhibitor LY294002 was obtained from MedChemExpress (Monmouth Junction, NJ, USA).2ul of LY294002 (10 nmol/2μl) dissolved in 25% DMSO was infused 30 min before ICH induction. LY294002 (8 mg/kg) was dissolved in NS or dimethyl sulfoxide (DMSO) and administered by intraperitoneal injection for 3 consecutive days after surgery. Briefly, a 32G needle with a 5μl Hamilton syringe was inserted into the right lateral ventricle through a burr hole in the skull at the following coordinates relative to the bregma: 0.3 mm posterior, 1.0 mm lateral, and 2.3 mm deep. Intraventricular administration was performed using a micro-infusion pump at a rate of 0.2μl/min. After infusion, the needle was left in place for an additional 10 min before removal. The drill holes were sealed with bone wax.

22 | Statistical Analysis

Mice that died during surgery or shortly after ICH were excluded from the final analysis. The normality of data distribution was assessed using the Shapiro–Wilk test. Data are expressed as mean ± standard deviation. For comparisons between two groups, unpaired t-tests were used. Repeated-measures analysis of variance (ANOVA) with Bonferroni post hoc correction was applied to data collected at different time points across groups. One-way ANOVA with Bonferroni post hoc correction was used for comparisons among multiple groups, while the Friedman or Kruskal-Wallis test

was employed for non-normally distributed data. Mortality rates were compared using the Chi-square test. Statistical analyses were conducted with GraphPad Prism 9.5.0 (GraphPad Software, San Diego, CA, USA; www.graphpad.com). Statistical significance was set at $P < 0.05$.

Supplementary Figure

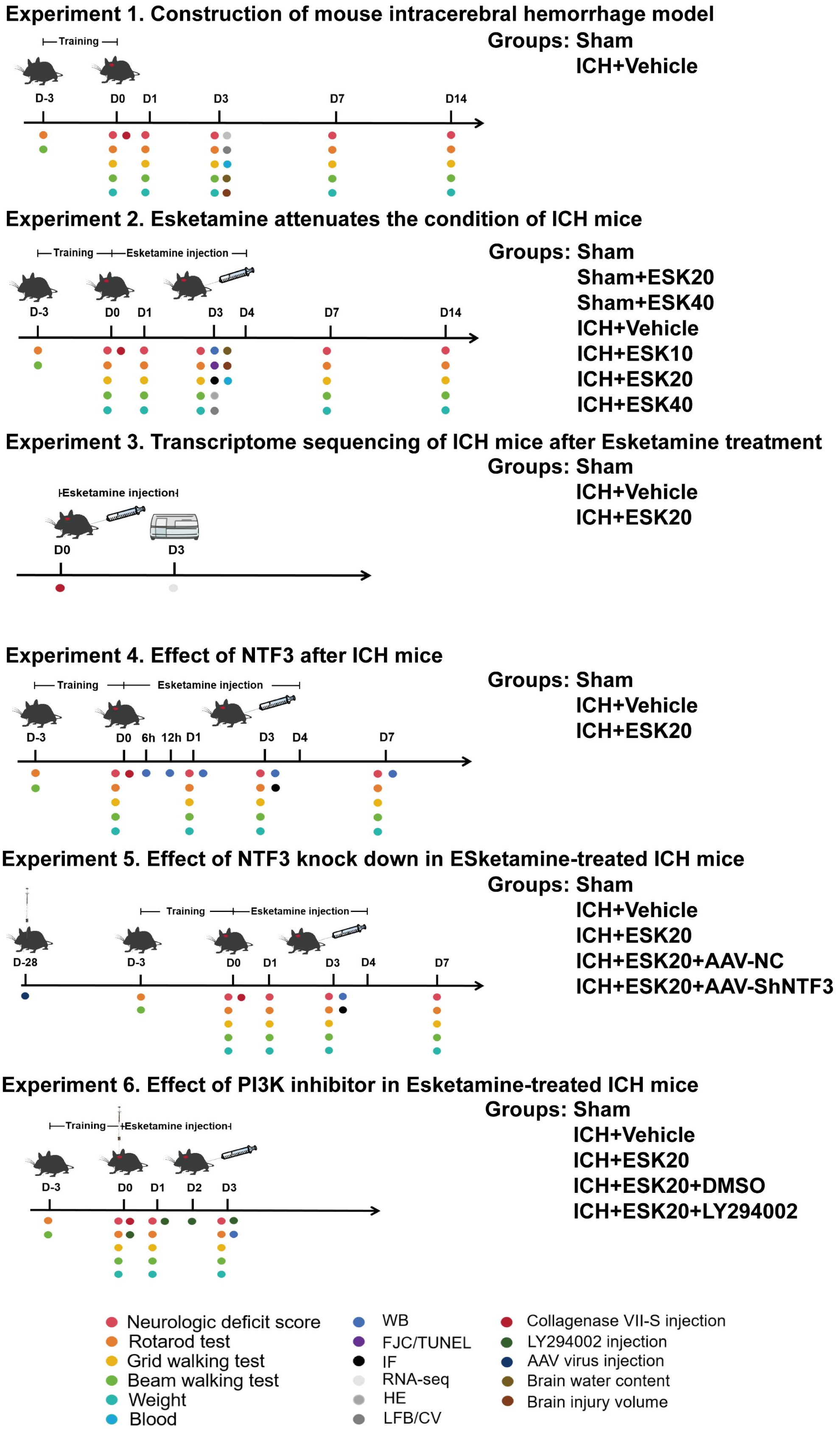

Supplementary Figure 1. Experimental design and animal groups.

DMSO, Dimethyl sulfoxide; ESK (10/20/40), Esketamine (10 mg/kg, 20 mg/kg, 40 mg/kg); FJC, Fluoro-Jade C; ICH, Intracerebral hemorrhage; HE, Hematoxylin-eosin staining; IF, Immunofluorescence; LFB/CV, Luxol fast blue/crystal violet; TUNEL, Terminal deoxynucleotidyl transferase-mediated dUTP-biotin nick end labeling; RNA-seq, RNA sequencing; WB, Western blot.

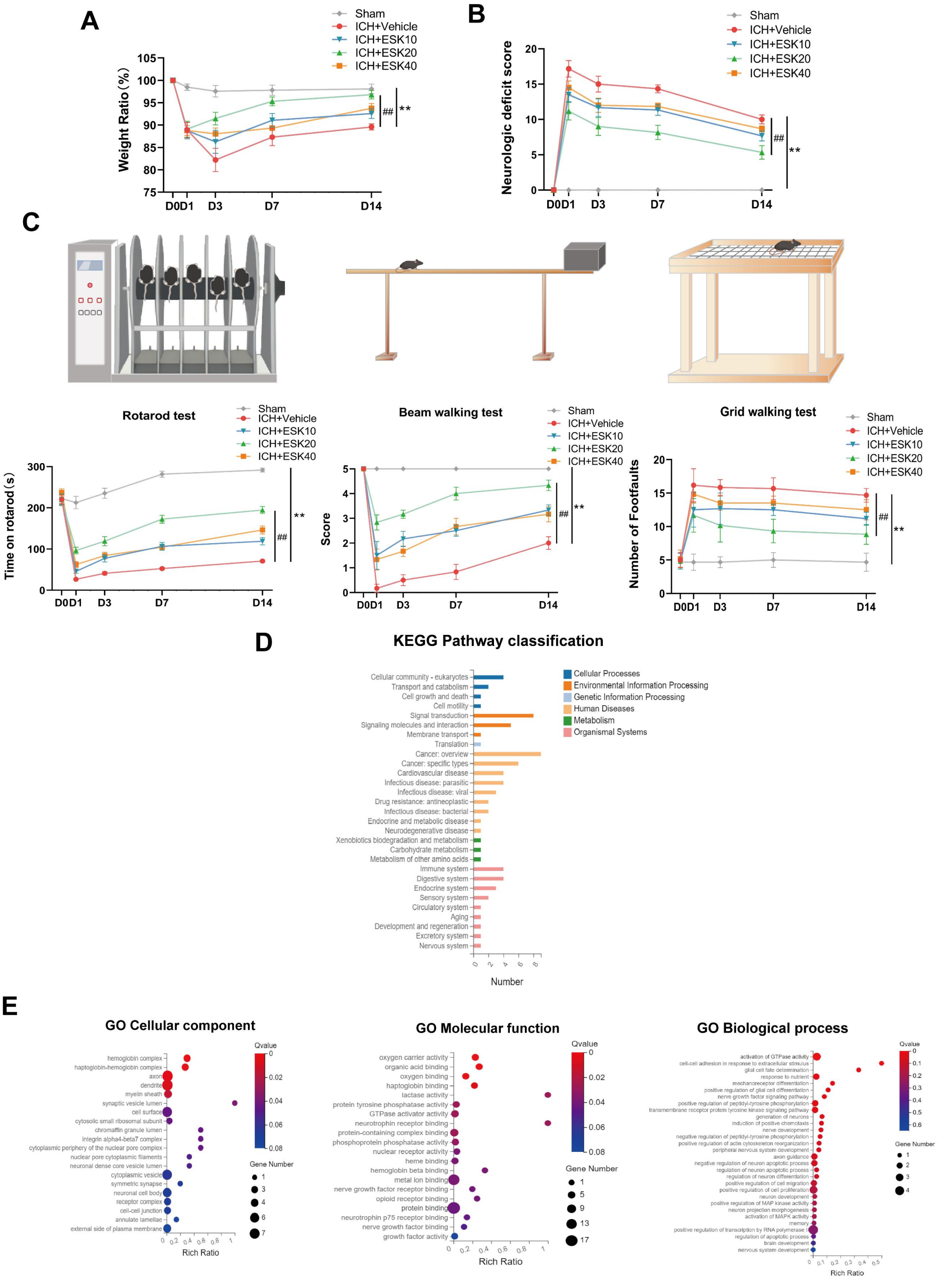

Supplementary Figure 2. Drug dose screening and related bioinformatics analysis

(A) Body weight ratio, **(B)** Neurologic deficit score, and **(C)** Behavioral assessments (rotarod test, beam walking test, and grid walking test). $^{*}P < 0.05$, $^{**}P < 0.01$: ICH+Vehicle vs. sham; $^{\#}P < 0.05$, $^{\#\#}P < 0.01$: ICH+ESK20 vs. ICH+Vehicle(n = 6). **(D)** KEGG pathway classification enrichment map, where a larger p-value (-log10) indicates a greater degree of enrichment. **(E)** GO enrichment of the commonly detected DEGs, which included molecular function, cellular components, and biological processes.

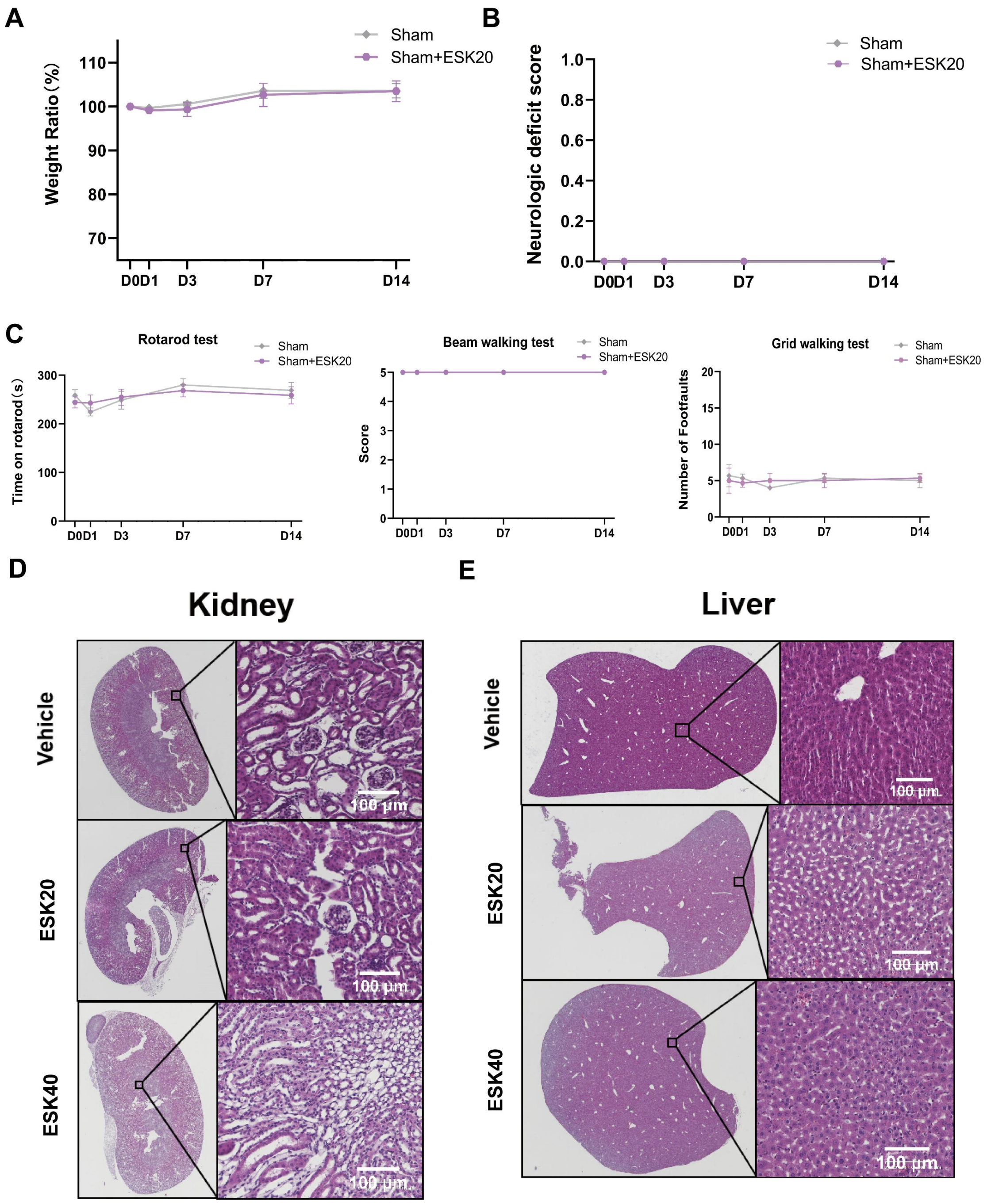

Supplementary Figure 3. Effects of 20 mg/kg ESK on mouse weight and neurological and motor function in mice on days 1, 3, 7 and/or 14.

(A) Body weight ratio; **(B)** Neurologic deficit score; and **(C)** Behavioral test results (rotarod test, beam walking test, and grid walking test) (n = 3). **(D, E)**

HE staining of liver and kidney tissues from mice treated with different doses of ESK for 4 days (n = 3, scale bar:100 μm, 20X magnification of the right panel in each group).

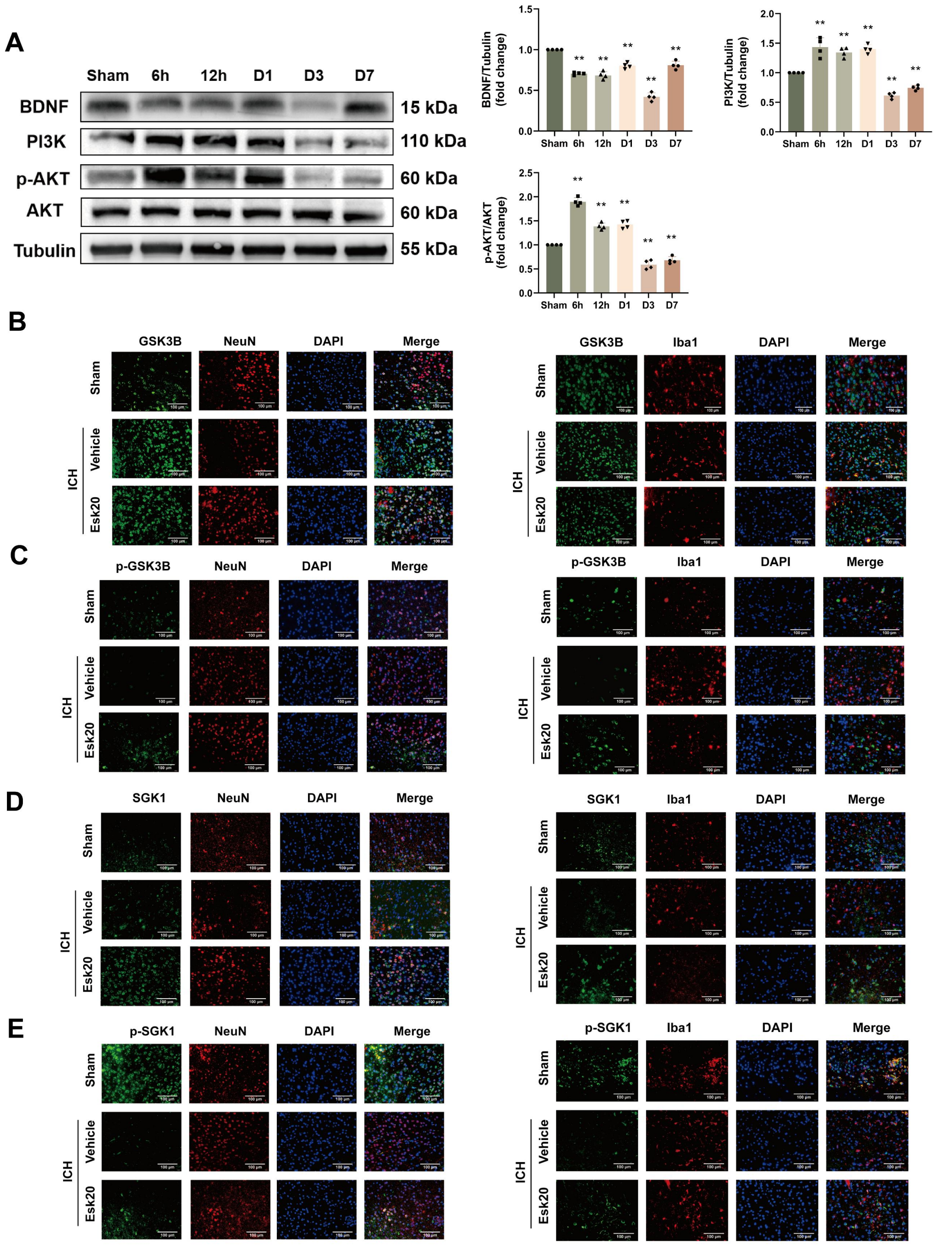

Supplementary Figure 4. Time course of BDNF, PI3K, and p-AKT protein levels after ICH and the cellular location of BDNF, PI3K, and p-AKT after ICH.

(A-D) Representative western blot bands and quantitative analyses of BDNF, PI3K, p-AKT, and AKT expression in the ipsilateral hemisphere after ICH (n = 4). ***P* < 0.01 vs sham. **(E-L)** Representative images of double immunofluorescence staining showing that GSK3B, p-GSK3B, SGK1, and p-SGK1 (green) were colocalized with neurons or microglia (red for NeuN or Iba1) in the perihematomal brain area of the ICH+Vehicle and ICH+ESK 20 groups and the corresponding brain area in the sham group at 72 hours after ICH. Scale bar:100 μm (n = 3).

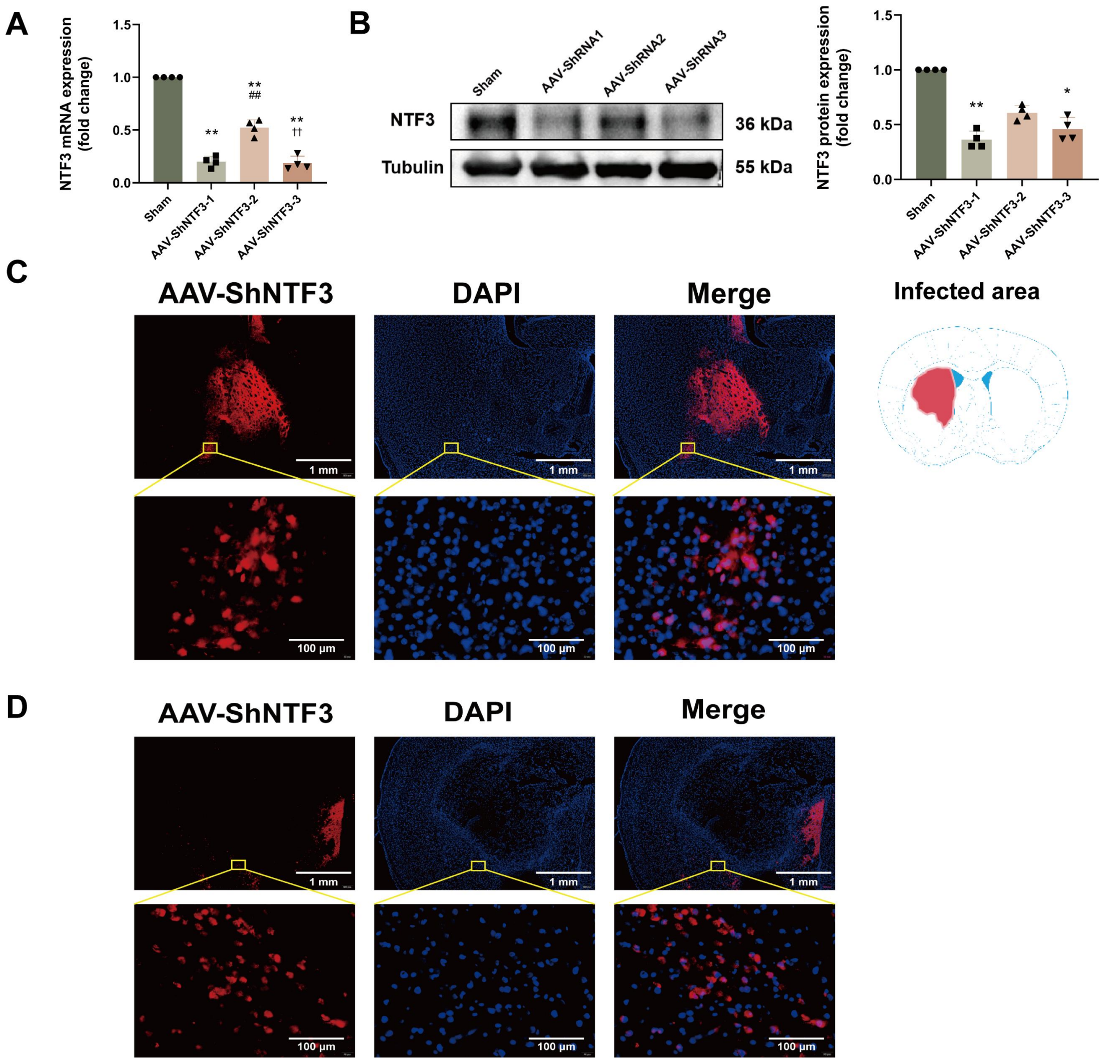

Supplementary Figure 5. The striatum infection in mice 28 days after AAV infection.

(A) QPCR results reveal the mRNA expression of three different AAV-ShNTF3 sequences after NTF3 knockout. (n = 4. $^{**}P < 0.01$, sham; $^{\#\#}P < 0.01$ vs. AAV-ShNTF3-1; $^{\dagger\dagger}P < 0.01$, AAV-ShNTF3-2). **(B, C)** Western blot analysis revealed the protein levels of three different AAV-ShNTF3 sequences after NTF3 knockout. (n = 4. $^{*}P < 0.05$, $^{**}P < 0.01$, vs. Sham). **(D)** Fluorescence of mice in the Sham group 28 days after AAV infection (n=3, Scale bar:1 mm/100

μm); **(E)** Fluorescence of 3 days after ICH in mice infected with AAV infection (n=3, Scale bar:1 mm/100 μm).

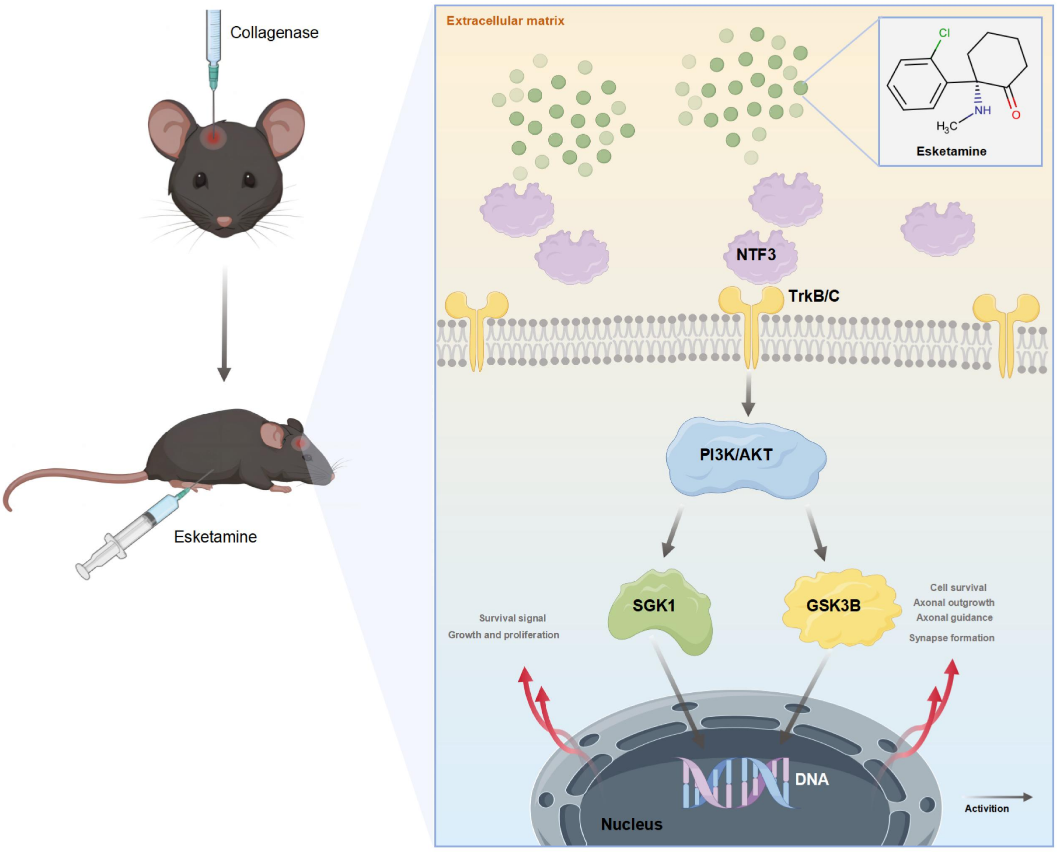

Supplementary Figure 6. Graphic abstract

Schematic representation of the underlying molecular mechanisms by which ESK mediates neuroprotection of the PI3K/AKT signaling pathway in mice with ICH through activation of NTF3 expression.

Summary of experimental groups, sample sizes, and mortality rate in the study

Experimental Groups	Weight<br>Neurological test<br>Behavior test	Brain water content	Injury volume	FJC<br>TUNEL	RNA-seq	IF	WB	Mortality	Subtotal
**Experiment 2**									
Sham	6	6	6	3		3	5	0	29
ICH+Vehicle(normal saline)	6	6	6	3		3	5	6	35
ICH+Esketamine(10mg/kg)	6							3	9
ICH+Esketamine(20mg/kg)	6	6	6	3		3	5	1	30
ICH+Esketamine(40mg/kg)	6							4	10
Sham+Esketamine(20mg/kg)	3								3
Sham+Esketamine(40mg/kg)	3								3
**Experiment 3**									0
Sham					3			0	3
ICH+Vehicle(normal saline)					3			1	4
ICH+Esketamine(20mg/kg)					3			0	3
**Experiment 4**									0
Sham	6					3	5	0	14
ICH+Vehicle(normal saline)	6					3	5	1	15
ICH+Esketamine(20mg/kg)	6					3	5	0	14
**Experiment 5**									0
Sham	6					3	5	0	14
ICH+Vehicle(normal saline)	6					3	5	2	16
ICH+Esketamine(20mg/kg)	6					3	5	1	15
ICH+ESK20+AAV-NC	6					3	5	1	15
ICH+ESK20+AAV-ShNTF3	6					3	5	2	16
**Experiment 6**									0
Sham	6					3	5	0	14
ICH+Vehicle(normal saline)	6					3	5	2	16
ICH+Esketamine(20mg/kg)	6					3	5	0	14
ICH+ESK20+DMSO	6					3	5	0	14
ICH+ESK20+LY294002	6					3	5	3	17
**Total**	114	18	18	9	9	48	80	27	323

ICH, intracerebral hemorrhage. DMSO, Dimethyl sulfoxide. ESK20, Esketamine(20mg/kg).

Differential gene expression levels

GeneSymbol	log2 (ICH+ESK20/ICH)	Qvalue (ICH+ESK20/ICH)
Gm40365	8.999534542	6.52724E-07
LCT	6.008777802	7.07079E-05
NTF3	3.395613114	0.013784383
CXCL9	3.015654997	0.037655887
GBP10	2.739852271	0.024109354
TAFA1	1.808695209	0.007196684
HBA-a1	1.740663499	0.000203535
HBB-bs	1.47190688	0.000238499
NR4A3	1.445573143	0.033477756
NWD2	1.424563015	0.000959826
TNFRSF25	1.420486914	0.028900198
HBA-a2	1.240869874	0.042027434
LAG3	1.019516697	0.002140105
NECTIN3	1.013688211	0.013784383
FLT4	-1.21686994	0.046182544

Prediction of relevant signaling pathways and key genes

Term	FDR	Genes
Neurotrophin signaling pathway	1.36759E-15	NTRK1, NGFR, NTRK2, SHC3, SHC1, SORT1, BDNF, CAMK2A, FRS2, PTPN11, NGF, NTF4, NTF3, PLCG1
Ras signaling pathway	1.34527E-13	NTRK1, NGFR, NTRK2, SHC3, SHC1, BDNF, RASGRF1, PTPN11, NGF, GRIN2B, GRIN1, NTF4, GRIN2A, NTF3, PLCG1
Calcium signaling pathway	0.000356814	NTRK1, NTRK2, GRIN2A, CAMK2A, PLCG1, NOS1, NGF, GRIN1
Cocaine addiction	0.000512041	GRIN2A, BDNF, DLG4, GRIN2B, GRIN1
Alcoholism	0.000602472	NTRK2, GRIN2A, SHC3, SHC1, BDNF, GRIN2B, GRIN1
MAPK signaling pathway	0.000666495	NTRK1, NTF4, NTRK2, NGFR, BDNF, RASGRF1, NTF3, NGF
Rap1 signaling pathway	0.000826998	NGFR, GRIN2A, SRC, PLCG1, NGF, GRIN2B, GRIN1
ErbB signaling pathway	0.002254438	SHC3, SHC1, SRC, CAMK2A, PLCG1
Circadian entrainment	0.003123911	GRIN2A, CAMK2A, NOS1, GRIN2B, GRIN1
Inflammatory mediator regulation of TRP channels	0.003123911	NTRK1, SRC, CAMK2A, PLCG1, NGF
Pathways of neurodegeneration - multiple diseases	0.006768514	GRIN2A, BDNF, DLG4, CAMK2A, PLCG1, NOS1, GRIN2B, GRIN1
Natural killer cell mediated cytotoxicity	0.006768514	SHC3, SHC1, PTPN11, FYN, PLCG1
PI3K-Akt signaling pathway	0.00760173	NTRK1, NTF4, NTRK2, NGFR, BDNF, NTF3, NGF
Phospholipase D signaling pathway	0.01002471	SHC3, SHC1, PTPN11, FYN, PLCG1
Long-term potentiation	0.01002471	GRIN2A, CAMK2A, GRIN2B, GRIN1
Amphetamine addiction	0.010233409	GRIN2A, CAMK2A, GRIN2B, GRIN1
Drug metabolism - cytochrome P450	0.010890871	CYP2C9, CYP2B6, CYP2C19, CYP3A4
Glioma	0.011568624	SHC3, SHC1, CAMK2A, PLCG1
EGFR tyrosine kinase inhibitor resistance	0.012721907	SHC3, SHC1, SRC, PLCG1
Axon guidance	0.015799411	SRC, CAMK2A, PTPN11, FYN, PLCG1
Linoleic acid metabolism	0.020529003	CYP2C9, CYP2C19, CYP3A4
Focal adhesion	0.020529003	SHC3, SHC1, SRC, RASGRF1, FYN
Proteoglycans in cancer	0.021067013	SRC, CAMK2A, FRS2, PTPN11, PLCG1
Lipid and atherosclerosis	0.023910536	CYP2C9, CYP2B6, SRC, CAMK2A, PLCG1
cAMP signaling pathway	0.025298161	GRIN2A, BDNF, CAMK2A, GRIN2B, GRIN1
Glutamatergic synapse	0.026164844	GRIN2A, DLG4, GRIN2B, GRIN1
Nicotine addiction	0.031362694	GRIN2A, GRIN2B, GRIN1
Relaxin signaling pathway	0.034163085	SHC3, SHC1, SRC, NOS1

Blood routine results of mice

Index	Sham	Sham+ESK20	ICH+Vehicle	ICH+ESK20
WBC（10^9/L）	5.37±0.42	6.77±1.07	3.53±2.14	3.27 ±1.93
Lymph（10^9/L）	4.37±0.25	5.33±1	2.93±1.87	2.60 ±1.54
Mon（10^9/L）	0.17±0.12	0.23±0.06	0.1±0.1	0.07 ±0.06
Gran（10^9/L）	0.83±0.15	1.2±0.2	0.5±0.2 **	0.60 ±0.35
Lymph(%)	81.63±4.02	78.23±2.87	78.7±8.93	79.13 ±2.66
Mon(%)	3.33±1.97	3.9±1.66	4.17±1.01	2.70 ±0.20
Gran(%)	15.03±2.06	17.87±1.96	17.13±8.92	18.17 ±2.80
RBC(10^{12}/L)	7.1±0.38	6.93±0.61	6.45±0.36	6.69 ±0.19
HGB(g/L)	104.33±5.51	105±10	99.33±6.35	103.00 ±2.00
HCT(%)	31.03±1.48	29.53±3	27.77±1.55	28.87 ±0.42
MCV(fL)	43.8±1.08	42.67±0.57	43.13±0.5	43.27 ±0.64
MCH(pg)	14.63±1.17	15.07±0.12	15.33±0.15	15.37 ±0.21
MCHC（g/L）	336±19.31	355±2.65	357±4.58	356.33 ±2.31
RDW(%)	15.83±1.07	15.7±1.4	15.9±1.67	14.60 ±1.23
PLT（10^9/L）	493.67±89.03	656.33±92.97	703.33±124.74	733.67 ±172.26
MPV(fL)	5.07±0.21	4.8±0.17	4.87±0.12	4.77 ±0.15
PDW	16.1±0.3	15.63±0.15	15.67±0.23	15.57± 0.12
PCT(%)	0.25±0.05	0.31±0.03	0.34±0.07	0.35 ±0.09

* and ** represent significant difference and highly significant differences between the ICH+Vehicle group and the Sham+ESK20 group

Blood biochemical results of mice

Index	Sham	Sham+ESK20	ICH+Vehicle	ICH+ESK20
ALT（U/L）	44.58 ± 8.00	45.28 ± 3.91	51.93 ± 19.19	46.19 ± 12.8
AST（U/L）	133.72 ± 38.86	134.50 ± 10.62	176.42 ± 59.53	136.89 ± 44.8
D-BIL（umol/l）	9.44 ± 1.09	12.94 ± 6.63	16.22 ± 4.46	7.47 ± 3.72
T-BIL（umol/l）	18.38 ± 4.62	17.88 ± 9.50	23.28 ± 1.58 *	11.53 ± 3.36
ALB（g/L）	27.08 ± 2.01	24.73 ± 1.42	26.05 ± 1.30	24.82 ± 0.21
ALP（U/L）	124.89 ± 22.39	85.03 ± 7.19	82.87 ± 12.65	73.59 ± 6.83
GGT（U/L）	0.87 ± 0.06	0.89 ± 0.09	0.89 ± 0.09	0.94 ± 0.01
TBA（umol/l）	3.16 ± 1.24	4.12 ± 1.67	4.87 ± 0.61	3.23 ± 1.83
BUN（mg/dL）	26.06 ± 3.01	23.22 ± 0.76	22.75 ± 3.97	23.27 ± 0.96
CR（umol/l）	21.60 ± 3.61	27.68 ± 12.53	27.23 ± 11.36	21.16 ± 3.24
UA（umol/l）	130.89 ± 35.41	158.68 ± 53.01	200.90 ± 27.05	143.58 ± 42.47

* and ** represent significant difference and highly significant differences between the ICH+Vehicle group and the ICH+ESK20 group

Table S6 Statistics reporting

Figure	N (sample size)	Data Structure	Variance Homogeneity	Test used	*p* Value
Figure 1B<br>(Brain water content<br>on day 3)	Sham,n=6<br>ICH+Vehicle,n=6<br>ICH+ESK20,n=6	Normal distribution	Equal variance	One-way ANOVA; Bonferroni *post hoc*	Ipsilateral: $p<0.001$.<br>ICH+ESK20 vs. ICH+vehicle,$p=0.6543$<br>ICH+vehicle vs. Sham,$p<0.001$<br><br>Contralateral: $p=0.035$.<br>ICH+ESK20 vs. ICH +vehicle,$p=1.000$<br>ICH+vehicle vs. Sham,$p=0.040$<br><br>Cerebellum: $p=0.486$.<br>ICH+ESK20 vs. ICH+vehicle,$p=1.000$<br>ICH+vehicle vs. Sham,$p=0.722$
Figure 1B<br>(Brain injury volume<br>on day 3)	ICH+Vehicle,n=6<br>ICH+ESK20,n=6	Normal distribution	Equal variance	Student's *t*-test	$p=0.2985$

Figure 1D (Weight Ratio on days 0,1,3,7 and 14)	Sham,n=6 ICH+Vehicle,n=6 ICH+ESK20,n=6	Normal distribution	Equal variance	Repeated measures ANOVA; Bonferroni *post hoc*	*P*<0.001. ICH+ESK20 vs. ICH+vehicle,*p*<0.001 ICH + vehicle vs. Sham,*p*<0.001
Figure 1E (Neurologic deficit score on days 0,1,3 and 7)		Normal distribution	Equal variance	Repeated measures ANOVA; Bonferroni *post hoc*	*P*<0.001. ICH+ESK20 vs. ICH+vehicle,*p*<0.001 ICH + vehicle vs. Sham,*p*<0.001
Figure 1F (Rotarod test on days 0,1,3 and 7)		Non-Normal distribution	Equal variance	Friedman test; Dunn's test *post hoc*	*P*<0.001. ICH+ESK20 vs. ICH+vehicle,*p*<0.001 ICH + vehicle vs. Sham,*p*<0.001
Figure 1F (Beam walking test on days 0,1,3 and 7)		Normal distribution	Equal variance	Repeated measures ANOVA; Bonferroni *post hoc*	*P*<0.001. ICH+ESK20 vs. ICH+vehicle,*p*<0.001 ICH + vehicle vs. Sham,*p*<0.001
Figure 1F (Grid walking test on days 0,1,3 and 7)		Normal distribution	Equal variance	Repeated measures ANOVA; Bonferroni *post hoc*	*P*<0.001. ICH+ESK20 vs. ICH+vehicle,*p*<0.001 ICH + vehicle vs. Sham,*p*<0.001

Figure 2C (NeuN/GAPDH)	Sham,n=4<br>ICH+Vehicle,n=4<br>ICH+ESK20,n=4	Normal distribution	Equal variance	One-way ANOVA; Bonferroni *post hoc*	*p*<0.001.<br>ICH+ESK20 vs. ICH+vehicle,*p*<0.001<br>ICH+vehicle vs. Sham,*p*=0.021
Figure 2C (GFAP/GAPDH)		Normal distribution	Equal variance	One-way ANOVA; Bonferroni *post hoc*	*p*<0.001.<br>ICH+ESK20 vs. ICH+vehicle,*p*=0.047<br>ICH+vehicle vs. Sham,*p*<0.001
Figure 2C (NSE/GAPDH)		Normal distribution	Unequal variance	Kruskal-Wallis test;<br>Dunn's test *post hoc*	*p*<0.001.<br>ICH+ESK20 vs. ICH+vehicle,*p*=0.016<br>ICH+vehicle vs. Sham,*p*=0.138
Figure 2C (MBP/GAPDH)		Non-Normal distribution	Unequal variance	Kruskal-Wallis test;<br>Dunn's test *post hoc*	*p*<0.001.<br>ICH+ESK20 vs. ICH+vehicle,*p*=0.046<br>ICH+vehicle vs. Sham,*p*=0.016
Figure 4A (NTF3/Tubulin)	Sham,n=4<br>6h,n=4<br>12h,n=4<br>D1,n=4<br>D3,n=4<br>D7,n=4	Normal distribution	Unequal variance	Kruskal-Wallis test;<br>Dunn's test *post hoc*	*p*=0.001.<br>Sham vs. 6h,*p*=1.000<br>Sham vs. 12h,*p*=1.000<br>Sham vs. D1,*p*=1.000<br>Sham vs. D3,*p*=0.046<br>Sham vs. D7,*p*=0.442

Figure 4E (NTF3/Tubulin)	Sham,n=4<br>ICH+Vehicle,n=4<br>ICH+ESK20,n=4	Normal distribution	Equal variance	One-way ANOVA; Bonferroni *post hoc*	*p*=0.002.<br>ICH+ESK20 vs. ICH+vehicle,*p*=0.050<br>ICH+vehicle vs. Sham,*p*=0.002
Figure 4E (BDNF/Tubulin)		Normal distribution	Unequal variance	Kruskal-Wallis test; Dunn's test *post hoc*	*p*<0.001.<br>ICH+ESK20 vs. ICH+vehicle,*p*=0.049<br>ICH+vehicle vs. Sham,*p*<0.001
Figure 4E (p-TrkB/TrkB)		Normal distribution	Equal variance	One-way ANOVA; Bonferroni *post hoc*	*p*<0.001.<br>ICH+ESK20 vs. ICH+vehicle,*p*<0.001<br>ICH+vehicle vs. Sham,*p*<0.001
Figure 4E (p-TrkC/TrkC)		Normal distribution	Equal variance	One-way ANOVA; Bonferroni *post hoc*	*p*<0.001.<br>ICH+ESK20 vs. ICH+vehicle,*p*=0.007<br>ICH+vehicle vs. Sham,*p*=0.004
Figure 5A (Weight Ratio on days 0,1,3 and 7)	Sham,n=6<br>ICH+Vehicle,n=6<br>ICH+ESK20,n=6<br>ICH+ESK20+AAV-NC,n=6<br>ICH+ESK20+AAV-ShNTF3,n=6	Normal distribution	Equal variance	Repeated measures ANOVA; Bonferroni *post hoc*	*P*<0.001.<br>ICH+vehicle vs. Sham,*p*<0.001<br>ICH+ESK20 vs. ICH+vehicle,*p*=0.002<br>ICH+ESK20+AAV-ShNTF3

					vs. ICH+ESK20,*p*<0.001
Figure 5B (Neurologic deficit score on days 0,1,3 and 7)		Normal distribution	Equal variance	Repeated measures ANOVA; Bonferroni *post hoc*	*P*<0.001. ICH+vehicle vs. Sham,*p*<0.001 ICH+ESK20 vs. ICH+vehicle,*p*<0.001 ICH+ESK20+AAV-ShNTF3 vs. ICH+ESK20,*p*<0.001
Figure 5C (Rotarod test on days 0,1,3 and 7)		Non-Normal distribution	Equal variance	Friedman test; Dunn's test *post hoc*	*P*<0.001. ICH+vehicle vs. Sham,*p*<0.001 ICH+ESK20 vs. ICH+vehicle,*p*<0.001 ICH+ESK20+AAV-ShNTF3 vs. ICH+ESK20,*p*<0.001
Figure 5C (Beam walking test on days 0,1,3 and 7)		Normal distribution	Equal variance	Repeated measures ANOVA; Bonferroni *post hoc*	*P*<0.001. ICH+vehicle vs. Sham,*p*<0.001 ICH+ESK20 vs. ICH+vehicle,*p*<0.001 ICH+ESK20+AAV-ShNTF3 vs. ICH+ESK20,*p*<0.001

Figure 5C (Grid walking test on days 0,1,3 and 7)		Non-Normal distribution	Equal variance	Friedman test; Dunn's test *post hoc*	*P*<0.001. ICH+vehicle vs. Sham,*p*<0.001 ICH+ESK20 vs. ICH+vehicle,*p*<0.001 ICH+ESK20+AAV-ShNTF3 vs. ICH+ESK20,*p*<0.001
Figure 5D (NTF3/Tubulin)	Sham,n=4 ICH+Vehicle,n=4 ICH+ESK20,n=4 ICH+ESK20+AAV-NC,n=4 ICH+ESK20+AAV-ShNTF3,n=4	Normal distribution	Equal variance	One-way ANOVA; Bonferroni *post hoc*	*p*<0.001. ICH+vehicle vs. Sham,*p*<0.001 ICH+ESK20 vs. ICH+vehicle,*p*<0.001 ICH+ESK20+AAV-ShNTF3 vs.ICH+ESK20,*p*<0.001
Figure 5D (PI3K/Tubulin)		Normal distribution	Equal variance	One-way ANOVA; Bonferroni *post hoc*	*p*<0.001. ICH+vehicle vs. Sham,*p*<0.001 ICH+ESK20 vs. ICH+vehicle,*p*<0.001 ICH+ESK20+AAV-ShNTF3 vs.ICH+ESK20,*p=0.002*
Figure 5D (p-AKT/AKT)		Normal distribution	Equal variance	One-way ANOVA; Bonferroni *post hoc*	*p*<0.001. ICH+vehicle vs. Sham,*p*=0.019 ICH+ESK20 vs. ICH+vehicle,*p*<0.001

					ICH+ESK20+AAV-ShNTF3 vs.ICH+ESK20,*p*<0.001
Figure 5D (p-GSK3B/GSK3B)		Normal distribution	Equal variance	One-way ANOVA; Bonferroni *post hoc*	*p*<0.001. ICH+vehicle vs. Sham,*p*=0.310 ICH+ESK20 vs. ICH+vehicle,*p*<0.001 ICH+ESK20+AAV-ShNTF3 vs.ICH+ESK20,*p*=0.046
Figure 5D (p-SGK1/SGK1)		Normal distribution	Equal variance	One-way ANOVA; Bonferroni *post hoc*	*p*<0.001. ICH+vehicle vs. Sham,*p*=0.007 ICH+ESK20 vs. ICH+vehicle,*p*<0.001 ICH+ESK20+AAV-ShNTF3 vs.ICH+ESK20,*p*<0.001
Figure 6A (Weight Ratio on days 0,1 and 3)	Sham,n=6 ICH+Vehicle,n=6 ICH+ESK20,n=6 ICH+ESK20+DMSO,n=6 ICH+ESK20+LY294002,n=6	Non-Normal distribution	Equal variance	Friedman test; Dunn's test *post hoc*	*P*<0.001. ICH+vehicle vs. Sham,*p*<0.001 ICH+ESK20 vs. ICH+vehicle,*p*<0.001 ICH+ESK20+LY294002 vs. ICH+ESK20,*p*<0.001

Figure 6B<br>(Neurologic deficit score<br>on days 0,1 and 3)		Non-Normal distribution	Equal variance	Friedman test; Dunn's test *post hoc*	$P<0.001$.<br>ICH+vehicle vs. Sham,$p<0.001$<br>ICH+ESK20 vs. ICH+vehicle,$p<0.001$<br>ICH+ESK20+LY294002 vs. ICH+ESK20,$p<0.001$
Figure 6C<br>(Rotarod test<br>on days 0,1 and 3)		Normal distribution	Equal variance	Repeated measures ANOVA; Bonferroni *post hoc*	$P<0.001$.<br>ICH+vehicle vs. Sham,$p<0.001$<br>ICH+ESK20 vs. ICH+vehicle,$p<0.001$<br>ICH+ESK20+LY294002 vs. ICH+ESK20,$p<0.001$
Figure 6C<br>(Beam walking test<br>on days 0,1 and 3)		Normal distribution	Equal variance	Repeated measures ANOVA; Bonferroni *post hoc*	$P<0.001$.<br>ICH+vehicle vs. Sham,$p<0.001$<br>ICH+ESK20 vs. ICH+vehicle,$p<0.001$<br>ICH+ESK20+LY294002 vs. ICH+ESK20,$p<0.001$
Figure 6C<br>(Grid walking test<br>on days 0,1 and 3)		Non-Normal distribution	Equal variance	Friedman test; Dunn's test *post hoc*	$P<0.001$.<br>ICH+vehicle vs. Sham,$p<0.001$<br>ICH+ESK20 vs. ICH+vehicle,$p<0.001$

					ICH+ESK20+LY294002 vs. ICH+ESK20,*p*<0.001
Figure 6D (NTF3/Tubulin)	Sham,n=4<br>ICH+Vehicle,n=4<br>ICH+ESK20,n=4<br>ICH+ESK20+AAV-NC,n=4<br>ICH+ESK20+AAV-ShNTF3,n=4	Normal distribution	Unequal variance	Kruskal-Wallis test;<br>Dunn's test *post hoc*	*p*=0.005.<br>ICH+vehicle vs. Sham,*p*=0.001<br>ICH+ESK20 vs. ICH+vehicle,*p*=0.036<br>ICH+ESK20+LY294002 vs.ICH+ESK20,*p*=1.000
Figure 6D (PI3K/Tubulin)		Normal distribution	Equal variance	One-way ANOVA;<br>Bonferroni *post hoc*	*p*<0.001.<br>ICH+vehicle vs. Sham,*p*<0.001<br>ICH+ESK20 vs. ICH+vehicle,*p*<0.001<br>ICH+ESK20+LY294002 vs.ICH+ESK20,*p*<0.001
Figure 6D (p-AKT/AKT)		Normal distribution	Equal variance	One-way ANOVA;<br>Bonferroni *post hoc*	*p*<0.001.<br>ICH+vehicle vs. Sham,*p*<0.001<br>ICH+ESK20 vs. ICH+vehicle,*p*<0.001<br>ICH+ESK20+LY294002 vs.ICH+ESK20,*p*<0.001

Figure 6D (p-GSK3B/GSK3B)		Non-Normal distribution	Unequal variance	Kruskal-Wallis test; Dunn's test *post hoc*	*p*=0.001. ICH+vehicle vs. Sham,*p*=0.040 ICH+ESK20 vs. ICH+vehicle,*p*<0.044 ICH+ESK20+LY294002 vs.ICH+ESK20,*p*<0.031
Figure 6D (p-SGK1/SGK1)		Normal distribution	Equal variance	One-way ANOVA; Bonferroni *post hoc*	*p*<0.001. ICH+vehicle vs. Sham,*p*<0.001 ICH+ESK20 vs. ICH+vehicle,*p*<0.001 ICH+ESK20+LY294002 vs.ICH+ESK20,*p*<0.001
Figure S2A (Weight Ratio on days 0,1,3,7 and 14)	Sham,n=6 ICH+Vehicle,n=6 ICH+ESK10,n=6 ICH+ESK20,n=6 ICH+ESK40,n=6	Normal distribution	Equal variance	Repeated measures ANOVA; Bonferroni *post hoc*	*P*<0.001. ICH+ESK20 vs. ICH+vehicle,*p*<0.001 ICH + vehicle vs. Sham,*p*<0.001
Figure S2B (Neurologic deficit score on days 0,1,3,7 and 14)		Normal distribution	Equal variance	Repeated measures ANOVA; Bonferroni *post hoc*	*P*<0.001. ICH+ESK20 vs. ICH+vehicle,*p*<0.001 ICH + vehicle vs. Sham,*p*<0.001

Figure S2C (Rotarod test on days 0,1,3,7 and 14)		Non-Normal distribution	Equal variance	Friedman test; Dunn's test *post hoc*	*P*<0.001. ICH+ESK20 vs. ICH+vehicle,*p*<0.001 ICH + vehicle vs. Sham,*p*<0.001
Figure S2C (Beam walking test on days 0,1,3,7 and 14)		Normal distribution	Equal variance	Repeated measures ANOVA; Bonferroni *post hoc*	*P*<0.001. ICH+ESK20 vs. ICH+vehicle,*p*<0.001 ICH + vehicle vs. Sham,*p*<0.001
Figure S2C (Grid walking test on days 0,1,3,7 and 14)		Non-Normal distribution	Equal variance	Friedman test; Dunn's test *post hoc*	*P*<0.001. ICH+ESK20 vs. ICH+vehicle,*p*<0.001 ICH + vehicle vs. Sham,*p*<0.001
Figure S3A (Weight Ratio on days 0,1,3,7 and 14)	Sham,n=3 Sham+ESK20,n=3	Normal distribution	Equal variance	Repeated measures ANOVA; Bonferroni *post hoc*	*P*=0.546
Figure S3B (Neurologic deficit score on days 0,1,3 and 7)		Normal distribution	Equal variance	Repeated measures ANOVA; Bonferroni *post hoc*	*P*=1

Figure S3C (Rotarod test on days 0,1,3,7 and 14)		Normal distribution	Equal variance	Repeated measures ANOVA; Bonferroni *post hoc*	*P*=0.805
Figure S3C (Beam walking test on days 0,1,3,7 and 14)		Normal distribution	Equal variance	Repeated measures ANOVA; Bonferroni *post hoc*	*P*=1
Figure S3C (Grid walking test on days 0,1,3 and 7)		Normal distribution	Equal variance	Repeated measures ANOVA; Bonferroni *post hoc*	*P*=0.855
Figure S4B (BDNF/Tubulin)	Sham,n=4<br>6h,n=4<br>12h,n=4<br>D1,n=4<br>D3,n=4<br>D7,n=4	Normal distribution	Equal variance	One-way ANOVA; Bonferroni *post hoc*	*p*<0.001.<br>Sham vs. 6h,*p*<0.001<br>Sham vs. 12h,*p*<0.001<br>Sham vs. D1,*p*<0.001<br>Sham vs. D3,*p*<0.001<br>Sham vs. D7,*p*<0.001

Figure S4C (PI3K/Tubulin)		Normal distribution	Equal variance	One-way ANOVA; Bonferroni *post hoc*	$p<0.001$.<br>Sham vs. 6h, $p<0.001$<br>Sham vs. 12h, $p<0.001$<br>Sham vs. D1, $p<0.001$<br>Sham vs. D3, $p<0.001$<br>Sham vs. D7, $p<0.001$
Figure S4D (p-AKT/AKT)		Normal distribution	Equal variance	One-way ANOVA; Bonferroni *post hoc*	$p<0.001$.<br>Sham vs. 6h, $p<0.001$<br>Sham vs. 12h, $p<0.001$<br>Sham vs. D1, $p<0.001$<br>Sham vs. D3, $p<0.001$<br>Sham vs. D7, $p<0.001$
Figure S5A (NTF3 mRNA expression)	Sham,n=4<br>AAV-ShNTF3-1,n=4<br>AAV-ShNTF3-2,n=4<br>AAV-ShNTF3-3,n=4	Normal distribution	Equal variance	One-way ANOVA; Bonferroni *post hoc*	$p<0.001$.<br>AAV-ShNTF3-1 vs. Sham, $p<0.001$<br>AAV-ShNTF3-2 vs. Sham, $p<0.001$<br>AAV-ShNTF3-3 vs. Sham, $p<0.001$<br>AAV-ShNTF3-2 vs. AAV-ShNTF3-1, $p<0.001$<br>AAV-ShNTF3-3 vs.AAV-ShNTF3-2, $p<0.001$

Figure S5A (NTF3 protein expression)		Normal distribution	Unequal variance	Kruskal-Wallis test; Dunn's test *post hoc*	p<0.001. AAV-ShNTF3-1 vs. Sham,p=0.005 AAV-ShNTF3-2 vs. Sham,p=1.000 AAV-ShNTF3-3 vs. Sham,p=0.048 AAV-ShNTF3-2 vs. AAV-ShNTF3-1,p=0.260 AAV-ShNTF3-3 vs.AAV-ShNTF3-2,p=*1.000*
