# Supplementary figures and images for: Esketamine Provides Neuroprotection After Intracerebral Hemorrhage in Mice via the NTF3/PI3K/AKT Pathway
Source: CNS Neurosci Ther. 2024 Dec 17;30(12):e70145. doi: 10.1111/cns.70145 (PMC11652676; doi:10.1111/cns.70145)

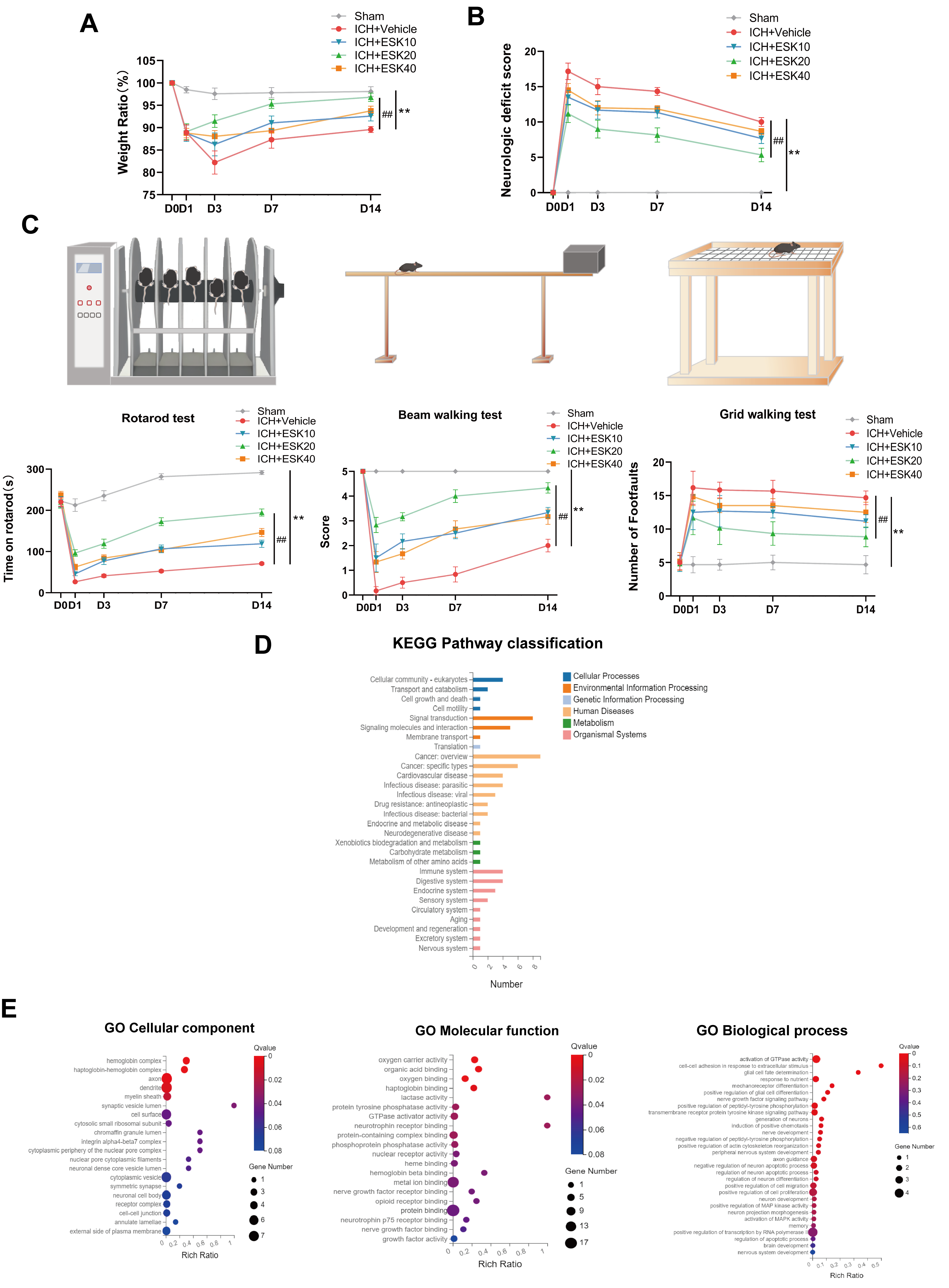

Supplement: Supplementary file 1 — Data S1. [file CNS-30-e70145-s001.zip › Supplementary Figure 2.tif]
