# Supplementary material for: Esketamine Provides Neuroprotection After Intracerebral Hemorrhage in Mice via the NTF3/PI3K/AKT Pathway
Source: CNS Neurosci Ther. 2024 Dec 17;30(12):e70145. doi: 10.1111/cns.70145 (PMC11652676; doi:10.1111/cns.70145)
Supplement: Supplementary file 2 — Table S1. [file CNS-30-e70145-s006.docx]

| \| **Table S1 Summary of experimental groups, sample sizes, and mortality rate in the study** \| \| \| \| \| \| \| \| \| \| \| \| --- \| --- \| --- \| --- \| --- \| --- \| --- \| --- \| --- \| --- \| --- \| \| **Experimental Groups** \| **Weight  Neurological test Behavior test** \| **Brain water content** \| **Injury volume** \| **FJC TUNEL** \| **RNA-seq** \| **IF** \| **WB** \| **Mortality** \| **Subtotal** \| \| **Experiment 2** \|  \|  \|  \|  \|  \|  \|  \|  \|  \| \| Sham \| 6 \| 6 \| 6 \| 3 \|  \| 3 \| 5 \| 0 \| 29 \| \| ICH+Vehicle(normal saline) \| 6 \| 6 \| 6 \| 3 \|  \| 3 \| 5 \| 6 \| 35 \| \| ICH+Esketamine(10mg/kg) \| 6 \|  \|  \|  \|  \|  \|  \| 3 \| 9 \| \| ICH+Esketamine(20mg/kg) \| 6 \| 6 \| 6 \| 3 \|  \| 3 \| 5 \| 1 \| 30 \| \| ICH+Esketamine(40mg/kg) \| 6 \|  \|  \|  \|  \|  \|  \| 4 \| 10 \| \| Sham+Esketamine(20mg/kg) \| 3 \|  \|  \|  \|  \|  \|  \|  \| 3 \| \| Sham+Esketamine(40mg/kg) \| 3 \|  \|  \|  \|  \|  \|  \|  \| 3 \| \| **Experiment 3** \|  \|  \|  \|  \|  \|  \|  \|  \| 0 \| \| Sham \|  \|  \|  \|  \| 3 \|  \|  \| 0 \| 3 \| \| ICH+Vehicle(normal saline) \|  \|  \|  \|  \| 3 \|  \|  \| 1 \| 4 \| \| ICH+Esketamine(20mg/kg) \|  \|  \|  \|  \| 3 \|  \|  \| 0 \| 3 \| \| **Experiment 4** \|  \|  \|  \|  \|  \|  \|  \|  \| 0 \| \| Sham \| 6 \|  \|  \|  \|  \| 3 \| 5 \| 0 \| 14 \| \| ICH+Vehicle(normal saline) \| 6 \|  \|  \|  \|  \| 3 \| 5 \| 1 \| 15 \| \| ICH+Esketamine(20mg/kg) \| 6 \|  \|  \|  \|  \| 3 \| 5 \| 0 \| 14 \| \| **Experiment 5** \|  \|  \|  \|  \|  \|  \|  \|  \| 0 \| \| Sham \| 6 \|  \|  \|  \|  \| 3 \| 5 \| 0 \| 14 \| \| ICH+Vehicle(normal saline) \| 6 \|  \|  \|  \|  \| 3 \| 5 \| 2 \| 16 \| \| ICH+Esketamine(20mg/kg) \| 6 \|  \|  \|  \|  \| 3 \| 5 \| 1 \| 15 \| \| ICH+ESK20+AAV-NC \| 6 \|  \|  \|  \|  \| 3 \| 5 \| 1 \| 15 \| \| ICH+ESK20+AAV-ShNTF3 \| 6 \|  \|  \|  \|  \| 3 \| 5 \| 2 \| 16 \| \| **Experiment 6** \|  \|  \|  \|  \|  \|  \|  \|  \| 0 \| \| Sham \| 6 \|  \|  \|  \|  \| 3 \| 5 \| 0 \| 14 \| \| ICH+Vehicle(normal saline) \| 6 \|  \|  \|  \|  \| 3 \| 5 \| 2 \| 16 \| \| ICH+Esketamine(20mg/kg) \| 6 \|  \|  \|  \|  \| 3 \| 5 \| 0 \| 14 \| \| ICH+ESK20+DMSO \| 6 \|  \|  \|  \|  \| 3 \| 5 \| 0 \| 14 \| \| ICH+ESK20+LY294002 \| 6 \|  \|  \|  \|  \| 3 \| 5 \| 3 \| 17 \| \| **Total** \| 114 \| 18 \| 18 \| 9 \| 9 \| 48 \| 80 \| 27 \| 323 \| \| ICH, intracerebral hemorrhage. DMSO, Dimethyl sulfoxide. ESK20, Esketamine(20mg/kg). \| \| \| \| \| \| \| \| \| \| \| |
| --- | --- | --- | --- | --- | --- | --- | --- | --- | --- | --- | --- | --- | --- | --- | --- | --- | --- | --- | --- | --- | --- | --- | --- | --- | --- | --- | --- | --- | --- | --- | --- | --- | --- | --- | --- | --- | --- | --- | --- | --- | --- | --- | --- | --- | --- | --- | --- | --- | --- | --- | --- | --- | --- | --- | --- | --- | --- | --- | --- | --- | --- | --- | --- | --- | --- | --- | --- | --- | --- | --- | --- | --- | --- | --- | --- | --- | --- | --- | --- | --- | --- | --- | --- | --- | --- | --- | --- | --- | --- | --- | --- | --- | --- | --- | --- | --- | --- | --- | --- | --- | --- | --- | --- | --- | --- | --- | --- | --- | --- | --- | --- | --- | --- | --- | --- | --- | --- | --- | --- | --- | --- | --- | --- | --- | --- | --- | --- | --- | --- | --- | --- | --- | --- | --- | --- | --- | --- | --- | --- | --- | --- | --- | --- | --- | --- | --- | --- | --- | --- | --- | --- | --- | --- | --- | --- | --- | --- | --- | --- | --- | --- | --- | --- | --- | --- | --- | --- | --- | --- | --- | --- | --- | --- | --- | --- | --- | --- | --- | --- | --- | --- | --- | --- | --- | --- | --- | --- | --- | --- | --- | --- | --- | --- | --- | --- | --- | --- | --- | --- | --- | --- | --- | --- | --- | --- | --- | --- | --- | --- | --- | --- | --- | --- | --- | --- | --- | --- | --- | --- | --- | --- | --- | --- | --- | --- | --- | --- | --- | --- | --- | --- | --- | --- | --- | --- | --- | --- | --- | --- | --- | --- | --- | --- | --- | --- | --- | --- | --- | --- | --- | --- | --- | --- | --- | --- | --- | --- | --- | --- | --- | --- | --- | --- | --- | --- | --- | --- | --- | --- | --- | --- | --- | --- | --- | --- | --- | --- | --- | --- | --- | --- | --- | --- | --- | --- | --- | --- | --- | --- | --- | --- | --- | --- | --- | --- | --- | --- | --- | --- | --- | --- | --- | --- | --- | --- | --- | --- | --- | --- | --- | --- | --- | --- | --- | --- | --- | --- | --- | --- | --- | --- | --- |
