# Supplementary material for: Esketamine Provides Neuroprotection After Intracerebral Hemorrhage in Mice via the NTF3/PI3K/AKT Pathway
Source: CNS Neurosci Ther. 2024 Dec 17;30(12):e70145. doi: 10.1111/cns.70145 (PMC11652676; doi:10.1111/cns.70145)
Supplement: Supplementary file 3 — Table S2. [file CNS-30-e70145-s005.docx]

| **Table S2 Differential gene expression levels** | | |
| --- | --- | --- |
| GeneSymbol | log2 (ICH+ESK20/ICH) | Qvalue (ICH+ESK20/ICH) |
| Gm40365 | 8.999534542 | 6.52724E-07 |
| LCT | 6.008777802 | 7.07079E-05 |
| NTF3 | 3.395613114 | 0.013784383 |
| CXCL9 | 3.015654997 | 0.037655887 |
| GBP10 | 2.739852271 | 0.024109354 |
| TAFA1 | 1.808695209 | 0.007196684 |
| HBA-a1 | 1.740663499 | 0.000203535 |
| HBB-bs | 1.47190688 | 0.000238499 |
| NR4A3 | 1.445573143 | 0.033477756 |
| NWD2 | 1.424563015 | 0.000959826 |
| TNFRSF25 | 1.420486914 | 0.028900198 |
| HBA-a2 | 1.240869874 | 0.042027434 |
| LAG3 | 1.019516697 | 0.002140105 |
| NECTIN3 | 1.013688211 | 0.013784383 |
| FLT4 | -1.21686994 | 0.046182544 |
|  |  |  |
|  |  |  |
