# Supplementary material for: Esketamine Provides Neuroprotection After Intracerebral Hemorrhage in Mice via the NTF3/PI3K/AKT Pathway
Source: CNS Neurosci Ther. 2024 Dec 17;30(12):e70145. doi: 10.1111/cns.70145 (PMC11652676; doi:10.1111/cns.70145)
Supplement: Supplementary file 4 — Table S3. [file CNS-30-e70145-s002.docx]

| **Table S3 Prediction of relevant signaling pathways and key genes** | | |
| --- | --- | --- |
| **Term** | **FDR** | **Genes** |
| Neurotrophin signaling pathway | 1.36759E-15 | NTRK1, NGFR, NTRK2, SHC3, SHC1, SORT1, BDNF, CAMK2A, FRS2, PTPN11, NGF, NTF4, NTF3, PLCG1 |
| Ras signaling pathway | 1.34527E-13 | NTRK1, NGFR, NTRK2, SHC3, SHC1, BDNF, RASGRF1, PTPN11, NGF, GRIN2B, GRIN1, NTF4, GRIN2A, NTF3, PLCG1 |
| Calcium signaling pathway | 0.000356814 | NTRK1, NTRK2, GRIN2A, CAMK2A, PLCG1, NOS1, NGF, GRIN1 |
| Cocaine addiction | 0.000512041 | GRIN2A, BDNF, DLG4, GRIN2B, GRIN1 |
| Alcoholism | 0.000602472 | NTRK2, GRIN2A, SHC3, SHC1, BDNF, GRIN2B, GRIN1 |
| MAPK signaling pathway | 0.000666495 | NTRK1, NTF4, NTRK2, NGFR, BDNF, RASGRF1, NTF3, NGF |
| Rap1 signaling pathway | 0.000826998 | NGFR, GRIN2A, SRC, PLCG1, NGF, GRIN2B, GRIN1 |
| ErbB signaling pathway | 0.002254438 | SHC3, SHC1, SRC, CAMK2A, PLCG1 |
| Circadian entrainment | 0.003123911 | GRIN2A, CAMK2A, NOS1, GRIN2B, GRIN1 |
| Inflammatory mediator regulation of TRP channels | 0.003123911 | NTRK1, SRC, CAMK2A, PLCG1, NGF |
| Pathways of neurodegeneration - multiple diseases | 0.006768514 | GRIN2A, BDNF, DLG4, CAMK2A, PLCG1, NOS1, GRIN2B, GRIN1 |
| Natural killer cell mediated cytotoxicity | 0.006768514 | SHC3, SHC1, PTPN11, FYN, PLCG1 |
| PI3K-Akt signaling pathway | 0.00760173 | NTRK1, NTF4, NTRK2, NGFR, BDNF, NTF3, NGF |
| Phospholipase D signaling pathway | 0.01002471 | SHC3, SHC1, PTPN11, FYN, PLCG1 |
| Long-term potentiation | 0.01002471 | GRIN2A, CAMK2A, GRIN2B, GRIN1 |
| Amphetamine addiction | 0.010233409 | GRIN2A, CAMK2A, GRIN2B, GRIN1 |
| Drug metabolism - cytochrome P450 | 0.010890871 | CYP2C9, CYP2B6, CYP2C19, CYP3A4 |
| Glioma | 0.011568624 | SHC3, SHC1, CAMK2A, PLCG1 |
| EGFR tyrosine kinase inhibitor resistance | 0.012721907 | SHC3, SHC1, SRC, PLCG1 |
| Axon guidance | 0.015799411 | SRC, CAMK2A, PTPN11, FYN, PLCG1 |
| Linoleic acid metabolism | 0.020529003 | CYP2C9, CYP2C19, CYP3A4 |
| Focal adhesion | 0.020529003 | SHC3, SHC1, SRC, RASGRF1, FYN |
| Proteoglycans in cancer | 0.021067013 | SRC, CAMK2A, FRS2, PTPN11, PLCG1 |
| Lipid and atherosclerosis | 0.023910536 | CYP2C9, CYP2B6, SRC, CAMK2A, PLCG1 |
| cAMP signaling pathway | 0.025298161 | GRIN2A, BDNF, CAMK2A, GRIN2B, GRIN1 |
| Glutamatergic synapse | 0.026164844 | GRIN2A, DLG4, GRIN2B, GRIN1 |
| Nicotine addiction | 0.031362694 | GRIN2A, GRIN2B, GRIN1 |
| Relaxin signaling pathway | 0.034163085 | SHC3, SHC1, SRC, NOS1 |
|  |  |  |
