# Supplementary material for: Esketamine Provides Neuroprotection After Intracerebral Hemorrhage in Mice via the NTF3/PI3K/AKT Pathway
Source: CNS Neurosci Ther. 2024 Dec 17;30(12):e70145. doi: 10.1111/cns.70145 (PMC11652676; doi:10.1111/cns.70145)
Supplement: Supplementary file 5 — Table S4. [file CNS-30-e70145-s004.docx]

| **Table S4 Blood routine results of mice** | | | | |
| --- | --- | --- | --- | --- |
| **Index** | **Sham** | **Sham+ESK20** | **ICH+Vehicle** | **ICH+ESK20** |
| WBC（10^9^/L） | 5.37±0.42 | 6.77±1.07 | 3.53±2.14 | 3.27 ±1.93 |
| Lymph（10^9^/L） | 4.37±0.25 | 5.33±1 | 2.93±1.87 | 2.60 ±1.54 |
| Mon（10^9^/L） | 0.17±0.12 | 0.23±0.06 | 0.1±0.1 | 0.07 ±0.06 |
| Gran（10^9^/L） | 0.83±0.15 | 1.2±0.2 | 0.5±0.2 ** | 0.60 ±0.35 |
| Lymph(%) | 81.63±4.02 | 78.23±2.87 | 78.7±8.93 | 79.13 ±2.66 |
| Mon(%) | 3.33±1.97 | 3.9±1.66 | 4.17±1.01 | 2.70 ±0.20 |
| Gran(%) | 15.03±2.06 | 17.87±1.96 | 17.13±8.92 | 18.17 ±2.80 |
| RBC(10^12^/L) | 7.1±0.38 | 6.93±0.61 | 6.45±0.36 | 6.69 ±0.19 |
| HGB(g/L) | 104.33±5.51 | 105±10 | 99.33±6.35 | 103.00 ±2.00 |
| HCT(%) | 31.03±1.48 | 29.53±3 | 27.77±1.55 | 28.87 ±0.42 |
| MCV(fL) | 43.8±1.08 | 42.67±0.57 | 43.13±0.5 | 43.27 ±0.64 |
| MCH(pg) | 14.63±1.17 | 15.07±0.12 | 15.33±0.15 | 15.37 ±0.21 |
| MCHC（g/L） | 336±19.31 | 355±2.65 | 357±4.58 | 356.33 ±2.31 |
| RDW(%) | 15.83±1.07 | 15.7±1.4 | 15.9±1.67 | 14.60 ±1.23 |
| PLT（10^9^/L） | 493.67±89.03 | 656.33±92.97 | 703.33±124.74 | 733.67 ±172.26 |
| MPV(fL) | 5.07±0.21 | 4.8±0.17 | 4.87±0.12 | 4.77 ±0.15 |
| PDW | 16.1±0.3 | 15.63±0.15 | 15.67±0.23 | 15.57± 0.12 |
| PCT(%) | 0.25±0.05 | 0.31±0.03 | 0.34±0.07 | 0.35 ±0.09 |
| * and ** represent significant difference and highly significant differences between the ICH+Vehicle group and the Sham+ESK20 group | | | | |
