# Supplementary material for: Esketamine Provides Neuroprotection After Intracerebral Hemorrhage in Mice via the NTF3/PI3K/AKT Pathway
Source: CNS Neurosci Ther. 2024 Dec 17;30(12):e70145. doi: 10.1111/cns.70145 (PMC11652676; doi:10.1111/cns.70145)
Supplement: Supplementary file 6 — Table S5. [file CNS-30-e70145-s003.docx]

| **Table S5 Blood biochemical results of mice** | | | | |
| --- | --- | --- | --- | --- |
| **Index** | **Sham** | **Sham+ESK20** | **ICH+Vehicle** | **ICH+ESK20** |
| ALT（U/L） | 44.58 ± 8.00 | 45.28 ± 3.91 | 51.93 ± 19.19 | 46.19 ± 12.8 |
| AST（U/L） | 133.72 ± 38.86 | 134.50 ± 10.62 | 176.42 ± 59.53 | 136.89 ± 44.8 |
| D-BIL（umol/l） | 9.44 ± 1.09 | 12.94 ± 6.63 | 16.22 ± 4.46 | 7.47 ± 3.72 |
| T-BIL（umol/l） | 18.38 ± 4.62 | 17.88 ± 9.50 | 23.28 ± 1.58 * | 11.53 ± 3.36 |
| ALB（g/L） | 27.08 ± 2.01 | 24.73 ± 1.42 | 26.05 ± 1.30 | 24.82 ± 0.21 |
| ALP（U/L） | 124.89 ± 22.39 | 85.03 ± 7.19 | 82.87 ± 12.65 | 73.59 ± 6.83 |
| GGT（U/L） | 0.87 ± 0.06 | 0.89 ± 0.09 | 0.89 ± 0.09 | 0.94 ± 0.01 |
| TBA（umol/l） | 3.16 ± 1.24 | 4.12 ± 1.67 | 4.87 ± 0.61 | 3.23 ± 1.83 |
| BUN（mg/dL） | 26.06 ± 3.01 | 23.22 ± 0.76 | 22.75 ± 3.97 | 23.27 ± 0.96 |
| CR（umol/l） | 21.60 ± 3.61 | 27.68 ± 12.53 | 27.23 ± 11.36 | 21.16 ± 3.24 |
| UA（umol/l） | 130.89 ± 35.41 | 158.68 ± 53.01 | 200.90 ± 27.05 | 143.58 ± 42.47 |
| * and ** represent significant difference and highly significant differences between the ICH+Vehicle group and the ICH+ESK20 group | | | | |
